# Efficient strategy for alleviating neuronal apoptosis and oxidative stress damage of Alzheimer's disease through dual targeting BCL-2 gene promoter i-motif and β-amyloid

**DOI:** 10.1016/j.redox.2025.103600

**Published:** 2025-03-18

**Authors:** Dongsheng Ji, Jiahui Zhang, Jihai Liang, Zhi-Shu Huang, Bing Shu, Ding Li

**Affiliations:** a School of Pharmaceutical Sciences, Sun Yat-sen University, Guangzhou University, City, 132 Waihuan East Road, Guangzhou, 510006, PR China; b School of Pharmacy, Guangdong Pharmaceutical University, Guangzhou, PR China

**Keywords:** Alzheimer's disease, BCL-2, I-motif, β-amyloid (Aβ), Oxidative stress, anti-Apoptosis

## Abstract

Alzheimer's disease (AD) is a severe neurodegenerative disorder characterized by abnormal metabolism of β-amyloid (Aβ) precursor proteins and neuronal apoptosis, ultimately leading to cognitive dysfunction. The pathogenesis of AD is complex, and current single-target therapies are not effective in preventing the rapid progression of AD, which highlights the urgent need for developing multi-target drugs. In this study, a series of compounds were synthesized through a multi-targeting ligand strategy. After extensive screening and evaluation, we found a lead compound **B14**, which showed excellent dual targeting ability for effectively alleviating neuronal apoptosis and oxidative stress damage of AD. In our molecular and cellular level experiments, **B14** could target and stabilize the i-motif structure formed on the *BCL-2* promoter to upregulate *BCL-2* expression, which could also bind to Aβ and inhibit its deposition. In the Aβ_1-42_-induced cell model, **B14** could maintain mitochondrial function and number, regulate intracellular reactive oxygen species (ROS) and Ca^2+^ metabolism disorders, and effectively reduce Aβ_1-42_-induced apoptosis. Further studies showed that **B14** also exhibited good ability to cross the blood-brain barrier (BBB), which significantly improved learning memory and cognitive deficits, reduced brain Aβ plaques, alleviated inflammation and restored oxidative stress markers in APP/PS1 mice. Our findings provide an innovative strategy of dual targeting *BCL-2* promoter i-motif for transcriptional regulation and Aβ aggregation synergistically for mitigating AD pathologies. **B14** represents a promising multi-target lead compound with a good potential for further development for AD treatment.

## Highlights

• We prepared novel compound **B14** targeting *BCL2* i-motif and Aβ for Alzheimer's disease.
• **B14** showed protection through mitochondria apoptotic pathway and up-regulated BCL2.
• **B14** could improve cognition, learning and memory on APP/PS1 transgenic mice.
• **B14** could reduce apoptosis and improve pathological lesions on brain of APP/PS1 mice.
• **B14** could improve inflammation and oxidative stress damage on brain of APP/PS1 mice.

## Graphical abstract

**Figure:**
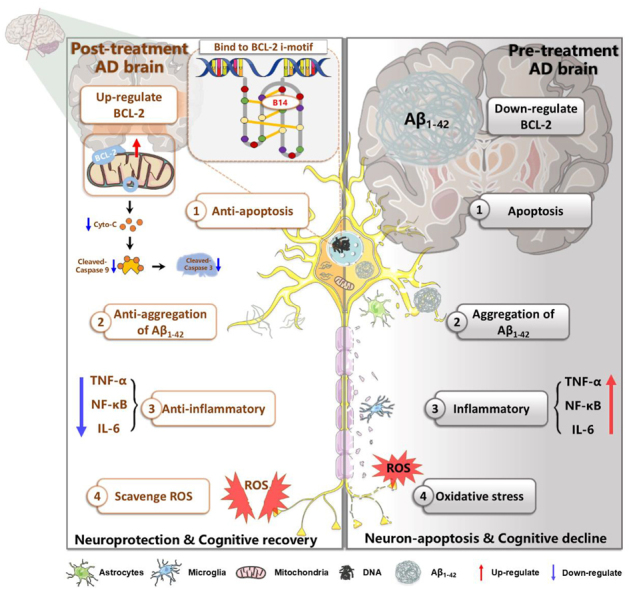

## 1 Introduction

Alzheimer's disease (AD), also known as senile dementia, has become the fourth leading cause of death after heart disease, malignant tumor, and stroke. Its incidence rate is rising with the increase of people's life expectancy. AD is a chronic neurodegenerative disease characterized by memory loss, cognitive decline, and motor disorders. As a multifactorial disease, the pathogenesis of AD is complex. Although the exact pathogenesis is still unclear, numerous studies have shown that abnormal metabolism of β-amyloid (Aβ) precursor proteins [1], hyperphosphorylation of Tau proteins [2], cholinergic damage [3], oxidative stress damage [4], neuronal cell apoptosis [5], mitochondrial dysfunction [6], neuroinflammation [7], genetics [8], the environment [9], and extracellular vehicles (EVs) carrying chemical messages for intercellular communication [10], may play important roles in the pathogenesis of AD. Over the past few decades, the FDA has approved three classes of AD therapeutics: acetylcholinesterase (AChE) inhibitors (tacrine, donepezil, rivastigmine and galantamine) [11], N-methyl-d-aspartate (NMDA) receptor antagonists (memantine) [12] and Aβ antibody (such as aduhelm) [13,14]. However, the effects of these drugs are unsatisfactory, which could only relieve some symptoms but could not cure AD or even slow down its progression. Therefore, the development of innovative and multi-target directed anti-AD drugs could provide new solution for AD treatment.

Aβ deposition and formation of senile plaques are major pathological changes in AD. These peptides spontaneously self-aggregate into ordered forms of β-folded sheets, monomers, oligomers, protofibrils, and amyloid plaques [15]. Amyloid plaques are deposited extracellularly in central nervous system (CNS), triggering a pathogenic cascade of reactions that induce oxidative stress and synaptic dysfunction [16,17], and promote the formation of neuronal tangles, thereby exacerbate neuronal death. Thus, inhibition of Aβ aggregation and reduction of amyloid plaque toxicity are major strategies for the treatment of AD. Various natural product derivatives with benzofuran structure have shown a wide range of biological activities including Aβ anti-aggregation activity [[18], [19], [20], [21], [22], [23], [24], [25], [26]]. In addition, various studies have demonstrated a significant association between AD and the expression of the anti-apoptotic factor BCL-2 [[27], [28], [29]]. A reduced BCL-2 expression first activates the apoptosis-related factors Caspase9 and Caspase3, which leads to intracellular Ca^2+^ disruption and triggers mitochondria-mediated endogenous apoptotic pathway causing neuronal apoptosis, thus leading to AD [30]. On the other hand, upregulation of BCL-2 expression can effectively ameliorate AD [[31], [32], [33]]. Previous studies have demonstrated that mall molecules could upregulate *BCL-2* transcription and translation after stabilizing the i-motif structure formed by C-rich sequences in the upstream region of the *BCL-2* gene promoter, which could possibly provide new avenues for AD therapy and relevant drug development [[34], [35], [36]]. However, for treatment of AD, a single target appears to be insufficient due to its complex pathogenesis and multiple pathologic manifestations.

In this study, we hypothesized that the dual-targeting strategy of inhibiting Aβ deposition and upregulating *BCL-2* expression could jointly act on multiple pathways to effectively slow down AD progression. To verify this hypothesis, we designed and developed a series of acridone-benzofuran derivatives with acridone as the i-motif binding backbone [[37], [38]] using a multi-target directed ligand strategy [[39], [40]], with addition of benzofuran as the Aβ inhibitory moiety [[26], [41], [42]]. This innovative strategy could simultaneously target on both Aβ toxicity and apoptosis dysregulation while exploring the potential of i-motif-targeting approach for AD therapy. The biological activities of these acridone-benzofuran derivatives were evaluated by using various experiments. Our results showed that the introduction of benzofuran group enhanced Aβ inhibitory property, and the presence of acridone backbone maintained good binding affinity to *BCL-2* gene promoter i-motif structure (Fig. S1C). Further biological experiments showed that derivative **B14** could up-regulate the expression of BCL-2, inhibit the aggregation of Aβ_1-42_ and reduce oxidative stress, which could consequently mitigate neuronal apoptosis and delay AD progression. This method of simultaniously inhibiting Aβ_1-42_ aggregation and up-regulating gene expression by small molecules targeting the gene promoter i-motif could become a practical method for AD treatment. This revealed for the first time the physiological function and importance of the i-motif structure as a potential target for AD treatment, which provided new strategy and possible solution for its clinical drug development.

## 2 Material and methods

### 2.1 Oligonucleotides, Aβ_1-42_ and compounds

DNA oligonucleotides were purchased from Sangon (Shanghai, China) as salt-free oligomers, which were then dissolved in relevant buffers, with their sequences as shown in Table S1. Their concentrations were based on single-strand DNA concentrations, which were determined according to their absorbance at 260 nm using a NanoDrop 1000 Spectrophotometer (Thermo Scientific, USA), and calculated based on their respective molar extinction coefficients. Their further dilutions to working concentrations were made with relevant buffers [37,38,43] as follows:

BPES buffer: 30 mM (KH_2_PO_4_, K_2_HPO_4_), 1 mM EDTA, and 100 mM KCl
MES buffer: 20 mM MES, 100 mM KCl, pH 5.5
Tris–HCl buffer: 50 mM Tris–HCl, 100 mM KCl, pH 7.4

Aβ_1-42_ was purchased from Gill Biochemicals Ltd (Shanghai, China). 0.5 mg of Aβ_1-42_ oligomer or monomer was dissolved with 22 μL DMSO, followed by addition of 1.086 mL of phenol red-free MEM/F12 or ddH_2_O to make a 100 μM Aβ_1-42_ reservoir, and stored in −80 °C freezer. Our synthetic compounds available for screening have structures as shown in Tables S3 and S4. Compounds were dissolved in DMSO at 10 mM concentration, and stored in −20 °C freezer.

### 2.2 Thioflavine T (ThT) assay

The experiment was performed following standardized amyloid detection protocols without significant modification [44]. Curcumin (Aladdin, Shanghai, China), a natural inhibitor of Aβ deposition, was used as a positive control with ThT (Aladdin, Shanghai, China) as a fluorescent indicator. 30 μL of freshly formulated Aβ_1-42_ (30 μM) was incubated without or with **A1-16**, **B1-14**, Curcumin at concentration of 40 μM for 24h. Then, it was mixed with 400 μL of freshly formulated ThT (15 μM), which was incubated in the dark for 30 min. Fluorescence signals were recorded using a fluorescence spectrophotometer, and changes in Aβ fibre deposition were determined through the strength of the fluorescence signals. The scanning conditions were set as follows: excitation wavelength at 440 nm, emission wavelength at 480 nm, and slit width of 20 nm.

### 2.3 Congo red binding assay

A total of 30 μM Congo red (Aladdin, Shanghai, China) solution (20 mM potassium phosphate, 50 mM NaCl, pH 7.4) was prepared and filtered with a 0.45 μm filter. The Aβ_1-42_ solutions (30 μM) were co-incubated with **B14** (20 μM), **A22** (20 μM), or Curcumin (20 μM) at 37 °C protected from light for 72h. The Aβ_1-42_ solution was mixed with equal volume of Congo red solution, followed by incubation for 30 min. A spectral region from 400 nm to 650 nm was measured by using a UV–vis spectrophotometer (AoE, China).

### 2.4 Circular dichroism (CD) experiment

The circular dichroism (CD) experiment was performed to investigate the effect of **B14** (20 μM) or Curcumin (20 μM) on the change of Aβ_1-42_ secondary structure. The Aβ_1-42_ solutions (100 μM) were co-incubated with **B14** (20 μM) or Curcumin (20 μM) at 37 °C for 72h. Ellipticity changes of Aβ_1-42_ were monitored employing a Chirascan CD spectropolarimeter (Chirascan, England).

### 2.5 Thioflavine T fluorescence spectroscopy assay

The Aβ_1-42_ solutions (100 μM) were co-incubated with **B14** (20 μM) or Curcumin (20 μM) at 37 °C protected from light for 96 h. 30 μL of Aβ_1-42_ solution was taken at different time points (0 h, 10 h, 24 h, 48 h, 72 h, 96 h), mixed fully with 400 μL of ThT solution (15 μM), and incubated in the dark for 30 min. The fluorescence signal was recorded by using a Cary Eclipse fluorescence spectrophotometer (Varian, USA) with the following setting: excitation wavelength at 440 nm, emission wavelength at 480 nm, and slit width of 20 nm.

### 2.6 Dot blot

Dot blot assays were performed to detect Aβ_1-42_ fibril aggregation with **B14** or Curcumin as described previously with minor modification [45]. Briefly, Aβ_1-42_ was diluted into 20 μM with buffer (20 mM HEPES, 150 mM NaCl, pH 7.4), and then incubated without or with **B14** or Curcumin at 20 μM concentration. After incubation at 37 °C for 24 h, 10 μL aliquots of 20 μM Aβ_1-42_ reaction mixtures were spotted onto nitrocellulose membranes. Membranes were blocked for 2 h with 5 % non-fat milk in TBS. After washing, membranes were incubated with the anti-Aβ fibril antibody (1:1,000, CST, USA) dissolved in TBS containing 3 % bovine serum albumin (BSA, Beyotime, Jiangsu, China) and 0.01 % Tween-20 (Solarbio, Beijing, China), and developed using an alkaline-phosphatase anti-rabbit secondary antibody (1:5,000, Proteintech, China). The bands were then detected using an enhanced chemiluminescence (ECL) kit (Millipore, USA).

### 2.7 Transmission electron microscopy (TEM)

TEM experiment was carried out following a published method without significant modification [46], and the Aβ_1-42_ solution (100 μM) was co-incubated with **B14** (20 μM) or Curcumin (20 μM) at 37 °C for 72h. The samples were dropped to a carbon-coated grid, stained with 2 % phosphotungstic acid, and then dried at room temperature. The morphology of Aβ_1-42_ fiber was observed on a JEM-1200EX II transmission electron microscope (Jeol, Japan).

### 2.8 Surface plasmon resonance (SPR) experiment

SPR measurement was performed on a ProteOn XPR36 protein interaction array system (Bio-Rad Laboratories, Hercules, California, USA) using a Neutravidin-coated GLH sensor chip. *BCL-2* i-motif binding analysis was performed following a published method without significant modification [47]. For immobilization, 5′-biotin-labeled DNA oligomers at 800 nM annealed in 1 × MES buffer at pH 5.5 by heating at 95 °C for 5 min, were attached to a reptavidin-coated sensor chip. Compounds were prepared through serial dilution with flowing buffer (20 mM MES, 100 mM KCl, pH 5.5 for i-motif) in at least 6 concentrations, which were simultaneously injected at a flow rate of 30 mL/min for a 120 s of association phase, followed by 250 s of dissociation phase at 25 °C. Langmuir model was used for fitting kinetic data, which was analyzed with ProteOn manager software. *K*_D_ values were determined through equation *K*_D_ = kd/ka. Compound A22 was used as a positive control, and other compounds were selected as candidates only if their *K*_D_ values for binding to *BCL-2* i-motif were similar to that of A22.

### 2.9 Microscale thermophoresis (MST) experiment

In the MST experiment of DNA with small molecule, 5′-FAM-labeled DNA oligomer (1 μM) was annealed in 1 × BPES buffer at pH 5.5 by heating at 95 °C for 5 min. The concentration of compound was initially 50 μM, which was half-diluted each time for 15 times with 1XBPES buffer. After incubation of DNA with compound for 60 min, the sample was loaded in MST-grade glass capillaries. The intensity of the LED power was set at 20 %, and the MST laser was set at 40 %. The analyses were performed using a Monolith NT.115, and the fitting curve was obtained by using NT Analysis 1.5.41 via Hill fitting. The *K*_D_ value represents the numeric equivalent of the concentration of **B14** when the response is half of the plateau response (R_max_) of the fitting curve.

### 2.10 Circular dichroism (CD) melting experiment

CD-melting experiment was carried out following a published method without significant modification [48]. *BCL-2* promoter i-motif and G-quadruplex structures at a concentration of 5 μM were annealed in buffer at 95 °C for 10 min, and then cooled to room temperature. The annealed samples were incubated with **B14** for 6 h, and then molar ellipticity at 287 nm for i-motif structure or 260 nm for G-quadruplex structure, where the CD value was the highest in our experiment, was measured over a temperature range of 20–90 °C at a heating rate of 0.5 °C/min. Tm values were determined and calculated through nonlinear regression curve fitting with software GraphPad Prism 9.0. (GraphPad Software Inc., California, USA).

### 2.11 ESI-MS

The oligomer for *BCL-2* promoter i-motif at concentration of 5 μM was annealed in 1 × BPES buffer with or without 20 μM **B14** at 95 °C for 5 min, followed by cooling to room temperature. Mass spectrometry signals were collected by using ESI-MS (Thermo, LCQ DECA Plus XP) and analyzed by using pro-mass.

### 2.12 Cell culture

SH-SY5Y cells (Procell, Wuhan, China) were routinely maintained at 37 °C in a humidified atmosphere with 5 % CO_2_ using MEM/F12 with 10 % heat-inactivated FBS (Bio-Channel, Nanjing, China), penicillin (100 U/mL, Thermo, Guangzhou, China) and streptomycin (100 lg/mL, Thermo, Guangzhou, China). Once the cultures reached 70–80 % confluence, the cells were subcultured with 0.05 % trypsin-ethylenediaminetetraacetic acid (EDTA, New Cell Molecular, Suzhou, China) at the required split ratio.

### 2.13 Fluorescence imaging of intracellular Aβ_1-42_ accumulation

SH-SY5Y cells were seeded in a 96-well plate (6000 cells/well) in 100 μL medium. After the cells adhered to the wall, SH-SY5Y cells were incubated with serum-free medium containing 25 μM Aβ_1-42_ for 6 h to allow for the uptake of Aβ_1-42_ into the cells [49]. Then solutions of **B14** at different concentrations were added to the cells, with the mixtures incubated for 48 h. After being washed for three times with pre-cooled PBS, the cells were fixed with freshly prepared 4 % paraformaldehyde (Servicebio, Guangzhou, China) for 10 min at room temperature. After being washed for three times with pre-cooled PBS, the cells were incubated with ThT solution for 20 min at 25 °C in the dark. After washing for three times with pre-cooled PBS, the cells were stained with 10 μg/mL Hoechst 33342 solution (Aladdin, Shanghai, China) for 20 min at 25 °C in the dark. After washing for three times with pre-cooled PBS, the stained cells were observed and photographed with a FV3000 (Olympus, Japan) fluorescence microscope.

### 2.14 Reverse transcription and real-time PCR

SH-SY5Y cells were treated with or without **B14**, and total RNA was subsequently isolated and purified. Reverse transcription was performed using a cDNA synthesis kit. The relative transcription levels of the genes were evaluated through quantitative real-time PCR using the SYBR Green-based method, as described in the Supplementary Date. Details regarding the primer sequences and the raw data can be found in Table S7 and Table S8.

### 2.15 Western blot

Protein samples (25–40 μg) were separated using 10 % SDS-PAGE and subsequently transferred onto PVDF membranes (0.22 μm, Servicebio, Guangzhou, China) via electro transfer. Following blocking with TBST solution (Servicebio, Guangzhou, China) containing 5 % BSA (Solarbio, Beijing, China) for approximately 60 min at room temperature, the membrane was exposed to various primary antibodies overnight at 4 °C on a rotating shaker. Then, the membranes were incubated with HRP-conjugated secondary antibody (Affinity, Changzhou, China) for 2 h. Protein bands were then detected using an ECL kit. The signal intensity of each target protein was quantified using Image J 1.8.0 software (Bio-Rad Laboratories, California, USA).

### 2.16 Measurement of *in vitro* BBB transport

bEnd.3 (10 × 10^4^ cells/well) was cultured in the upper chamber of a 12 wells-plate within a transwell system [50]. The transepithelial electrical resistance (TEER) was measured to monitor the tightness of the monolayer, and the monolayer of cells with a minimum of 200 Ω cm^2^ was used for further experiments. The levels of donepezil and **B14** in the lower chamber were evaluated at 467 nm based on a standard curve via UV–Vis spectrophotometry. The degree of transporting across this BBB model was measured using the following equation (1):

(1) $$\text{Permeability}\;\left(\%\right)=\frac{Co-Cs}{Co}\times 100\%$$

with *Co* and *Cs* representing the concentrations of donepezil and **B14** in the upper chamber before and after incubation, respectively.

### 2.17 Animals

Five months old male APP/PS1 mice, weighing 25–33 g, were purchased from Guangdong Medical Laboratory Animal Center and housed in the Laboratory Animal Center of Sun Yat-sen University (Guangzhou, China) for experimentation. All procedures were approved by the Animal Care and Use Committee of Sun Yat-sen University and complied with legal requirements and national guidelines for the care and maintenance of laboratory animals (Approval No. SYSU-IACUC-2024-002032). The mice were housed in a specific pathogen-free environment with a controlled temperature of 22 ± 1 °C and a 12-h dark/light cycle. The mice were fed with a standard diet, and 30 mice were randomly divided into 6 groups (N = 5). Compound **B14** was dissolved in saline and administered at doses of 10, 20, or 40 mg/kg. APP/PS1 male mice were grown until 6 months of age, and then injected intraperitoneally with compound **B14** every other day for 60 days. Behavioral experiments were then conducted, and tissue specimens were collected for biochemical analysis.

### 2.18 Behavioral experiments

The spatial memory and learning abilities of the mice were assessed using Morris water maze (MWM), novel object recognition (NOR) and nesting experiments (NBT). The anxiety and depressive behaviors were evaluated through the open field test (OFT) and elevated plus maze (EPM). Detailed experimental procedures are provided in the supplementary information.

### 2.19 Hematoxylin and Eosin (H&E) staining and Nissl staining

The mice were sacrificed, with their brains and organs isolated and embedded in paraffin. The brains and organs were then sectioned into 6 μm thin slices. These slices were subjected to H&E or Nissl staining (Servicebio, Guangzhou, China). The stained sections were subsequently imaged and analyzed by using microscopy.

### 2.20 Transmission electron microscope (TEM) of hippocampus microstructure

After the behavioral tests, the mice were anesthetized and perfused with 0.1 M pre-cooled phosphate-buffered saline for 5 min, followed by 4 % pre-cooled paraformaldehyde until the mice were stiff. The hippocampi were then extracted and fixed in 3 % pre-cooled glutaraldehyde solution for 6 h, cut into 0.5–1 mm^3^ blocks, and post-fixed in 1 % osmium tetroxide for 60 min. The samples were then dehydrated in acetone, embedded in epoxy resin, and sectioned into ultrathin slices. These sections were stained with lead citrate and uranyl acetate, followed by visualization using a JEM-1200EXII (Jeol, Japan) TEM.

### 2.21 Biochemical parameters analysis

The hippocampi were homogenized in normal saline containing protease inhibitors. The homogenate was centrifuged at 2500 rpm for 10 min, and the protein concentration in the supernatant was measured by using the bicinchoninic acid (BCA, Beyotime, Jiangsu, China) method. The levels of malondialdehyde (MDA) and the activities of superoxide dismutase (SOD), glutathione peroxidase (GSH-Px) and cathepsin (CAT) were determined by using relevant assay kits (Beyotime, Jiangsu, China).

### 2.22 Statistical analysis

Data were expressed as the mean ± SEM. Data between the two groups were analyzed with one-way ANOVA or unpaired Student's t-tests using GraphPad Prism9.0 (GraphPad Software Inc, California, USA). A ∗p value of ≤0.05 was considered to be statistically significant.

## 3 Results

### 3.1 Design, synthesis and screening of small molecules for binding to *BCL-2* gene promoter i-motif structure and inhibiting Aβ_1-42_ deposition

**Fig. 1:**
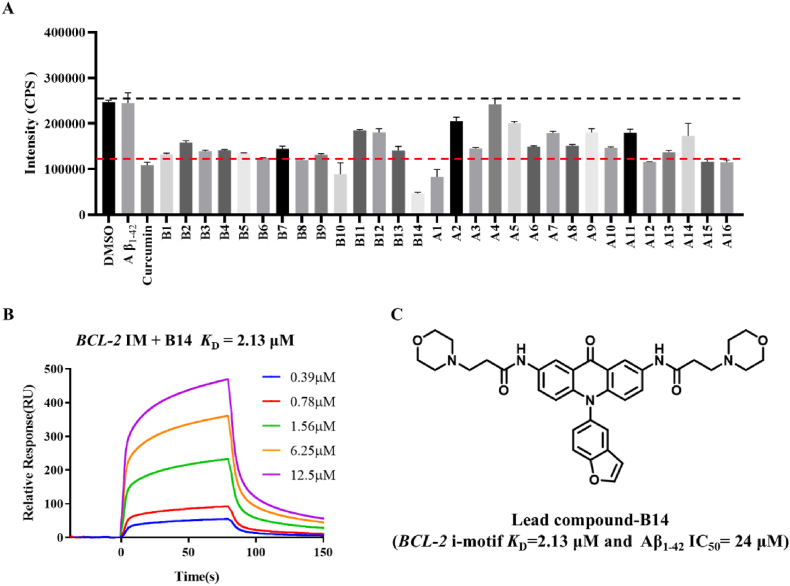
**B14** could specifically bind to *BCL-2* gene promoter i-motif (*BCL-2* IM) and inhibit Aβ_1-42_ deposition. (A) ThT fluorescence was measured after incubation of compounds with Aβ_1-42_. Data were statistically analyzed as means ± SEM. (B) *K*_*D*_ value for binding of **B14** to *BCL-2* IM was determined by using SPR. (C) Structure of compound **B14**.

As mentioned before, both BCL-2 and Aβ_1-42_ are potential targets for treatment of AD, and one single target appears to be insufficient. Therefore, present research was carried out to develop small molecules for both regulating *BCL-2* gene expression through its promoter i-motif and inhibiting Aβ_1-42_ aggregation simultaneously. We synthesized a total of 30 compounds as two series (A and B), through a bi-target directed strategy. Benzofuran as an Aβ deposition inhibitory moiety, was incorporated into acridone backbone, with amino side chains introduced at C-2 and C-7 positions for enhancing their binding affinity and specificity to i-motifs. These compounds were prepared through synthetic route as shown in Scheme S1, with their structures and purity determined by using ^1^H NMR, ^13^C NMR, HRMS, and HPLC, as detailed in supplementary data. Then, we screened our synthesized compounds by using ThT fluorescence and SPR experiment. ThT could specifically bind to the β-fold of proteins to enhance their fluorescence efficiency [44], and the inhibitory effects of compounds on Aβ aggregation could be assessed by analyzing changes in the fluorescence signals of ThT. Curcumin is a well-known natural inhibitor of Aβ self-aggregation [51,52], which served as a control compound in the present study. Our synthesized compounds all exhibited some Aβ inhibitory activities with the introduction of benzofuran ring, as shown in Fig. 1A and Table S2. As shown in Fig. S2, among these compounds, **B14** had the strongest inhibitory effect with 78.41 % inhibition of Aβ self-aggregation at 40 μM concentration. Our SPR experiment showed that compound **B14** also had a strong binding affinity to *BCL-2* gene promoter i-motif, with *K*_D_ value determined to be 2.13 μM as shown in Fig. 1B. We further confirmed that **B14** did have a strong binding affinity to *BCL-2* gene promoter i-motif by using FRET (Fig. S3) and TO substitution experiment (Fig. S4). Consequently, **B14** was selected for further investigation of its potential anti-AD activity.

### 3.2 Effect of B14 on the structural change of Aβ_1-42_ was analyzed using various methods

**Fig. 2:**
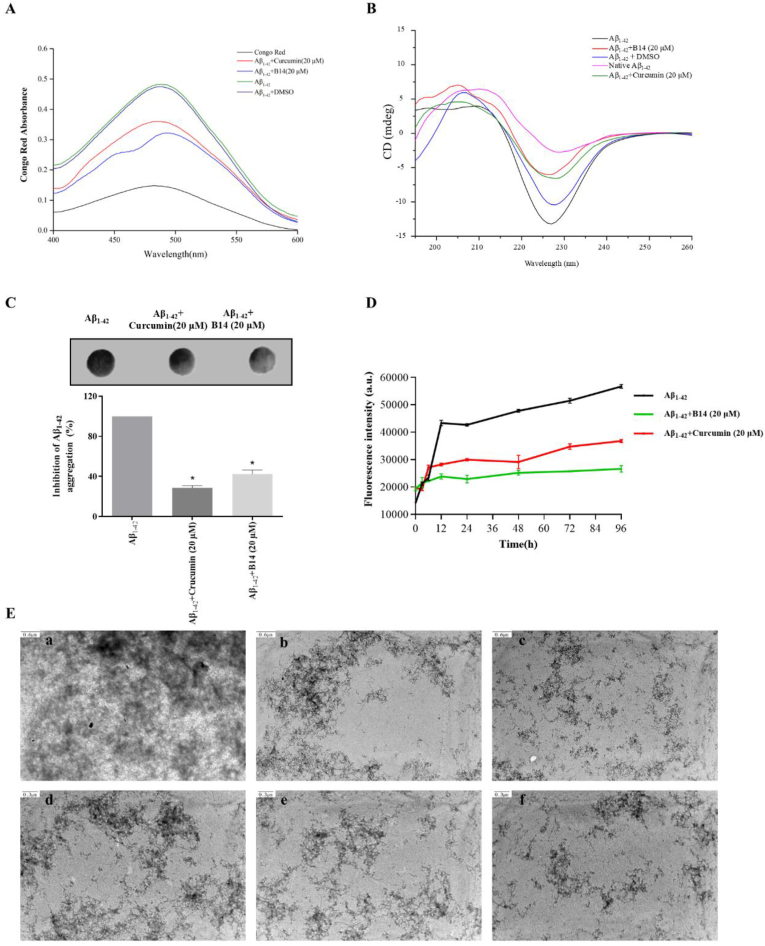
Effect of **B14** on Aβ_1-42_ aggregation with its structural change analyzed using various methods. (A) Congo red absorbance experiment. (B) CD experiment. (C) Dot blot experiment with quantitative analysis. (D) ThT experiment. (E) TEM image analysis of Aβ_1-42_ fibrils. (a,d) Aβ_1-42_ alone. (b,e) Aβ_1-42_ with Curcumin at 20 μM. (c,f) Aβ_1-42_ with **B14** at 20 μM. The scale bar of (a–c) was 0.6 μm, and the scale bar of (d–f) was 0.3 μm. Data were statistically analyzed as means ± SEM. ∗*p* < 0.05, vs. Aβ_1-42_ group.

Congo red is commonly used to detect specific conformations of beta-folded proteins [53,54], which was used to evaluate the effect of **B14** on Aβ_1-42_ accumulation in amyloid fibres in the present study. As shown in Fig. 2A, the absorbance of Congo red in the Aβ_1-42_ sample was significantly enhanced after 72 h of incubation, which suggested the formation of a hydrophobic structure in the Aβ_1-42_ sample. The absorbance was significantly reduced after incubation with **B14** (20 μM). In comparison, Curcumin (20 μM) also showed some inhibitory effect, but much lower than **B14**. Circular dichroism (CD) as an instrument for rapid analysis of protein secondary structure [46,55], was used to assess the effect of **B14** on the secondary structure of Aβ_1-42_. As shown in Fig. 2B, after incubation of Aβ_1-42_ alone for 3 days, its CD spectrum showed a characteristic negative peak at 226 nm, indicating a secondary structure of Aβ_1-42_. After treatment with **B14** (20 μM), the CD signal intensity of Aβ_1-42_ was much decreased, with its characteristic peaks more significantly reduced than those of Aβ_1-42_ treated with 20 μM Curcumin. Dot blot experiment was carried out with Aβ_1-42_ treated with different compounds, followed by analysis using an Aβ_1-42_ protofibril-specific antibody. As shown in Fig. 2C, incubation of Aβ_1-42_ with **B14** reduced the formation of Aβ fibers and greatly decreased Aβ self-aggregation, while Curcumin had a relatively less significant effect compared with **B14**. These experiments suggested that **B14** could affect the secondary structure of Aβ_1-42_, reduce its conversion into fibers, inhibit Aβ self-aggregation, and consequently reducing its toxicity.

ThT as a benzothiazole dye specifically binds to the β-fold in proteins to increase their fluorescence efficiency, which is mainly used to study the formation of fibrous substances [44]. The effect of **B14** on Aβ_1-42_ accumulation was investigated by analyzing changes in ThT fluorescence signals. As previously reported [56], the growth kinetic profile of Aβ_1-42_ in the control group for Aβ_1-42_ alone was semi-S-shaped as shown in Fig. 2D. After incubating Aβ_1-42_ with **B14** or Curcumin at 20 μM concentration for 96 h, the measured ThT fluorescence intensity decreased by 39.58 ± 2.08 % and 20.83 ± 1.68 % respectively, indicating that **B14** had a significant binding and consequently inhibitory effect on Aβ_1-42_ accumulation, which was consistent with our previous data. To visually evaluate the effect of **B14** on Aβ_1-42_ aggregation, the ultrastructure of Aβ_1-42_ fibers was analyzed by using transmission electron microscopy. After 72 h incubation of Aβ_1-42_ alone, Aβ_1-42_ aggregated to form many long bifurcated fibrillate structures as shown in Fig. 2E (a and d), which is a distinctive feature of amyloid aggregation [57]. The above experiment was perform with addition of **B14** at 20 μM concentration, and Aβ_1-42_ was found to be fragmented, while formation of protofibrils or amorphous oligomers was significantly reduced (Fig. 2E–c and f). These results showed that **B14** effectively inhibited the aggregation of Aβ_1-42_, which was also consistent with our previous experimental results.

### 3.3 Further studies on the interaction of B14 with *BCL-2* promoter i-motif

**Fig. 3:**
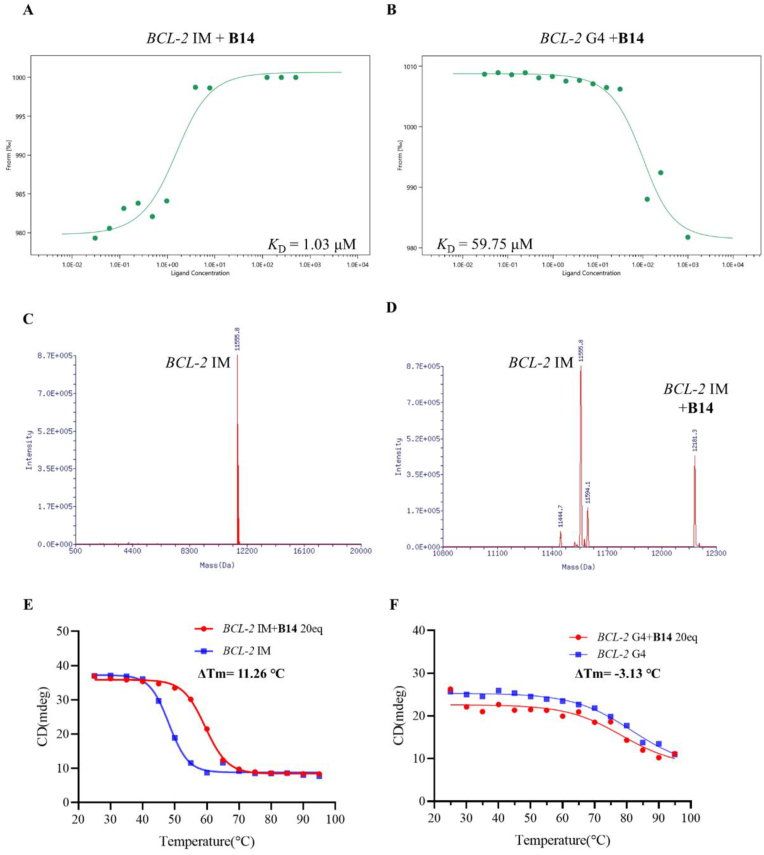
Effect of **B14** on *BCL-2* promoter DNA secondary structures. (A) *K*_D_ value was determined through MST for the binding of **B14** with *BCL-2* i-motif (*BCL-2* IM) in BPES buffer at pH 5.5. (B) *K*_D_ value was determined through MST for interaction of **B14** with *BCL-2* G-quadruplex (*BCL-2* G4) in Tris-HCl buffer at pH 7.4. (C) ESI-MS spectrum of oligomer for *BCL-2* i-motif annealed in 1 × BPES buffer at pH 6.5. (D) ESI-MS spectrum of oligomer for *BCL-2* i-motif with addition of **B14** at pH 6.5. The mass of 12,181.3 Da corresponding to *BCL-2* i-motif–**B14** adduct was detected. (E) CD melting experiment was performed for 2 μM *BCL-2* i-motif without and with 20 μM **B14** in BPES buffer at pH 5.5. (F) CD melting experiment was performed for 2 μM *BCL-2* G-quadruplex without and with 20 μM **B14** in Tris-HCl buffer at pH 7.4.

It has been shown that acridone derivatives have significant effects on gene promoter DNA secondary structures, and therefore we investigated whether **B14** could interact with these structures. Surface plasmon resonance (SPR) and microscale thermophoresis (MST) experiments were performed to evaluate the interaction of **B14** with various gene promoter DNA secondary structures, including *BCL-2* IM for *BCL-2* promoter i-motif and *BCL-2* G4 for *BCL-2* promoter G-quadruplex. In SPR experiments, the *K*_D_ values were determined for binding of **B14** to *ILPR* promoter i-motif (>50 μM), *HRAS* promoter i-motif (>50 μM), and *(G*_*2*_*C*_*4*_*)*_*6*_ promoter i-motif (33.30 μM), which were all significantly higher than that of *BCL-2* promoter i-motif (2.13 μM), as shown in Fig. 3A and B. The *K*_D_ value for binding of **B14** to *BCL-2* IM was determined to be 1.06 μM by using MST. It is known that FAM and TAMRA double-labeled C-rich oligonucleotides fluorescently quench upon folding into i-motif structures. To further understand the interaction of **B14** with *BCL-2* IM, we examined the effect of **B14** on the fluorescence response of double-labeled DNA by using fluorescence titration. As shown in Supplementary Figs. S2D and E, **B14** had a dose-dependent effect on the fluorescence of *BCL-2* IM, without significant impact on the fluorescence of other gene promoter i-motifs. This result was consistent with our TO displacement result as shown in Figs. S3A–C. The TO displacement ratio of *BCL-2* IM by **B14** was determined to be 65 %, compared with 16 % for *HRAS* promoter i-motif, 27 % for *RET* promoter i-motif, and only 1 % for *RB* promoter i-motif. The above results showed that **B14** could bind specifically to *BCL-2* IM.

In order to further understand the binding interaction of **B14** to *BCL-2* i-motif, we performed ESI-MS experiment. *BCL-2* i-motif was incubated with **B14** at pH 6.5, and the mixture was subjected to ESI-MS analysis. A peak of 12,181.3 Da corresponding to their adduct appeared on the mass spectrum, suggesting that **B14** could bind tightly to *BCL-2* i-motif (Fig. 3C and D), in contrast, no corresponding binding peak appeared for incubation of **B14** with *BCL-2* G4 (Figs. S5A and S5B), suggesting that **B14** could bind tightly to *BCL-2* IM with selectivity. Previously we have found that acridone derivative **A22** could alleviate liver damage of NAFLD/NASH by stabilizing *BCL-2* gene promoter i-motif and upregulating its expression. In the present study, we performed UV titration experiment for both **B14** and **A22** for comparison, as shown in Figs. S5C and D. The absorption titration spectra of **B14** were found to be similar to those of **A22**, and their absorption peaks at 269 nm gradually increased in dose-dependent manners with the increase of *BCL-2* i-motif concentration. These results suggested that **B14** could induce the formation of *BCL-2* i-motif and interact with *BCL-2* i-motif in a similar binding mechanism of **A22**. Our EMSA experiment showed that the oligomer for *BCL-2* promoter C-rich sequence tended to form free single-stranded DNA at pH 6.2, as shown in Fig. S5E. The gradual appearance of a relatively slow i-motif migration band with increasing concentrations of **B14** suggested that **B14** could induce *BCL-2* i-motif formation in a dose-dependent manner. An ideal i-motif ligand should have two basic characteristics including high binding specificity and high stabilization capacity. In order to know whether **B14** could stabilize *BCL-2* i-motif, we performed circular dichroism (CD) and CD-melting experiments. In the presence of **B14**, *BCL-2* i-motif showed an apparently enhanced absorption peak at 286 nm in CD spectra (Fig. S5F), suggesting that **B14** could induce the formation of *BCL-2* i-motif. CD melting experiment showed that addition of **B14** increased the melting temperature of *BCL-2* i-motif by 11.26 °C as shown in Fig. 3E. In contrast, the effect of **B14** on *BCL-2* G-quadruplex was found to be much weak, with a *ΔTm* value determined to be −3.13 °C as shown in Fig. 3F. These results demonstrated that **B14** could bind tightly to and stabilize *BCL-2* i-motif structure at conditions close to physiological pH.

### 3.4 Molecular modeling of compound B14 with Aβ fibril and *BCL-2* promoter i-motif structure

**Fig. 4:**
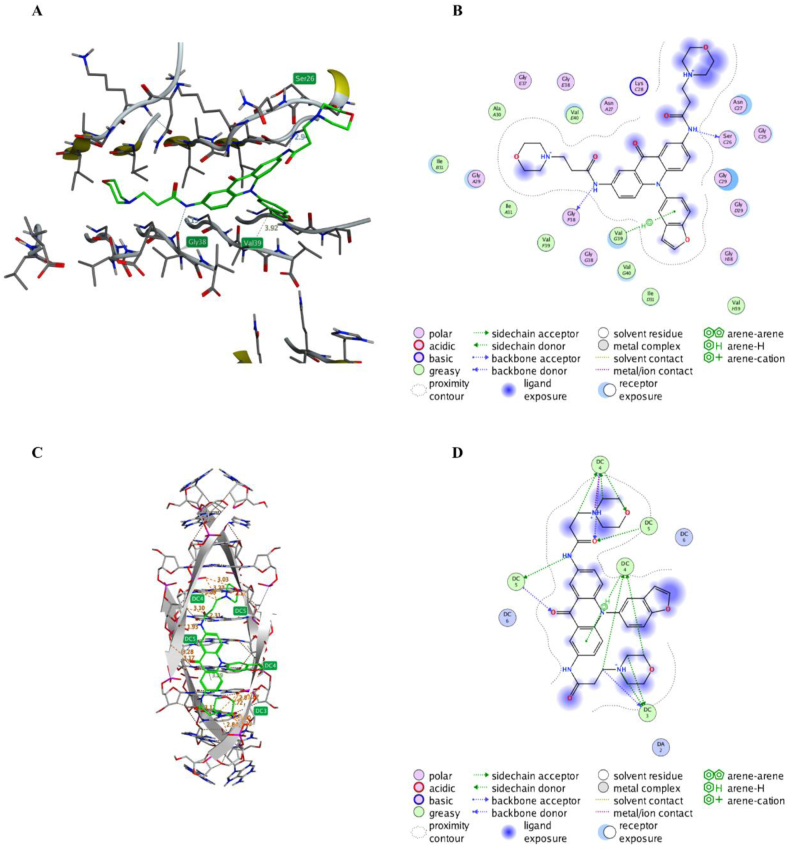
Hypothetical binding mode of **B14** to Aβ fibril and *BCL-2* promoter i-motif structure. (A&B) Graphic views for binding of **B14** to Aβ fibril (PDB ID: 2LMN). (C&D) Graphic views for binding of **B14** to a model tetrameric i-motif (PDB ID: 1YBL).

Molecular docking experiments were performed using Auto Dock 4.2 for possible interactions of compound **B14** with Aβ fibril (PDB: 2LMN) and *BCL-2* promoter i-motif structure. At the molecular level, a general symmetry exists between two fibril forms of Aβ. As shown in Fig. 4A and B, the benzofuran ring of **B14** could recognize to Aβ fibrils. The amino side chain on one side of **B14** formed a hydrogen bond with Ser26 of the β-folded fragment of one Aβ fibril, while the amino side chain on the other side formed a hydrogen bond with Gly38 of the non-β-folded fragment of the other Aβ fibril, thus exerting an inhibition of Aβ aggregation. Since the structure of *BCL-2* promoter i-motif has not been reported, we used the telomeric i-motif (PDB ID: 1YBL) as a model structure for the docking experiment. As shown in Fig. 4C and D, the acridone ring of **B14** interacted with the i-motif loop structure through hydrogen bonding interaction, with its side chain forming hydrogen bonds to the phosphodiester of the i-motif. These data well explained why **B14** could inhibit Aβ aggregation and tightly bind to *BCL-2* promoter i-motif structure.

### 3.5 B14 could inhibit intracellular accumulation of Aβ_1–42_ and up-regulate the transcription and translation of BCL-2 in SH-SY5Y cells

**Fig. 5:**
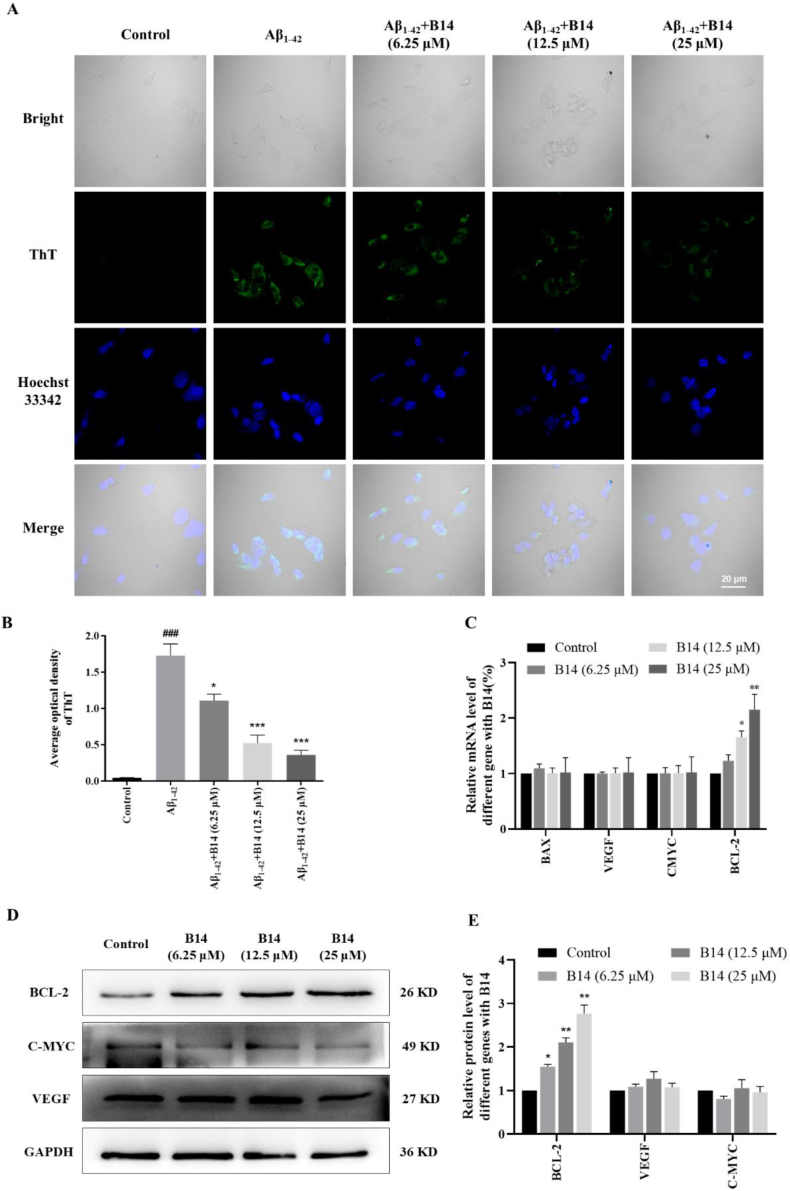
Effects of **B14** on intracellular accumulation of Aβ_1-42_. (A) ThT fluorescence images of the morphology of intracellular Aβ_1-42_ accumulation and (B) percentage of positive areas. (C) Effects of **B14** on BCL-2, BAX, VEGF and C-MYC gene transcription in SH-SY5Y cells were analyzed through cell incubation with increasing concentration of **B14**. (D) Effects of **B14** on BCL-2, C-MYC and VEGF protein expressions were analyzed through cell incubation with increasing concentration of **B14** for 24 h, which were quantitatively analyzed as shown in (E). All the experiments were repeated for three times. Dates were statistically analyzed as means ± SEM. ^###^*p* < 0.001, vs. Control group; ∗*p* < 0.05, vs. Aβ_1-42_ group; ∗∗∗*p* < 0.001, vs. Aβ_1-42_ group.

ThT has been one of the most commonly used indicators of amyloid fibrils due to its broad staining ability, exceptional sensitivity and ease of use [58,59]. To investigate the effect of **B14** on the intracellular aggregation of Aβ_1-42_, the fluorescence intensity of ThT in SH-SY5Y cells was measured through fluorescence analysis, and the aggregation of Aβ_1-42_ in the cells was determined based on the change of fluorescence intensity. As shown in Fig. 5A and B, the intensity of green fluorescence in the cells of the Aβ_1-42_ group was much enhanced compared with the control group, which indicated that Aβ_1-42_ was significantly aggregated in SH-SY5Y cells under the experimental condition. The fluorescence intensity of SH-SY5Y cells was significantly reduced in a dose-dependent manner upon addition of **B14**, indicating that Aβ_1-42_ aggregation was strongly inhibited by **B14**. Notably, the fluorescence of SH-SY5Y cells almost completely disappeared after treatment with **B14** at 25 μM concentration. This result further demonstrated that **B14** effectively inhibited the intracellular aggregation of Aβ_1-42_ at cellular level by using sensitive fluorescence method.

Since **B14** could strongly and selectively bind to and stabilize *BCL-2* promoter i-motif structure, we investigated whether **B14** could affect the transcription and translation of BCL-2 in SH-SY5Y cells. As shown in Table S6, **B14** exhibited minimal toxicity to most cell lines, with an IC_50_ value for SH-SY5Y cells determined to be more than 100 μM. SH-SY5Y cells were treated with increasing concentration of **B14**, and it was found that **B14** significantly up-regulated BCL-2 transcription in SH-SY5Y cells in a dose-dependent manner as shown in Fig. 5C. In comparison, **B14** had no significant effect on the transcription of other genes containing promoter i-motif structure, including BAX, VEGF, and C-MYC. It was found that **B14** could also up-regulate BCL-2 translation in SH-SY5Y cells in a dose-dependent manner without significant effect on C-MYC and VEGF expression as shown in Fig. 5D and E. These experimental data indicated that **B14** preferentially upregulated the transcription and translation of BCL-2 in SH-SY5Y cells possibly through selective interaction with *BCL-2* promoter i-motif structure.

### 3.6 Effect of B14 on the viability of SH-SY5Y cells incubated with Aβ_1-42_

**Fig. 6:**
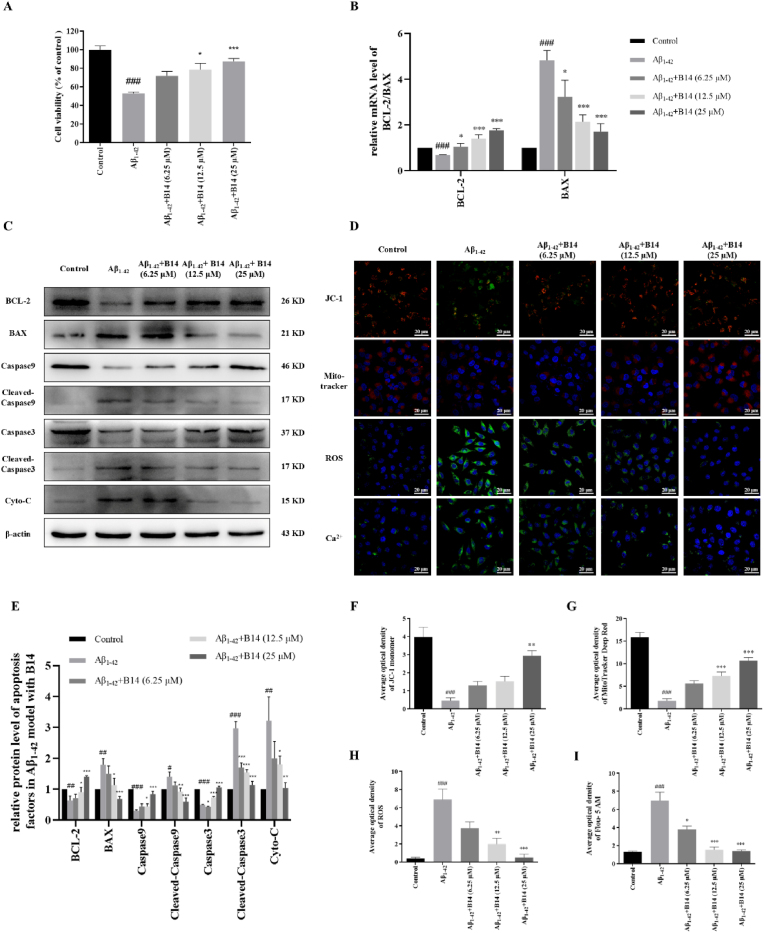
Anti-apoptotic effect of **B14** was studied in Aβ_1-42_ induced cell model. (A) Anti-apoptotic effect of **B14** on Aβ_1-42_ induced toxicity to SH-SY5Y cells was determined by using MTT. (B) Effects of **B14** on BCL-2 and BAX gene transcriptions in SH-SY5Y cells were analyzed through cell incubation with increasing concentration of **B14** in Aβ_1-42_ induced cell model. (C) Effects of **B14** on protein expressions related with apoptosis were analyzed through Western blotting, which were quantitatively analyzed as shown in (E). (D) JC-1, Mito-tracker red, ROS, and Ca^2+^ in SH-SY5Y cells incubated with Aβ_1-42_ were affected by increasing concentrations of **B14**, with their histogram as shown in (F–I). All the experiments were repeated for three times. The data were expressed as the mean ± SEM: ^#^p < 0.05, ^##^p < 0.01, ^###^p < 0.001 vs Control group; ∗p < 0.05, ∗∗p < 0.01, ∗∗∗p < 0.001 vs Aβ_1-42_ group, significantly different from the control.

Excessive neuronal cell apoptosis has been found to be associated with AD, and thus anti-neuronal cell apoptosis can block the pathological progression of AD [60,61]. Since **B14** could enhance BCL-2 expression and inhibit Aβ_1-42_ aggregation, we further investigated its anti-apoptotic effect in an Aβ_1-42_-induced neuronal cell apoptosis model. **B14** showed no significant toxicity to SH-SY5Y cells as shown in Fig. S7A. Apoptosis was induced using 25 μM Aβ_1-42_, resulting in 52 % reduction in cell viability after 24 h as shown in Fig. 6A. Co-incubation of the above apoptotic cells with 6.25, 12.5, and 25 μM **B14** increased cell viability by 17 %, 26 %, and 38 %, respectively in a dose-dependent manner. The FITC Annexin V/PI apoptosis experiment was carried out with result as shown in Fig. S7B. The apoptosis ratio after Aβ_1-42_ induction was determined to be 17.70 %, which was significantly higher than the control (1.79 %). Upon addition of **B14**, the apoptosis ratio was decreased in a dose-dependent manner. Depolarization of mitochondria during the early stages of apoptosis leads to a decrease in mitochondrial membrane potential, which is an indicator of early apoptosis. JC-1 fluorescent probe could monitor this process by changing the intensity of red/green fluorescence [62]. As illustrated in Fig. 6D, Aβ_1-42_ treatment caused mitochondrial depolarization and decreased the red/green fluorescence intensity ratio. After the addition of **B14**, the red/green fluorescence intensity ratio changed significantly in a dose-dependent manner, suggesting that **B14** could reduce the occurrence of early apoptosis. Using MitoTracker Crimson probes and H2DCFDA, we found that **B14** could attenuate oxidative stress without changing mitochondrial number in 25 μM Aβ_1-42_-induced apoptosis as shown in Fig. 6D. In addition, as shown in Fig. 6D, we observed by Fluo-5AM probing that **B14** could modulate aberrantly metabolized Ca^2+^ in model cells in a dose-dependent manner, thereby reducing apoptosis. BCL-2 mRNA level was reduced in the Aβ_1-42_ model, which was found to be increased upon **B14** treatment in a dose-dependent manner, as shown in Fig. 6B. In contrast, BAX mRNA level exhibited an opposite trend. Apoptotic pathways were further examined through Western blotting as shown in Fig. 6C and E. Our result showed decreased levels of pro-apoptotic proteins including BAX, Cleaved Caspase 3, Cleaved Caspase 9, Cyto-C, and Apaf-1 upon **B14** treatment in dose-dependent manners. These results indicated that upregulation of BCL-2 expression by **B14** could attenuate neuronal cell apoptosis through mitochondria-mediated endogenous apoptotic pathway.

### 3.7 Assessment of B14 on transporting blood-brain barrier (BBB)

**Fig. 7:**
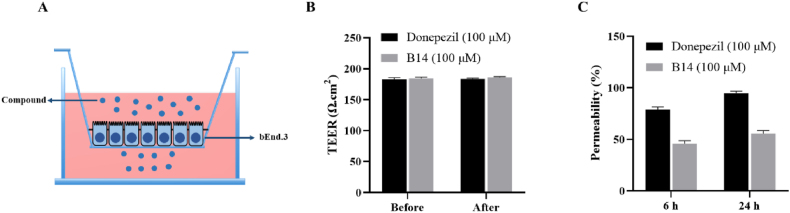
Measurement of *in vitro* blood-brain barrier (BBB) transport. (A) Schematic diagram of *in vitro* BBB model. (B) *Trans*-epithelial electrical resistance measurements (TEER) were performed to give corresponding values. (C) Permeability values were measured and calculated at 6h and 24h.

Previous studies have shown that the BBB plays a crucial role in the treatment of AD. Therefore, we utilized bEnd.3 cells to establish an *in vitro* BBB model, employing donepezil as a positive control to assess the BBB permeability of **B14**, as shown in Fig. 7A. *Trans*-epithelial electrical resistance measurements (TEER) were performed as shown in Fig. 7B, which showed values exceeding 200 Ω cm^2^ before and after incubation with **B14** or donepezil, indicating robust cellular integrity crucial for experimental reliability. **B14** exhibited permeabilities of 45.80 % at 6 h and 55.74 % at 24 h as shown in Fig. 7C, which were lower than the corresponding values of 78.80 % and 94.65 % for donepezil. Nevertheless, **B14** still showed reasonably good capability of transporting across the BBB.

### 3.8 B14 could significantly improve learning, memory and cognitive functions of APP/PS1 transgenic mice

**Fig. 8:**
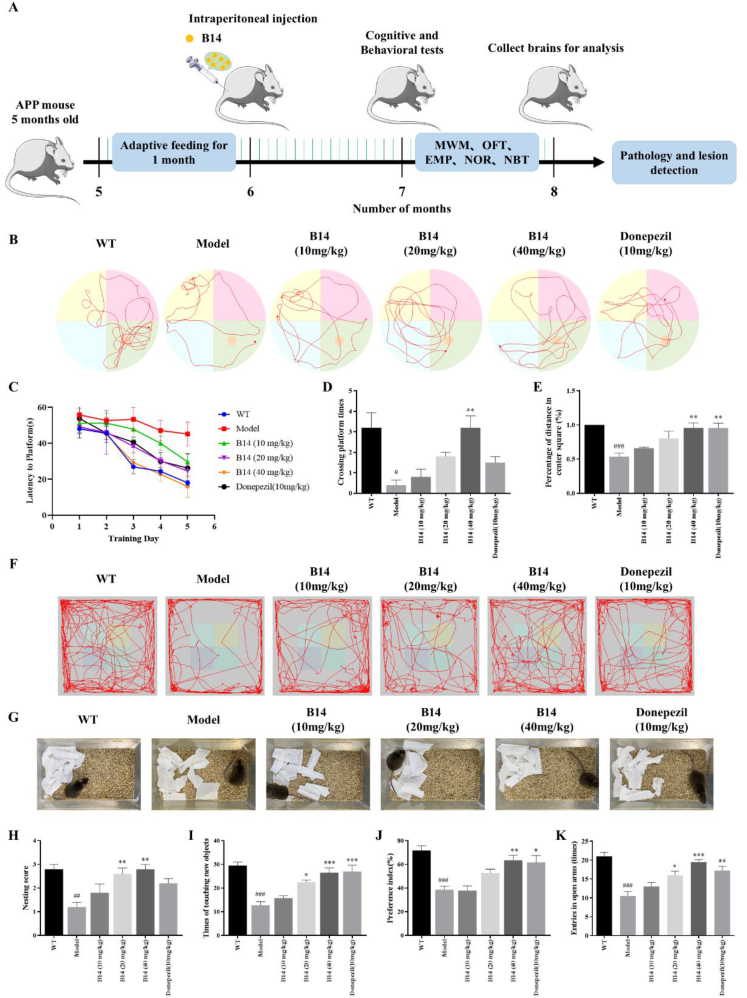
Effect of **B14** on behavioral cognitive deficits of APP/PS1 transgenic mice. (A) Animal experiment process and treatment plan. (B) Representative novel object recognition (MWM) trajectories for each group. (C) Escape latency during the first five days of the water maze experiment. (D) The times for the mice to cross the platform was counted on the sixth day of the MWM. (E) Percentage of mice in the center area active in the open field test (OFT). (F) Representative OFT trajectories for each group. (G) Representative nest building test (NBT) trajectories for each group. (H) Mice nesting experiment score. (I) The times for the mice to touch new objects in new object recognition experiments, with preference index as shown in (J). (K) Number of times for the mice to enter the open arm in EPM experiment. All the experiments were repeated for three times. The data were expressed as the mean ± SEM: ^#^p < 0.05, ^##^p < 0.01, ^###^p < 0.001 vs Control group; ∗p < 0.05, ∗∗p < 0.01, ∗∗∗p < 0.001 vs Model group, significantly different from the control.

To assess the effect of **B14** on learning, memory and cognitive ability of APP/PS1 transgenic mice, we conducted *in vivo* animal experiments. For this evaluation, we administered compound **B14** by intraperitoneal injection into a 6-month-old mice model for two months, with donepezil as a positive control. The behavioral experiments were performed one month after drug administration. MWM is a classical evaluation experiment to test the spatial memory ability. Our results showed that compared with the model group, the escape latency became significantly short after **B14** treatment as shown in Fig. 8C and Figs. S9A–F, while the number for the mice to cross the platform became significantly increased as shown in Fig. 8D both in dose-dependent manners. OFT is commonly used to detect and analyze spontaneous activity and exploratory ability of animals. Here we evaluated the activity of mice by counting their distance in the central area, followed by analyzing the movement trajectory (Fig. 8E and F) and other movement indexes (Figs. S9G–J). Our result showed a significant improvement in the range and duration of activity for the AD mice after **B14** treatment in a dose-dependent manner. NBT can reflect the social behavior and daily activity of mice. We found that the appearance and shape of the nesting material changed significantly after **B14** treatment. The paper pieces were gathered together to form a nest, and their nesting scores were significantly improved upon **B14** treatment in a dose-dependent manner as shown in Fig. 8G and H. NOR experiment is a behavioral method for mice to identify the precision of memory and sensitivity to external exploration. Our results showed that the times for the mice to touch new objects and the index of preference were significantly increased after **B14** treatment in dose-dependent manners as shown in Fig. 8I and J. The EPM experiment can assess the anxiety behavior of mice by comparing the retention time and distance traveled within the open and closed arms. As shown in Fig. 8K and Fig. S9L, the number of entries into the open arm and the retention time significantly increased following **B14** treatment, indicating that **B14** could effectively reduce anxiety of AD mice. In summary, our above results showed that compound **B14** could effectively improve the learning, memory and cognitive functions of APP/PS1 mice, enhance their autonomous activity, and alleviate their anxiety.

### 3.9 Protective effect of B14 on hippocampal neurons of APP/PS1 transgenic mice

**Fig. 9:**
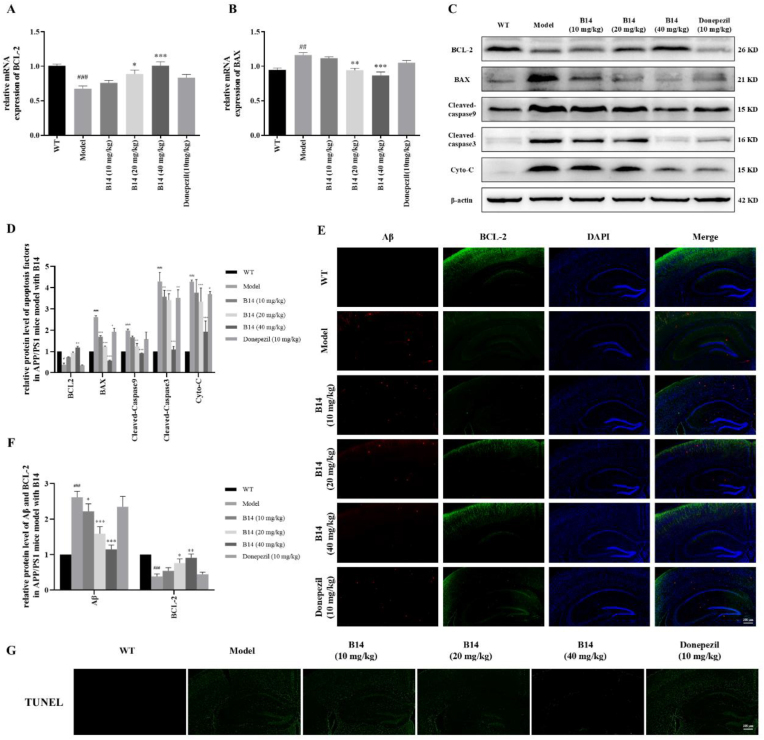
Effects of **B14** on Aβ and expressions of various apoptosis related factors in the brain of APP/PS1 mice. (A) Effect of **B14** on BCL-2 gene transcription. (B) Effect of **B14** on BAX gene transcription. (C) Effects of **B14** on expressions of various apoptosis related factors including BCL-2, BAX, Cleaved-caspase9, Cleaved-caspase3 and Cyto-C in the hippocampus of mice, with their statistical histograms as shown in (D). (E) Fluorescence images of brain tissues were collected from different treatment groups with immunohistochemical stains of Aβ and BCL-2, and their corresponding histograms were as shown in (F). The scale bar was 300 μm. (G) Fluorescence images of brain tissues were collected from different treatment groups with immunohistochemical stains of TUNEL. The scale bar was 300 μm. All the experiments were repeated for three times. The data were expressed as the mean ± SEM: ^#^*p* < 0.05, ^##^*p* < 0.01, ^###^*p* < 0.001 vs Control group; ∗*p* < 0.05, ∗∗*p* < 0.01, ∗∗∗*p* < 0.001 vs Model group, significantly different from the control.

In order to examine whether **B14** had anti-apoptotic and Aβ deposition inhibitory activities *in vivo*, we performed Western blot and immunofluorescence experiments, with the results as shown in Fig. 9. It was found that the transcriptional and translational levels of BCL-2 were decreased while those of BAX were increased in the hippocampus of mice model in comparison with those of wild type mice. Our results showed that **B14** treatment effectively increased the expression level of BCL-2 while decreased the expression levels of BAX, Cyto-C, Cleaved-caspase 9 and Cleaved-caspase 3 in dose-dependent manners. Consistent with the protein blotting results, immunofluorescence staining of brain sections also showed that the expression level of BCL-2 in the hippocampal region of AD mice was much lower than that of wild type mice, whereas **B14** treatment significantly increased BCL-2 expression in a dose-dependent manner. Then immunofluorescence experiment was performed to investigate the effect of **B14** on Aβ deposition. As shown in Fig. 9E and F, hippocampal Aβ deposition in AD mice was much increased in comparison with that in wild type mice, which was significantly reduced in a dose-dependent manner after **B14** administration. Next, TUNEL staining was used to determine the number of apoptotic neurons. As shown in Fig. 9G, the number of TUNEL-positive neurons was much increased in the model group, whereas **B14** treatment significantly decreased the proportion of TUNEL-positive neurons in a dose-dependent manner. Our above results showed that **B14** could effectively increase the expression level of BCL-2, alleviate the apoptosis of neuronal cells in the hippocampus of AD mice through the mitochondria-mediated endogenous apoptosis pathway, and inhibit the Aβ deposition in the hippocampal region, consequently exerting a protective effect.

### 3.10 Effect of B14 on neuronal morphology and synaptic ultrastructure in APP/PS1 transgenic mice

**Fig. 10:**
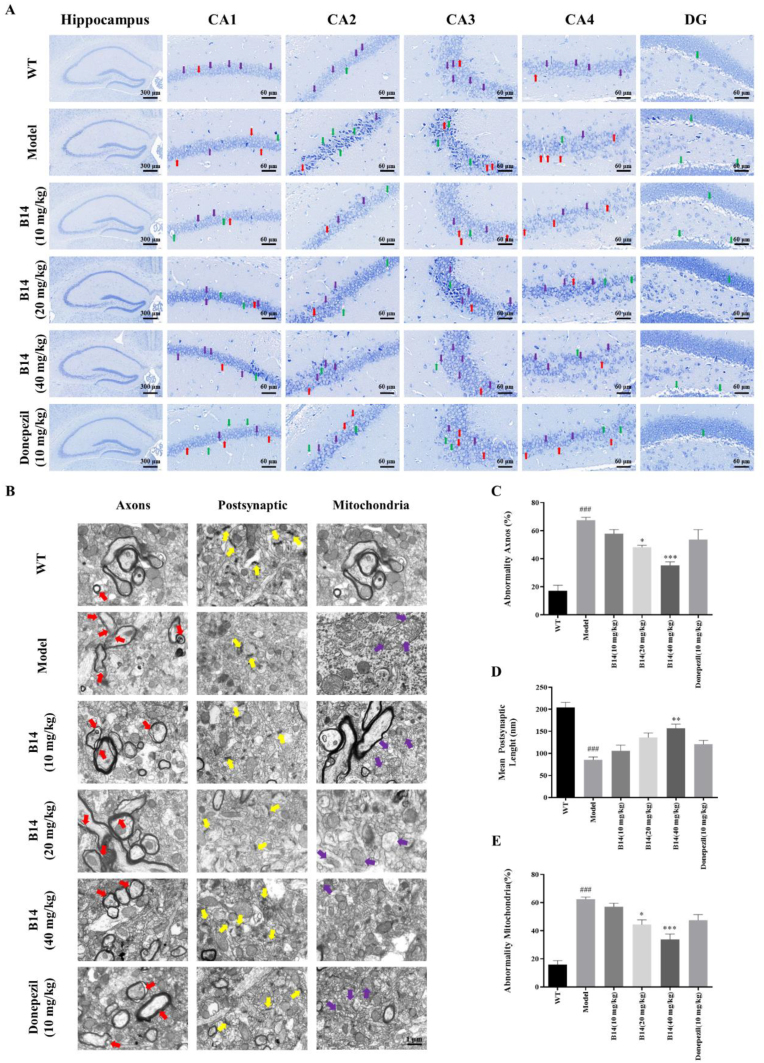
Effect of **B14** on hippocampal neurons of APP/PS1 transgenic mice. (A) Nissl staining was performed with Nissl bodies indicated by purple arrows, nuclear consolidation indicated by red arrows, and cellular edema indicated by green arrows. The scale bar was 300 μm or 60 μm. (B) The images were obtained through transmission electron microscopy for mice hippocampal neuron myelin, synapses, and mitochondria. The mitochondrial damage to myelin sheaths was indicated by red arrows. The post-synaptic dense bands became thin, as indicated by yellow arrows. The rupture of mitochondrial membranes and spine degeneration was indicated by purple arrows. The scale bar was 1 μm. (C–E) Statistical charts of indicators. All the experiments were repeated for three times. The data were expressed as the mean ± SEM: ^###^p < 0.001 vs Control group; ∗p < 0.05, ∗∗p < 0.01, ∗∗∗p < 0.001 vs Model group, significantly different from the control.

Nissl bodies serve as critical sites for protein synthesis in neurons, pivotal for nerve excitation and conduction. We assessed hippocampal damage in mice by Nissl staining, as shown in Fig. 10A. Compared with model group, the number of Nissl bodies distributed in the CA1-CA4 and DG regions of the mice hippocampus (purple arrows) was restored after **B14** treatment, while nucleus consolidation (red arrows) and cellular edema (green arrows) were markedly reduced, indicating that **B14** could preserve neuronal function. HE staining (Fig. S10) revealed a significant reduction in cell density and disorganized cell arrangement in the CA region of the hippocampus of mice in model group, as well as the presence of an obvious wide edema area in the DG region (red arrows) and significant pyknosis (blue arrows). After the treatment of **B14**, neuronal cells in the hippocampal region of mice were found to be arranged in a tight and orderly manner, with the number of cells increased significantly, pyknosis and cell edema decreased significantly, suggesting that **B14** could reverse the damage of hippocampal neurons in mice. We also observed and analyzed the ultrastructure of mice hippocampal neurons, the number of synapses and the mitochondrial structure by using transmission electron microscopy, with the results obtained as shown in Fig. 10B–E. For the mice in model group, the myelin sheaths were found to be loosened (red arrowheads), the postsynaptic dense bands (yellow arrowheads) became thin with the number reduced, and the mitochondria showed membrane rupture and ridge degeneration (purple arrowheads). Upon treatment with **B14**, the myelin sheaths were found to be tightly arranged, the postsynaptic dense bands became thick, and the degree of mitochondrial damage was much reduced in dose-dependent manners. Our above results showed that **B14** could significantly preserve neuronal morphology and function, safeguard hippocampal neurons, and enhance neuronal viability in AD mice.

### 3.11 B14 had beneficial effect on neuroinflammation and oxidative stress damage in the hippocampus of APP/PS1 transgenic mice without significant toxicity

**Fig. 11:**
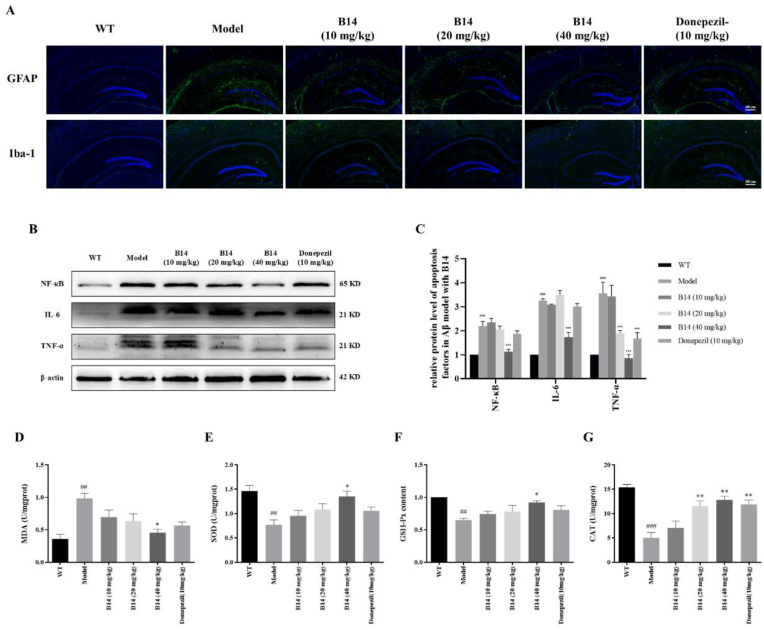
Effects of **B14** on neuroinflammation and oxidative stress damage in the hippocampus of APP/PS1 transgenic mice with its toxicity assessment. (A) Effects of **B14** on the expressions of astrocytes (glial fibrillary acidic protein, GFAP) and microglia (ionized calcium binding adaptor molecule 1, Iba-1). The scale bar was 200 μm. (B) Effects of **B14** on the expressions of inflammatory factors including NF-κB, TNF-α and IL-6 with their statistical histograms as shown in (C). (D–G) Effects of **B14** on the levels of malondialdehyde (MDA), superoxide dismutase (SOD), glutathione peroxidase (GSH-Px) and cathepsin (CAT). All the experiments were repeated for three times. The data were expressed as the mean ± SEM: ^##^*p* < 0.01, ^###^*p* < 0.001 vs Control group; ∗*p* < 0.05, ∗∗*p* < 0.01 vs Model group, significantly different from the control.

Neuronal apoptosis and increased Aβ deposition can activate glial cells, which in turn induce neuroinflammation. Therefore, we investigated whether **B14** had an anti-inflammatory effect. Our result showed that **B14** could reduce the activations of astrocytes (glial fibrillary acidic protein, GFAP) and microglia (ionized calcium binding adaptor molecule 1, Iba-1) in dose-dependent manners as shown in Fig. 11A. **B14** could also decrease the expressions of the inflammatory factors NF-κB, TNF-α and IL-6 in the hippocampus of APP mice as shown in Fig. 11B and C. Then we examined oxidative stress-related indicators in the mice hippocampus, with the results as shown in Fig. 11D–G. **B14** could reduce the levels of lipid peroxides MDA and restore the levels of antioxidant enzymes (SOD, GSH-Px, and CAT) in dose-dependent manners, thereby alleviating the oxidative stress damage in the hippocampus of AD mice. In addition, we performed histopathological examinations of the organs including heart, liver, spleen, lungs, and kidney under various experimental conditions as shown in Fig. S11, and our results showed that **B14** had no significant detrimental effect for these organs. In summary, our above results demonstrated that **B14** could effectively attenuate neuronal apoptosis and Aβ deposition in the hippocampus of AD mice, protect neuronal morphology and function, mitigate pathological changes including neuroinflammation and oxidative stress injury, without significant side effect. Therefore, **B14** could become a promising bi-target directed lead compound with multiple beneficial effect for further development for the treatment of Alzheimer's disease.

## 4 Discussion

Currently, clinical therapeutic agents for AD mainly include some AchE inhibitors, non-competitive NMDA receptor antagonists and antioxidants. However, these drugs target single pathways, and are ineffective in reversing pathological changes like amyloid plaque deposition and neurofibrillary tangles in the AD brain. These drugs usually have side effects and fail to halt the rapid progression of AD. Therefore, active research of AD pathogenesis for new and effective treatment is one of the most urgent issues facing geriatrics. Many studies have highlighted a significant link between AD and the expression of anti-apoptotic protein BCL-2. Decreased BCL-2 expression could trigger neuronal apoptosis, a hallmark of AD pathology. Conversely, increasing BCL-2 expression shows promise in ameliorating AD symptoms. Therefore, inhibition of neuronal apoptosis by up-regulating BCL-2 expression is expected to be a new option for AD treatment. In recent years, research on the role of apoptosis regulation in the pathogenesis of AD has become a major hot-spot, with its specific mechanism involving the complex regulation of a series of genes. A variety of factors can cause neuronal apoptosis, including Aβ, calcium overload, oxidative stress, and insufficient nerve growth factor secretion. Among these factors, Aβ-induced neuronal apoptosis can selectively down-regulate the expression of the BCL-2 family of apoptosis-related genes, and the anti-apoptotic factor BCL-2 has been found to be reduced in degenerating neurons of post mortem AD brains, which makes BCL-2 becoming a new potential target for anti-AD therapy.

As we discussed above, for AD with complex pathogenesis and diverse pathologic manifestations, single-target drug treatment strategies exert limited effects. In turn, new evidence emphasizes the existence of a triad of interactions among amyloidogenesis, dysregulation of apoptosis, and oxidative stress, which can synergistically drive neurodegeneration, thereby accelerating the progression of the AD disease course [63]. Therefore, the design and development of innovative multi-targeting anti-AD drugs could offer new solution for the treatment of AD. We have previously revealed that an acridone derivative could selectively bind to oncogene promoter i-motif and regulate gene transcription [37]. We have also found that acridone derivative **A22** could alleviate liver damage of NAFLD/NASH by upregulating the expression of BCL-2 through stabilization of *BCL-2* promoter i-motif [47]. We applied this finding to AD model constructed and induced by excessive neuronal apoptosis, and found that **A22** showed a similar therapeutic effect and mild inhibition of Aβ_1-42_ deposition on this model. Therefore, we optimized the structure through modification of **A22**, in order to enhance its inhibition of Aβ_1-42_. We introduced a benzofuran moiety (for inhibition of Aβ aggregation) into the acridone skeleton (for i-motif binding), and synthesized a series of acridone-benzofuran derivatives through a multi-target directed ligand strategy. After extensive screening, we found that **B14** could bind to and stabilize *BCL-2* promoter i-motif without significant interaction with its corresponding G-quadruplex, which could also inhibit Aβ aggregation effectively. Our molecular modeling study indicated that the acridone ring of **B14** interacted with the i-motif loop structure through hydrogen bonding interaction, with its side chain forming hydrogen bonds to the phosphodiester of the i-motif. The addition of a benzofuran moiety appeared to have no detrimental effect on this interaction. In contrast, G-quadruplex binding ligands typically require extended planar systems with large fusion rings such as porphyrins to interact with flat G-quartets on the end of G-quadruplex structure through π-π stacking. The introduction of a benzofuran moiety appeared to have no significant beneficial effect on this interaction possibly due to its unprecise positioning. Our subsequent experiments demonstrated **B14** had excellent selectivity for binding and stabilization to *BCL-2* promoter i-motif for up-regulating gene expression. In Aβ_1-42_-induced cellular model, **B14** could disrupt the endogenous apoptotic cascade initiated by Bax activation and alter mitochondrial membrane permeability by simultaneously upregulating BCL-2 expression and inhibiting the intracellular deposition of Aβ aggregates. This mechanism parallels the efficacy of Allopurinol in attenuating COVID-19-related endothelial oxidative dysfunction [64]. The structural hybrid of acridone (i-motif binding) and benzofuran (Aβ interaction) in **B14** exemplifies rational polypharmacology. This multimodal effect is similar to the synergistic protective effect evidenced by the combination of single-target drugs, such as the hexoketone-cocobalamin/berberine combination [65].

Similar to the reported TGR63 [66], **B14** showed good capability of transporting across the BBB. Subsequent animal experiments demonstrated that **B14** could significantly inhibit the deposition of Aβ plaques in the hippocampus or cortex of mice, upregulate the expression of BCL-2 and alleviate oxidative stress damage in the brains of AD mice. HE section staining and electron microscopic ultrastructural analysis showed that **B14** could alleviate the abnormal changes in the ultrastructural synapses of hippocampal neurons in mice and reduce cell edema and shrinkage, without significant toxicity. The behavioral experiments showed that **B14** could effectively improve the learning, memory and cognitive functions of AD mice. Oxidative stress, driven by excess ROS, damages lipids, proteins, and DNA, leading to neuronal apoptosis and inflammation, a key factor in neurodegenerative diseases. Notably, **B14** showed ability to reduce MDA levels while restoring SOD activity, which suggested engagement with the Nrf2/Keap1 antioxidant axis, a critical regulator of vitagenes like heme oxygenase-1 (HO-1) [67]. This parallels recent findings that phytochemicals (e.g., Panax ginseng saponins) ameliorate oxidative organ toxicity by balancing Nrf2-mediated defenses and inflammatory/apoptotic cascades [68]. This dual targeting of apoptosis and oxidative stress resonates with Calabrese et al.’s vitagene hypothesis, wherein coordinated upregulation of cytoprotective genes (e.g., HO-1, NQO1) confers resilience against neurodegenerative insults [69]. Notably, Calabrese et al. emphasized that coordinated upregulation of these stress-responsive genes is critical for maintaining redox homeostasis in neurodegenerative contexts, which may be related to the core mechanism by which **B14** exerts its antioxidant effects and deserves further exploration.

Although the present study confirms that **B14** is effective in alleviating AD pathology through dual targeting *BCL-2* i-motif and Aβ deposition, its potential multisystemic role remains to be explored in depth. First, recent studies have found that abnormal platelet APP processing may exacerbate AD progression through peripheral Aβ production [70], and future studies are needed to assess whether **B14** regulates APP shearase activity in platelets to block this process. Second, given the strong association between Aβ deposition and cardiovascular dysfunction [71], it will be crucial to explore the protective effects of **B14** on cardiac mitochondrial homeostasis and vascular endothelial function. Finally, the emerging role of imbalanced uric acid metabolism in AD oxidative stress [66] suggests that **B14** may further enhance its antioxidant efficacy by interfering with xanthine oxidase activity or uric acid transport homeostasis. These research directions will not only improve the mechanism for action mapping of **B14**, but also provide theoretical support for the development of 10.13039/100020014AD therapeutic strategies with both central and peripheral efficacy.

In conclusion, **B14** represents a paradigm shift in AD therapeutics by concurrently targeting amyloid pathology, apoptotic signaling, and oxidative stress. Its efficacy in restoring BCL-2 expression and mitigating Aβ toxicity, coupled with favorable BBB permeability, positions it as a promising candidate for further development for clinical application. This work also pioneers the exploitation of i-motif structures as epigenetic regulators in neurodegeneration, offering a roadmap for next generation multi-target drug development.

## CRediT authorship contribution statement

**Dongsheng Ji:** Writing – original draft, Investigation, Conceptualization. **Jiahui Zhang:** Investigation. **Jihai Liang:** Investigation. **Zhi-Shu Huang:** Project administration. **Bing Shu:** Investigation. **Ding Li:** Writing – review & editing, Supervision, Funding acquisition, Conceptualization.

## Funding

We thank 10.13039/501100001809National Natural Science Foundation of China (Grant 22477147, 22007018), 10.13039/501100021171Guangdong Basic and Applied Basic Research Foundation (Grant 2024A1515012234, 2021A1515011530), and Guangdong Provincial Education Department Fund (Grant 2023ZDZX2028) for financial support of this study.

## Declaration of competing interest

The authors declare that they have no known competing financial interests or personal relationships that could have appeared to influence the work reported in this paper.

## Data Availability

Supplementary Data are available at Redox Biology Online.
