# Supplementary material for: Efficient strategy for alleviating neuronal apoptosis and oxidative stress damage of Alzheimer's disease through dual targeting BCL-2 gene promoter i-motif and β-amyloid
Source: Redox Biol. 2025 Mar 18;82:103600. doi: 10.1016/j.redox.2025.103600 (PMC11982498; doi:10.1016/j.redox.2025.103600)
Supplement: Multimedia component 1 [file mmc1.docx]

**Supplementary Material for**

**Efficient strategy for alleviating neuronal apoptosis and oxidative stress damage of Alzheimer's disease through dual targeting BCL-2 gene promoter i-motif and β-amyloid**

Dongsheng Ji^a^, Jiahui Zhang^a^, Jihai Liang^a^, Zhi-Shu Huang^a^, Bing Shu^b^, and Ding Li^a,^*

^a^ School of Pharmaceutical Sciences, Sun Yat-sen University, Guangzhou University

City, 132 Waihuan East Road, Guangzhou 510006, P. R. China

^b^ School of Pharmacy, Guangdong Pharmaceutical University, Guangzhou, P. R. China

* Corresponding author:

Ding Li, Tel: 8620 3994 3058; E-mail: [liding@mail.sysu.edu.cn](mailto:liding@mail.sysu.edu.cn)

**Table of Contents**

| **1. Materials** | S4 |
| --- | --- |
| **Table S1.** The oligonucleotides used in the present study | S4 |
| **Figure S1.** Marketed drugs for the treatment of AD and design strategy for acridone-benzofuran-piperidine derivatives | S5 |
| **2. Experimental Section** | S6 |
| **Scheme S1.** Synthetic pathway for novel acridone-benzofuran derivatives **A1-A16** and **B1-B14** | S7 |
| **3. Other Supporting Table, Spectra and Graphs** | S20 |
| **Table S2.** Effect of compounds on Aβ_1-42_ aggregation | S20 |
| **Table S3.** Equilibrium binding constants (*K*_D_) determined by using SPR | S21 |
| **Table S4.** Equilibrium binding constants (*K*_D_) determined by using SPR | S22 |
| **Table S5.** Relative TO displacement ratio (%) of selected compounds after screening through SPR experiment | S22 |
| **Table S6**. IC_50_ values for effect of **B14** on various cell lines determined by using MTT after incubation for 48 h | S23 |
| **Table S7.** Primers sequence information | S23 |
| **Table S8.** The qPCR raw data from the real-time RT-PCR assay for cells and animal samples | S23 |
| **Figure S2.** The fluorescence intensity of different concentrations of **B14** was measured after incubation with Aβ_1-42_ | S25 |
| **Figure S3.** Biophysical experiments were performed for binding of acridine derivative to *BCL-2* promoter i-motif and other DNA structures | S26 |
| **Figure S4.** TO displacement and UV titration experiments were performed to examine the effects of **B14** and **A22** on *BCL-2* promoter i-motif | S27 |
| **Figure S5**. ESI-MS, UV, CD, and EMSA experiments were performed to examine the effect of **B14** on *BCL-2* promoter i-motif and G-quadruplex | S28 |
| **Figure S6.** CD melting experiments were performed to examine the effects of **B14** and **A22** on *BCL-2* promoter i-motif and G-quadruplex  **Figure S7.** Effect of **B14** on SH-SY5Y cells incubated with Aβ_1-42_  **Figure S8.** Effect of **B14** on the body weights of APP/PS1 transgenic mice in comparison with that of donepezil  **Figure S9.** Effect of **B14** on behavioral cognitive deficits in APP/PS1 transgenic mice  **Figure S10.** Histopathological staining showed hippocampal morphology in APP/PS 1 transgenic mice | S29  S30  S30  S31  S32 |
| **Figure S11.** HE staining was performed for vital organs including heart, liver, spleen, lung, and kidney under various experimental conditions | S32 |
| **4. MATERIAL AND METHODS** | S33 |
| **5. NMR spectra and HPLC profiles of compounds** | S40 |

**1. Materials**

**Table S1.** The oligonucleotides used in the present study

| Name | Sequence (5’-3’) |
| --- | --- |
| *BCL-2* | CAGCCCCGCTCCCGCCCCCTTCCTCCCGCGCCCGCCCCT |
| *BCL-2(G4)* | AGGGGCGGGCGCGGGAGGAAGGGGGCGGGAGCGGGGCTG |
| bio-*BCL-2* | biotin-d[CAGCCCCGCTCCCGCCCCCTTCCTCCCGCGCCCGCCCCT] |
| bio- *BCL-2*(G4) | biotin-d [AGGGGCGGGCGCGGGAGGAAGGGGGCGGGAGCGGGGCTG] |
| bio-hairpin | biotin-d[TATAGCTATA-HEG-TATAGCTATA] |
| bio-*HRAS* | biotin-d[CGCCCGTGCCCTGCGCCCGCAACCCGA] |
| bio-*ILPR* | biotin-d[TGTCCCCACACCCCTGTCCCCACACCCCTGT] |
| bio-*(G_2_C_4_)_6_* | biotin-d[GGCCCCGGCCCCGGCCCCGGCCCCGGCCCCGGCCCC] |
| F-*BCL-2*-T | FAM-CAGCCCCGCTCCCGCCCCCTTCCTCCCGCGCCCGCCCCT-TAMRA |
| F-*BCL-2*(G4)-T | FAM-AGGGGCGGGCGCGGGAGGAAGGGGGCGGGAGCGGGGCTG-TAMRA |
| F-*BCL-2* | FAM-CAGCCCCGCTCCCGCCCCCTTCCTCCCGCGCCCGCCCCT |
| F-*BCL-2(*G4) | FAM-AGGGGCGGGCGCGGGAGGAAGGGGGCGGGAGCGGGGCTG |
| F-*TEL-C*-T | FAM-CCCTAACCCTAACCCTAACCCTAA-TAMRA |
| F-*C-KIT*-T | FAM-CCCTCCTCCCAGCGCCCTCCCT-TAMRA |
| F-*C-MYC*-T | FAM-CCCCACCTTCCCCACCCTCCCCACCCTCCCC-TAMRA |
| F-*C-JUN*-T | FAM-TAACCCCCTCCCCCTCCCCCCTTTAAT -TAMRA |
| hairpin | CGCGCGCGTTTTCGCGCGCG |
| *RET* | CGCGCGCGTTTTCGCGCGCG |
| *TEL-C* | CCCTAACCCTAACCCTAACCCTAA |
| *C-KIT* | CCCTCCTCCCAGCGCCCTCCCT |
| *HRAS* | CGCCCGTGCCCTGCGCCCGCAACCCGA |
| *VEGF* | GACCCCGCCCCCGGCCCGCCCCGG |
| *RB* | GCCGCCCAAAACCCCCCG |
| *C-JUN* | TAACCCCCTCCCCCTCCCCCCTTTAAT |
| *KRAS* | GCCCGGCCCCCGCTCCTCCCCCGCCGGCCCGGCCCGGCCCCCTCCTTCTCCCCG |


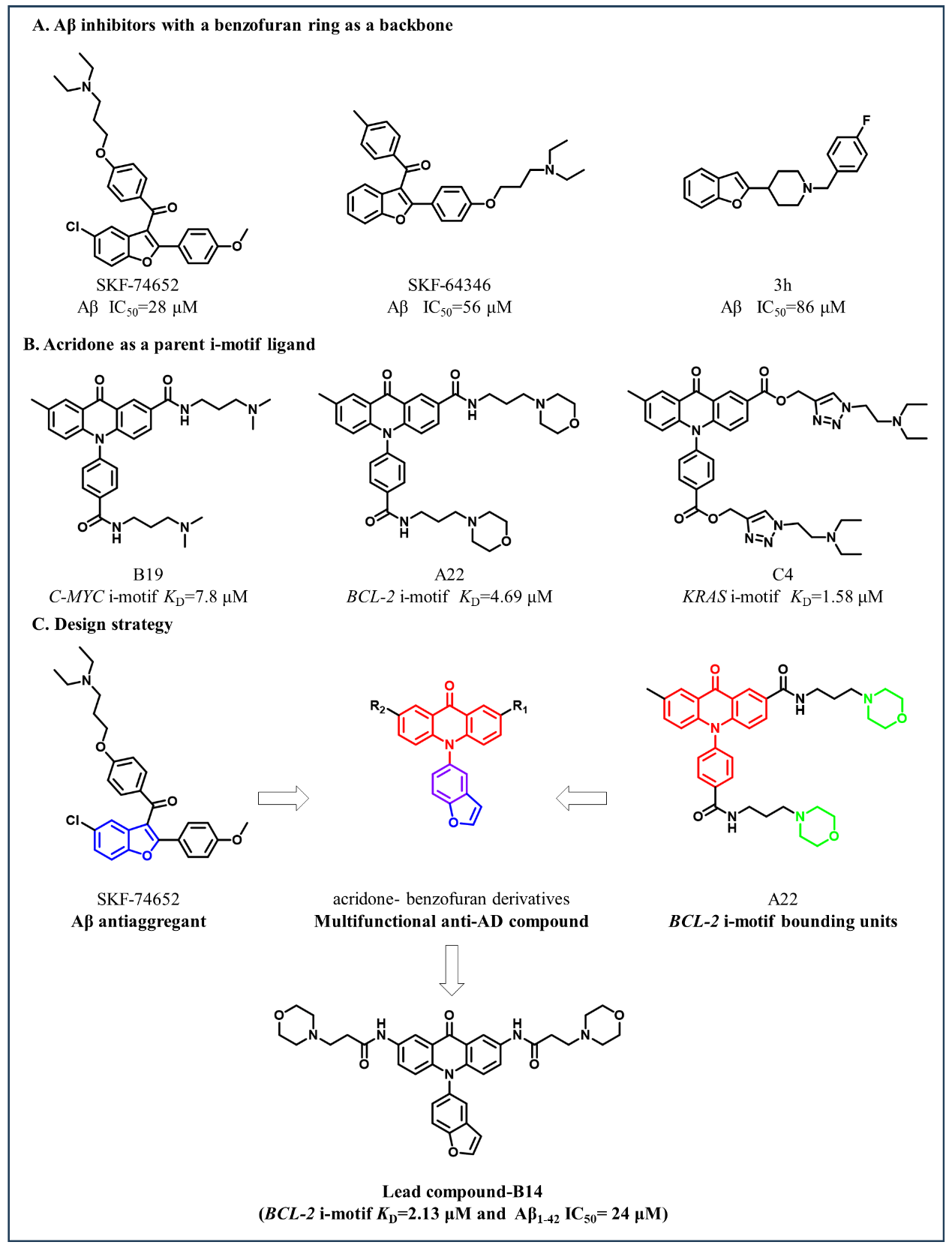


**Figure S1.** Lead compounds for the treatment of AD and design strategy for acridone-benzofuran-piperidine derivatives.

**2 Experimental Section**

**2.1. Synthesis and Characterization**

All chemicals were purchased from commercial sources, which were analytical grade without further purification unless otherwise specified. Synthesized compounds were characterized by using ^1^H and ^13^C NMR, HSQC, HMBC, and HRMS spectrometry. ^1^H and ^13^C NMR spectra were recorded using TMS as the internal standard in DMSO-d_6_ or CDCl_3_ with a Bruker BioSpin GmbH spectrometer. High resolution mass spectra (HRMS) were recorded on Shimadzu LCMS-IT-TOF of MAT95XP mass spectrometer (Thermo Fisher Scientific, American). The purity of the synthesized compounds was confirmed to be higher than 95% by using analytical HPLC performed with a dual pump Shimadzu LC-20 AB system equipped with an Ultimate XB-C18 column (4.6 mm × 250 mm, 5 μm), eluting with methanol-water (47:53) containing 0.05% trifluoroacetic acid.

The synthetic pathway for novel acridone-benzofuran derivatives **A1-A16** and **B1-B14** were as shown in **Scheme 1**. Acridone-benzofuran **2** was obtained through Ullman reaction of acridin-9(10H)-one (**1**) with 5-bromobenzofuran, followed by reaction with bromine in acetic acid at 120 °C for 8 h to give brominated acridone-benzofuran **3a** and **3b**. Then, **3a** and **3b** reacted with compounds containing various amino/amide groups to introduce different amino or amide side chains via Pd-catalyzed coupling to give the final products **A1-A16** and **B1-B14**, respectively. The synthesized compounds **A1-A16** and **B1-B14** were purified and their structures were characterized by using ^1^H-NMR, ^13^C-NMR, HSQC, HMBC, and HRMS as detailed in supporting information. All compounds used for subsequent biological and biochemical evaluations as well as cell-based studies were analyzed by using HPLC with their purity determined to be more than 95%.


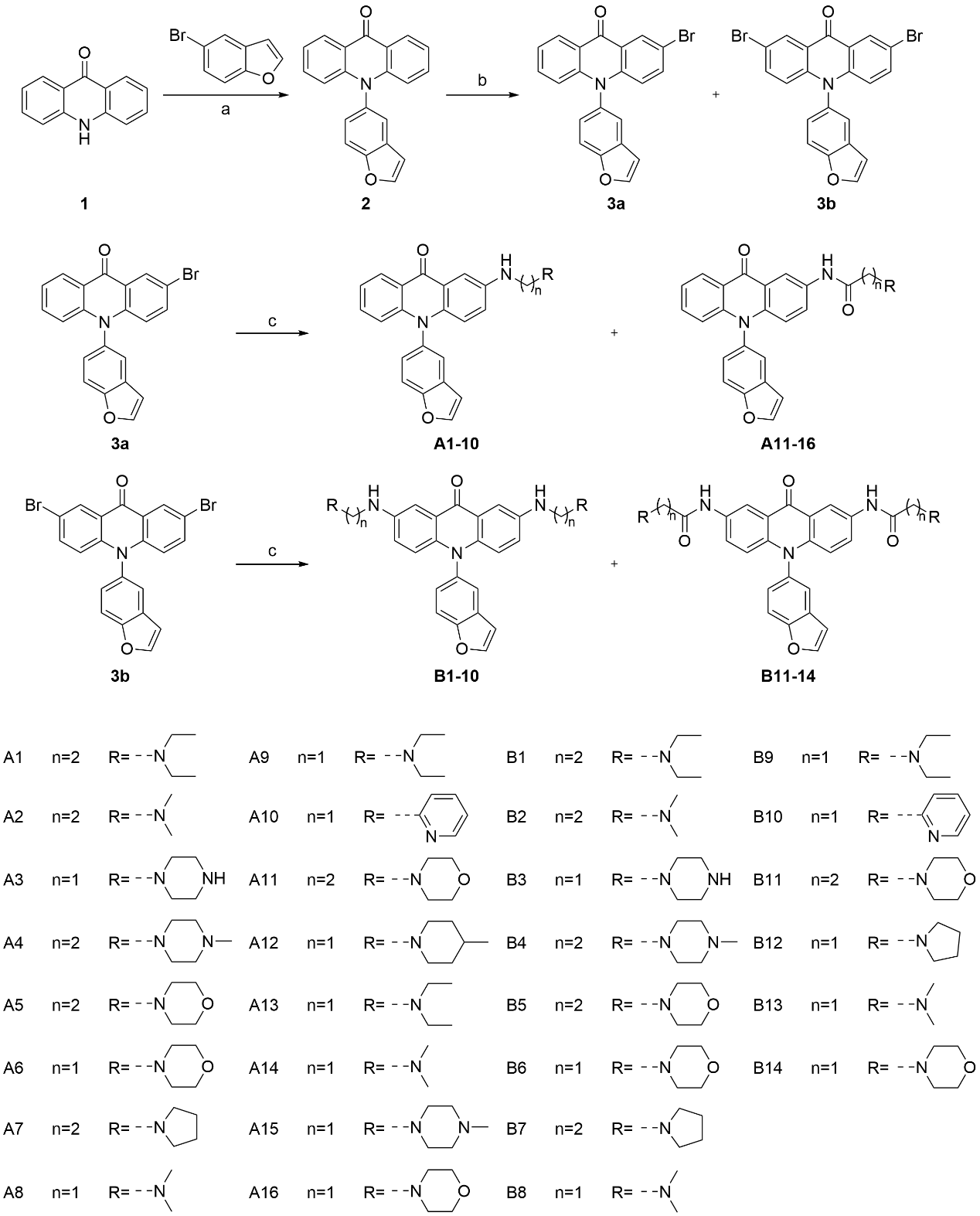


**Scheme S1.** Synthetic pathway for novel acridone-benzofuran derivatives **A1-A16** and **B1-B14.** Reagents and conditions: (a) CuI, K_2_CO_3_, 2,2,6,6-tetramethyl-3,5-heptanedione, DMF, 140 °C, 20 h, 55%; (b) Br_2_, CH_3_COOH, 120 °C, 8 h (yields for **3a** and **3b** were 67% and 54%); (c) compounds with various amino/amide groups, Pd(OAc)_2_, t-BuOK, Xantphos, 1,4-dioxane, 100 °C, 12 h (56-72% yield).

**2.1.1. General procedure for preparation of A1-A16**

*Synthesis of 10-(benzofuran-5-yl)acridin-9(10H)-one (****2****).* To a solution of acridin-9(10H)-one (3.12 g 16.4 mol) in 50 mL anhydrous dimethylformamide (50 mL), was added 5’-bromobenzofuran (2.7 g, 13.7 mol), K_2_CO_3_ (2.8 g, 20.51 mol), CuI (0.522 g, 2.74 mol) and 2,2,6,6-tetramethyl-3,5-heptanedione (1.08 g, 5.4 mol). The mixture was heated under reflux with stirring in a nitrogen atmosphere for 20 h. After being cooled down, the mixture was poured into ice water and stirred. Ethyl acetate (50 mL) was added and stirred for 10 min and filtered, and the residue was extracted with ethyl acetate (2 × 20 mL). The combined ethyl acetate layer was washed with brine three times, dried over anhydrous sodium sulfate, filtered, concentrated and purified using chromatography on silica gel with petroleum ether/ethyl acetate (10/1-2/1) to give the desire intermediate **2**, 10-(benzofuran-5-yl)acridin-9(10H)-one (**2**), as a yellow solid with yield of 30%. ^1^H NMR (400 MHz, DMSO) δ 8.39 (d, J = 7.1 Hz, 2H), 8.23 (d, J = 2.0 Hz, 1H), 7.99 (d, J = 8.6 Hz, 1H), 7.88 (d, J = 1.7 Hz, 1H), 7.65 – 7.55 (m, 2H), 7.44 (dd, J = 8.6, 1.9 Hz, 1H), 7.33 (t, J = 7.4 Hz, 2H), 7.13 (d, J = 1.2 Hz, 1H), 6.76 (d, J = 8.6 Hz, 2H). ^13^C NMR (126 MHz, DMSO) δ 177.22, 154.63, 148.47, 143.70, 134.20, 133.85, 129.98, 126.77, 126.16, 123.39, 122.00, 121.53, 117.67, 114.46, 107.76. HRMS (EI): m/z [M+Na]^+^ calcd. for C_21_H_13_NO_2_: 334.0838. Found: 334.0851.

*Synthesis of 10-(benzofuran-5-yl)-2-bromoacridin-9(10H)-one (****3a****) and 10-(benzofuran-5-yl)-2,7-dibromoacridin-9(10H)-one (****3b****).* To a solution of intermediate **2** (1.15 g, 3.7 mol) in 30 mL CH_3_COOH, was added Br_2_ (1.5 g, 9.25 mol, dissolved in 10 mL CH_3_COOH). The mixture was heated under reflux for 8 h. After being cooled down, the mixture was poured into ice water and stirred. Ethyl acetate (30 mL) was added, and the mixture was stirred for 10 min and filtered, and the residue was extracted with ethyl acetate (2 × 10 mL). The combined ethyl acetate layer was washed with saturated sodium bicarbonate solution and brine for three times, dried over anhydrous sodium sulfate, filtered, concentrated and purified using chromatography on silica gel with petroleum ether/ethyl acetate (10/1-2/1) to give the desire intermediate **3a** and **3b**.

*10-(Benzofuran-5-yl)-2-bromoacridin-9(10H)-one* *(****3a****)*: A light yellow solid was obtained with a yield of 67%. ^1^H NMR (400 MHz, DMSO) δ 8.40 (d, J = 2.5 Hz, 1H), 8.34 (dd, J = 8.0, 1.7 Hz, 1H), 8.21 (d, J = 2.2 Hz, 1H), 7.97 (d, J = 8.6 Hz, 1H), 7.87 (d, J = 2.2 Hz, 1H), 7.72 (dd, J = 9.2, 2.6 Hz, 1H), 7.61 (ddd, J = 8.7, 6.9, 1.7 Hz, 1H), 7.43 (dd, J = 8.6, 2.2 Hz, 1H), 7.33 (t, J = 7.5 Hz, 1H), 7.11 (d, J = 2.3 Hz, 1H), 6.72 (dd, J = 15.0, 8.9 Hz, 2H). ^13^C NMR (101 MHz, DMSO) δ 175.07, 154.80, 148.66, 142.60, 137.02, 133.24, 130.04, 128.53, 125.83, 123.19, 122.72, 120.62, 114.91, 114.63, 107.80. HRMS (EI): m/z [M+H]^+^ calcd. for C_21_H_12_BrNO_2_: 390.0124. Found: 390.0132.

*10-(Benzofuran-5-yl)-2,7-dibromoacridin-9(10H)-one (****3b****):* A yellow solid was obtained with a yield of 54%. ^1^H NMR (400 MHz, DMSO) δ 8.33 (d, J = 2.3 Hz, 2H), 8.25 (d, J = 2.0 Hz, 1H), 8.01 (d, J = 8.6 Hz, 1H), 7.93 (d, J = 1.8 Hz, 1H), 7.75 (dd, J = 9.2, 2.4 Hz, 2H), 7.48 (dd, J = 8.6, 2.0 Hz, 1H), 7.15 (d, J = 1.5 Hz, 1H), 6.72 (d, J = 9.1 Hz, 2H). ^13^C NMR (126 MHz, DMSO) δ 175.00, 154.79, 148.66, 142.55, 136.98, 133.24, 130.03, 128.49, 125.83, 123.18, 122.65, 120.57, 114.90, 114.63, 107.80. HRMS (EI): m/z [M+H]^+^ calcd. for C_21_H_11_Br_2_NO_2_: 467.9229. Found: 467.9230.

*General procedure for preparation of* ***A1-A16****.* The bromine-substituted compound **3a** (100 mg, 0.256 mmol), t-BuOK (86 mg, 0.768 mmol), Xantphos (89 mg, 0.154 mmol), and Pd(OA_C_)_2_ (11.5 mg, 0.0512 mmol) were dissolved in 1,4-dioxane (10 mL). Then compound with different amino/amide side chain (3.0 equiv) was added and the reaction mixture was heat to 100 °C for 10 h under a nitrogen atmosphere. After being cooled to room temperature, 10 mL of ice water was added. dichloromethane (10 mL) was added, and the mixture was filtered. The filtrate was extracted with dichloromethane (10 mL) for two times. Then combined organic layers were washed with brine for three times, dried over anhydrous sodium sulfate, filtered, concentrated and purified using chromatography on silica gel with methanol/dichloromethane (20/1-5/1, 0.5% NH_3_.H_2_O) to give the desire intermediate **A1-A16**.

*10-(Benzofuran-5-yl)-2-((3-(diethylamino) propyl) amino) acridin-9(10H)-one (****A1****)*: A yellow solid was obtained with a yield of 33%. ^1^H NMR (400 MHz, CDCl_3_) δ 8.59 (dd, J = 8.1, 1.3 Hz, 1H), 7.80 (dd, J = 11.1, 5.4 Hz, 2H), 7.62 (dd, J = 15.7, 2.3 Hz, 2H), 7.45 – 7.39 (m, 1H), 7.25 – 7.16 (m, 2H), 6.94 – 6.87 (m, 2H), 6.74 (d, J = 8.6 Hz, 1H), 6.64 (d, J = 9.1 Hz, 1H), 3.35 (t, J = 6.1 Hz, 2H), 2.75 (dt, J = 13.8, 6.6 Hz, 6H), 2.07 – 1.82 (m, 2H), 1.15 (t, J = 7.1 Hz, 6H). ^13^C NMR (101 MHz, CDCl_3_) δ 177.75, 154.66, 146.90, 143.66, 142.86, 136.68, 134.16, 132.53, 129.51, 127.18, 125.87, 122.92, 122.79, 120.89, 120.68, 118.34, 116.75, 113.73, 107.01, 104.77, 51.45, 46.77, 43.25, 25.12, 10.75. HRMS (EI): m/z [M+H]^+^ calcd. for C_28_H_25_N_3_O_4_: 440.2333. Found: 440.2333.

*10-(Benzofuran-5-yl)-2-((3-(dimethylamino)propyl)amino)acridin-9(10H)-one (****A2****)* : A yellow solid was obtained with a yield of 43%. ^1^H NMR (400 MHz, CDCl_3_) δ 8.50 (dd, J = 8.1, 1.2 Hz, 1H), 7.72 (d, J = 2.1 Hz, 1H), 7.69 (d, J = 8.6 Hz, 1H), 7.60 (d, J = 2.8 Hz, 1H), 7.51 (d, J = 1.9 Hz, 1H), 7.36 – 7.30 (m, 1H), 7.17 – 7.10 (m, 2H), 6.83 – 6.75 (m, 2H), 6.65 (d, J = 8.6 Hz, 1H), 6.55 (d, J = 9.1 Hz, 1H), 3.22 (t, J = 6.6 Hz, 2H), 2.36 (t, J = 6.8 Hz, 2H), 2.18 (s, 6H), 1.81 – 1.71 (m, 2H). ^13^C NMR (101 MHz, CDCl_3_) δ 177.76, 154.64, 146.89, 143.72, 142.88, 136.65, 134.19, 132.54, 129.51, 127.21, 125.89, 122.93, 122.39, 120.89, 120.67, 118.25, 116.71, 113.73, 107.02, 105.44, 58.18, 45.49, 43.50, 26.70. HRMS (EI): m/z [M+H]^+^ calcd. for C_26_H_25_N_3_O_2_: 412.2020. Found: 412.2020.

*10-(Benzofuran-5-yl)-2-((2-(piperazin-1-yl)ethyl)amino)acridin-9(10H)-one (****A3****)*: A yellow solid was obtained with a yield of 48%. ^1^H NMR (400 MHz, CDCl_3_) δ 8.61 (dd, J = 8.1, 1.4 Hz, 1H), 7.82 (dd, J = 10.6, 5.3 Hz, 2H), 7.71 (d, J = 2.8 Hz, 1H), 7.62 (d, J = 1.8 Hz, 1H), 7.47 – 7.42 (m, 1H), 7.28 – 7.21 (m, 3H), 6.95 (dd, J = 9.1, 2.8 Hz, 1H), 6.91 (d, J = 1.2 Hz, 1H), 6.78 – 6.74 (m, 1H), 6.67 (dd, J = 9.2, 2.3 Hz, 1H), 4.43 (s, 1H), 3.32 – 3.27 (m, 2H), 2.97 (d, J = 4.3 Hz, 2H), 2.77 – 2.57 (m, 4H), 2.26 (s, 4H). ^13^C NMR (101 MHz, CDCl_3_) δ 177.71, 154.64, 146.91, 143.59, 142.89, 136.79, 134.14, 132.57, 129.52, 127.18, 125.82, 122.90, 122.87, 122.48, 120.89, 120.70, 118.24, 116.70, 113.74, 106.96, 105.62, 57.07, 53.49, 45.65, 40.54. HRMS (EI): m/z [M+H]^+^ calcd. for C_27_H_26_N_4_O_2_: 439.2129. Found: 439.2130.

*10-(Benzofuran-5-yl)-2-((3-(4-methylpiperazin-1-yl)propyl)amino)acridin-9(10H)-one (****A4****)*: A yellow solid was obtained with a yield of 31%. ^1^H NMR (400 MHz, CDCl_3_) δ 8.52 (dd, J = 8.1, 1.5 Hz, 1H), 7.72 (dd, J = 10.4, 5.4 Hz, 2H), 7.59 (d, J = 2.8 Hz, 1H), 7.53 (d, J = 2.0 Hz, 1H), 7.34 (ddd, J = 8.6, 6.9, 1.6 Hz, 1H), 7.16 (dd, J = 8.2, 6.5 Hz, 2H), 6.83 – 6.78 (m, 2H), 6.66 (d, J = 8.6 Hz, 1H), 6.57 (d, J = 9.1 Hz, 1H), 3.28 – 3.20 (m, 2H), 2.52 – 2.38 (m, 8H), 2.22 (s, 3H), 1.92 – 1.80 (m, 2H), 1.78 (dd, J = 12.8, 6.4 Hz, 2H). ^13^C NMR (101 MHz, CDCl_3_) δ 177.75, 154.65, 146.89, 143.85, 142.87, 136.66, 134.22, 132.53, 129.51, 127.22, 125.91, 122.97, 122.94, 122.49, 120.91, 120.68, 118.24, 116.71, 113.73, 107.00, 105.24, 57.38, 55.29, 53.21, 46.02, 44.16, 25.50. HRMS (EI): m/z [M+H]^+^ calcd. for C_29_H_30_N_4_O_2_: 467.2442. Found: 467.2443.

*10-(Benzofuran-5-yl)-2-((3-morpholinopropyl)amino)acridin-9(10H)-one (****A5****)*: A yellow solid was obtained with a yield of 47%.^1^H NMR (400 MHz, CDCl_3_) δ 8.61 (dd, J = 8.1, 1.4 Hz, 1H), 7.83 (d, J = 2.2 Hz, 1H), 7.80 (d, J = 8.6 Hz, 1H), 7.70 (d, J = 2.8 Hz, 1H), 7.62 (d, J = 2.0 Hz, 1H), 7.44 (ddd, J = 8.6, 6.9, 1.6 Hz, 1H), 7.25 (ddd, J = 11.5, 7.6, 1.5 Hz, 2H), 6.94 – 6.87 (m, 2H), 6.76 (d, J = 8.6 Hz, 1H), 6.66 (d, J = 9.1 Hz, 1H), 3.79 – 3.71 (m, 4H), 3.35 (t, J = 6.5 Hz, 2H), 2.53 (dd, J = 14.7, 8.0 Hz, 6H), 1.90 – 1.85 (m, 2H). ^13^C NMR (101 MHz, CDCl_3_) δ 177.76, 154.66, 146.91, 143.66, 142.89, 136.73, 134.19, 132.58, 129.52, 127.22, 125.89, 122.93, 122.48, 120.92, 120.73, 118.29, 116.73, 113.75, 107.00, 105.32, 67.04, 57.64, 53.78, 43.78, 25.32. HRMS (EI): m/z [M+H]^+^ calcd. for C_28_H_27_N_3_O_3_: 454.2125. Found: 454.2125.

*10-(Benzofuran-5-yl)-2-((2-morpholinoethyl)amino)acridin-9(10H)-one (****A6****)*: A yellow solid was obtained with a yield of 43%. ^1^H NMR (400 MHz, CDCl_3_) δ 8.52 (dd, J = 8.1, 1.4 Hz, 1H), 7.73 (dd, J = 10.3, 5.4 Hz, 2H), 7.63 (d, J = 2.8 Hz, 1H), 7.53 (d, J = 2.0 Hz, 1H), 7.35 (ddd, J = 8.6, 6.9, 1.6 Hz, 1H), 7.16 (ddd, J = 9.4, 6.6, 4.6 Hz, 2H), 6.86 (dd, J = 9.1, 2.9 Hz, 1H), 6.83 – 6.77 (m, 1H), 6.67 (d, J = 8.7 Hz, 1H), 6.58 (d, J = 9.1 Hz, 1H), 3.78 – 3.60 (m, 4H), 3.24 (t, J = 5.8 Hz, 2H), 2.74 – 2.55 (m, 2H), 2.52 – 2.33 (m, 4H). ^13^C NMR (101 MHz, CDCl_3_) δ 177.77, 154.67, 146.92, 143.47, 142.92, 136.87, 134.17, 132.62, 129.54, 127.22, 125.88, 122.92, 122.88, 122.55, 120.93, 120.77, 118.30, 116.75, 113.77, 107.01, 105.68, 66.93, 57.05, 53.36, 40.35. HRMS (EI): m/z [M+H]^+^ calcd. for C_27_H_25_N_3_O_3_: 440.1969. Found: 440.1969.

1*0-(Benzofuran-5-yl)-2-((3-(pyrrolidin-1-yl)propyl)amino)acridin-9(10H)-one (****A7****)*: A yellow solid was obtained with a yield of 41%. ^1^H NMR (400 MHz, CDCl_3_) δ 8.52 (dd, J = 8.1, 1.4 Hz, 1H), 7.72 (dd, J = 10.6, 5.4 Hz, 2H), 7.62 (d, J = 2.8 Hz, 1H), 7.52 (d, J = 2.0 Hz, 1H), 7.34 (ddd, J = 8.6, 6.9, 1.6 Hz, 1H), 7.18 – 7.10 (m, 2H), 6.86 (dd, J = 9.1, 2.9 Hz, 1H), 6.81 (dd, J = 2.1, 0.8 Hz, 1H), 6.66 (d, J = 8.6 Hz, 1H), 6.57 (d, J = 9.1 Hz, 1H), 4.35 (s, 1H), 3.25 (dd, J = 10.8, 5.3 Hz, 2H), 2.75 – 2.65 (m, 2H), 2.50 (dd, J = 13.9, 8.8 Hz, 4H), 1.93 – 1.80 (m, 2H), 1.75 – 1.68 (m, 4H). ^13^C NMR (126 MHz, CDCl_3_) δ 177.78, 154.66, 146.88, 143.70, 142.89, 136.77, 134.20, 132.54, 129.52, 127.22, 125.90, 122.94, 122.89, 122.71, 120.92, 120.69, 118.23, 116.72, 113.74, 107.01, 105.41, 54.77, 53.94, 42.82, 23.49. HRMS (EI): m/z [M]^+^ calcd. for C_28_H_27_N_3_O_2_: 437.2098. Found: 437.2098.

*10-(Benzofuran-5-yl)-2-((2-(dimethylamino)ethyl)amino)acridin-9(10H)-one (****A8****)*: A yellow solid was obtained with a yield of 35%. ^1^H NMR (400 MHz, CDCl_3_) δ 8.57 (d, J = 8.2 Hz, 1H), 7.81 (dd, J = 14.4, 5.3 Hz, 2H), 7.62 – 7.54 (m, 2H), 7.45 (t, J = 7.2 Hz, 1H), 7.24 (t, J = 7.8 Hz, 2H), 7.17 (dd, J = 9.2, 2.5 Hz, 1H), 6.90 (d, J = 1.5 Hz, 1H), 6.78 (d, J = 8.7 Hz, 1H), 6.69 (d, J = 9.2 Hz, 1H), 3.89 – 3.66 (m, 2H), 3.55 – 3.28 (m, 2H), 2.92 (s, 6H). ^13^C NMR (101 MHz, CDCl_3_) δ 177.78, 154.66, 146.89, 143.64, 142.89, 136.81, 134.19, 132.54, 129.52, 127.22, 125.90, 122.94, 122.89, 122.82, 120.93, 120.71, 118.25, 116.73, 113.75, 107.02, 105.27, 57.89, 45.18, 41.49. HRMS (EI): m/z [M+H]^+^ calcd. for C_25_H_23_N_3_O_2_: 398.1863. Found: 398.1863.

*10-(Benzofuran-5-yl)-2-((2-(diethylamino)ethyl)amino)acridin-9(10H)-one (****A9****)*: A yellow solid was obtained with a yield of 29%. ^1^H NMR (400 MHz, CDCl_3_) δ 8.57 (dd, J = 20.2, 7.6 Hz, 1H), 7.81 (dd, J = 12.6, 5.3 Hz, 2H), 7.60 (dd, J = 10.5, 2.1 Hz, 2H), 7.45 (t, J = 7.8 Hz, 1H), 7.24 (dd, J = 9.0, 4.6 Hz, 2H), 7.12 (dd, J = 9.1, 2.7 Hz, 1H), 6.91 (s, 1H), 6.78 (d, J = 8.7 Hz, 1H), 6.77 – 6.57 (m, 1H), 3.82 – 3.54 (m, 2H), 3.32 – 3.18 (m, 2H), 3.10 (dd, J = 14.3, 7.1 Hz, 4H), 1.33 (t, J = 7.2 Hz, 6H). ^13^C NMR (101 MHz, CDCl_3_) δ 177.78, 154.65, 146.89, 143.82, 142.90, 136.76, 134.20, 132.53, 129.52, 127.22, 125.90, 122.94, 122.69, 120.92, 120.67, 118.21, 116.72, 113.74, 107.02, 105.65, 51.45, 46.65, 41.66, 11.75. HRMS (EI): m/z [M+H]^+^ calcd. for C_27_H_27_N_3_O_2_: 426.2176. Found: 426.2176.

*10-(Benzofuran-5-yl)-2-((2-(pyridin-2-yl)ethyl)amino)acridin-9(10H)-one (****A10****)*: A yellow solid was obtained with a yield of 37%. ^1^H NMR (400 MHz, CDCl_3_) δ 8.49 (dd, J = 17.3, 6.4 Hz, 2H), 7.70 (ddd, J = 17.2, 13.5, 7.0 Hz, 3H), 7.57 – 7.42 (m, 2H), 7.38 – 7.30 (m, 1H), 7.19 – 7.07 (m, 3H), 7.06 (dd, J = 7.3, 5.2 Hz, 1H), 7.02 – 6.71 (m, 2H), 6.65 (d, J = 8.7 Hz, 1H), 6.55 (d, J = 9.1 Hz, 1H), 3.57 (t, J = 6.5 Hz, 2H), 3.06 (t, J = 6.5 Hz, 2H). ^13^C NMR (101 MHz, CDCl_3_) δ 177.75, 159.79, 154.65, 149.28, 146.89, 143.23, 142.90, 136.80, 136.67, 134.17, 132.57, 129.51, 127.32, 127.21, 125.88, 123.43, 122.93, 122.55, 121.54, 120.91, 120.71, 118.30, 116.72, 113.74, 107.01, 105.74, 43.96, 37.10. HRMS (EI): m/z [M+H]^+^ calcd. for C_28_H_21_N_3_O_2_: 454.1526. Found: 454.1526.

*N-(10-(benzofuran-5-yl)-9-oxo-9,10-dihydroacridin-2-yl)-4-morpholinobutanamide (****A11****)*: A yellow solid was obtained with a yield of 36%. ^1^H NMR (400 MHz, CDCl_3_) δ 9.35 (s, 1H), 8.57 (d, J = 8.0 Hz, 1H), 8.44 (d, J = 2.4 Hz, 1H), 8.20 (dd, J = 9.3, 2.1 Hz, 1H), 7.83 (dd, J = 8.7, 5.3 Hz, 2H), 7.63 (d, J = 1.8 Hz, 1H), 7.49 (t, J = 7.2 Hz, 1H), 7.29 – 7.23 (m, 2H), 6.92 (s, 1H), 6.79 (t, J = 8.3 Hz, 2H), 4.11 – 3.60 (m, 4H), 2.60 (dd, J = 13.8, 6.8 Hz, 8H), 2.18 – 1.86 (m, 2H). ^13^C NMR (101 MHz, CDCl_3_) δ 177.98, 171.53, 154.77, 147.06, 143.42, 140.29, 133.68, 133.35, 133.24, 129.69, 127.36, 127.04, 125.62, 122.79, 121.71, 121.49, 121.17, 117.97, 117.13, 116.43, 113.95, 107.03, 66.26, 57.76, 53.28, 35.09, 21.52. HRMS (EI): m/z [M+H]^+^ calcd. for C_29_H_27_N_3_O_4_: 482.2074. Found: 482.2075.

*N-(10-(benzofuran-5-yl)-9-oxo-9,10-dihydroacridin-2-yl)-3-(4-methylpiperidin-1-yl) propenamide (****A12****)*: A yellow solid was obtained with a yield of 39%. ^1^H NMR (400 MHz, CDCl_3_) δ 11.07 (s, 1H), 8.56 (d, J = 6.9 Hz, 1H), 8.41 (d, J = 2.3 Hz, 1H), 8.05 (dd, J = 9.3, 2.4 Hz, 1H), 7.92 – 7.69 (m, 2H), 7.63 (d, J = 1.8 Hz, 1H), 7.46 (dd, J = 11.3, 4.2 Hz, 1H), 7.28 – 7.18 (m, 2H), 6.91 (d, J = 1.4 Hz, 1H), 6.76 (t, J = 9.1 Hz, 2H), 3.25 (d, J = 11.0 Hz, 2H), 2.98 (t, J = 5.9 Hz, 2H), 2.82 – 2.77 (m, 2H), 2.38 (t, J = 10.4 Hz, 2H), 1.82 (d, J = 9.6 Hz, 2H), 1.67 – 1.46 (m, 3H), 1.03 (d, J = 4.5 Hz, 3H). ^13^C NMR (101 MHz, CDCl_3_) δ 177.78, 170.14, 154.73, 146.99, 143.33, 140.19, 133.79, 133.16, 129.65, 127.14, 126.97, 125.70, 122.84, 121.80, 121.38, 121.24, 117.85, 116.97, 116.31, 113.91, 107.04, 53.64, 53.13, 33.27, 32.32, 30.16, 21.45. HRMS (EI): m/z [M+H]^+^ calcd. for C_30_H_29_N_3_O_3_: 480.2282. Found: 480.2283.

*N-(10-(benzofuran-5-yl)-9-oxo-9,10-dihydroacridin-2-yl)-3-(diethylamino)propen amide (****A13****)*: A yellow solid was obtained with a yield of 31%. ^1^H NMR (400 MHz, CDCl_3_) δ 11.45 (s, 1H), 8.57 (dd, J = 8.1, 1.5 Hz, 1H), 8.27 – 8.13 (m, 2H), 7.91 – 7.72 (m, 2H), 7.61 (dd, J = 5.8, 1.9 Hz, 1H), 7.50 – 7.43 (m, 1H), 7.29 – 7.23 (m, 2H), 6.90 (d, J = 1.4 Hz, 1H), 6.74 (ddd, J = 13.2, 9.3, 3.4 Hz, 2H), 2.87 – 2.79 (m, 2H), 2.73 (q, J = 7.1 Hz, 4H), 2.60 – 2.50 (m, 2H), 1.18 (t, J = 7.1 Hz, 6H). ^13^C NMR (101 MHz, CDCl_3_) δ 177.81, 171.19, 154.74, 147.00, 143.36, 140.12, 133.83, 133.37, 133.16, 129.65, 127.16, 127.04, 125.71, 122.84, 121.88, 121.37, 121.26, 117.92, 116.95, 115.85, 113.91, 107.02, 48.76, 46.09, 33.01, 11.49. HRMS (EI): m/z [M+H]^+^ calcd. for C_28_H_27_N_3_O_3_: 454.2125. Found: 454.2125.

*N-(10-(benzofuran-5-yl)-9-oxo-9,10-dihydroacridin-2-yl)-3-(dimethylamino) propenamide (****A14****)*: A yellow solid was obtained with a yield of 27%. ^1^H NMR (400 MHz, CDCl_3_) δ 10.93 (s, 1H), 8.59 (d, J = 2.1 Hz, 1H), 8.21 (d, J = 2.4 Hz, 1H), 8.18 – 8.09 (m, 1H), 7.81 (dd, J = 10.6, 5.4 Hz, 2H), 7.62 (d, J = 1.9 Hz, 1H), 7.49 (dd, J = 9.1, 2.3 Hz, 1H), 7.24 (dd, J = 8.6, 2.0 Hz, 1H), 6.91 (d, J = 1.5 Hz, 1H), 6.72 (d, J = 9.3 Hz, 1H), 6.65 (d, J = 9.2 Hz, 1H), 2.89 (t, J = 5.9 Hz, 2H), 2.70 (t, J = 5.9 Hz, 2H), 2.54 (s, 6H). ^13^C NMR (101 MHz, CDCl_3_) δ 176.63, 170.71, 154.81, 147.16, 142.06, 140.00, 135.94, 133.60, 133.42, 129.75, 129.41, 127.54, 125.45, 122.69, 122.35, 121.73, 119.06, 117.99, 116.27, 114.72, 114.06, 107.04, 54.98, 44.46, 33.17. HRMS (EI): m/z [M+H]^+^ calcd. for C_26_H_23_N_3_O_3_:426.1812. Found: 426.1810.

*N-(10-(benzofuran-5-yl)-9-oxo-9,10-dihydroacridin-2-yl)-3-(4-methylpiperazin-1-yl)propenamide (****A15****)*: A yellow solid was obtained with a yield of 55%. ^1^H NMR (400 MHz, CDCl_3_) δ 11.09 (s, 1H), 8.58 (d, J = 8.0 Hz, 1H), 8.28 (d, J = 2.5 Hz, 1H), 8.19 (dd, J = 9.3, 2.5 Hz, 1H), 7.91 – 7.74 (m, 2H), 7.64 (d, J = 1.9 Hz, 1H), 7.52 – 7.45 (m, 1H), 7.29 – 7.24 (m, 2H), 6.92 (d, J = 1.9 Hz, 1H), 6.86 – 6.71 (m, 2H), 2.81 – 2.78 (m, 2H), 2.69 (s, 4H), 2.60 – 2.54 (m, 2H), 2.43 (s, 3H), 2.32 – 2.18 (m, 4H). ^13^C NMR (101 MHz, CDCl_3_) δ 177.81, 170.76, 154.75, 147.03, 143.37, 140.17, 133.78, 133.28, 133.22, 129.67, 127.11, 126.98, 125.68, 122.82, 121.81, 121.43, 121.23, 117.95, 117.01, 116.00, 113.94, 107.03, 55.16, 53.63, 52.18, 45.87, 32.52. HRMS (EI): m/z [M+H]^+^ calcd. for C_29_H_28_N_4_O_3_: 481.2234. Found: 481.2235.

*N-(10-(benzofuran-5-yl)-9-oxo-9,10-dihydroacridin-2-yl)-3-morpholinopropanamide (****A16****)*: A yellow solid was obtained with a yield of 50%. ^1^H NMR (400 MHz, CDCl_3_) δ 10.86 (s, 1H), 8.57 (d, J = 8.1 Hz, 1H), 8.24 (d, J = 2.7 Hz, 1H), 8.17 (dd, J = 9.2, 2.7 Hz, 1H), 7.96 – 7.76 (m, 2H), 7.62 (d, J = 2.1 Hz, 1H), 7.48 (t, J = 7.8 Hz, 1H), 7.24 (s, 2H), 6.90 (d, J = 2.2 Hz, 1H), 6.77 (d, J = 9.0 Hz, 2H), 3.88 (t, J = 4.6 Hz, 4H), 2.81 (t, J = 5.9 Hz, 2H), 2.68 (s, 4H), 2.61 (t, J = 5.9 Hz, 2H). ^13^C NMR (101 MHz, CDCl_3_) δ 177.78, 170.56, 154.76, 147.05, 143.39, 140.25, 133.76, 133.28, 133.10, 129.68, 127.14, 126.97, 125.67, 122.81, 121.81, 121.50, 121.26, 118.02, 117.02, 116.03, 113.96, 107.02, 67.04, 54.24, 52.92, 32.22. HRMS (EI): m/z [M+H]^+^ calcd. for C_28_H_25_N_3_O_4_: 468.1918. Found: 468.1918.

**2.1.2. General procedure for preparation of B1-B14**

The bromine-substituted compound **3b** (100 mg, 0.213 mmol), t-BuOK (120 mg, 0.768 mmol), Xantphos (74 mg, 0.128 mmol), and Pd(OAC)_2_ (19.2 mg, 0.0856 mmol) were dissolved in 1,4-dioxane (10 mL). Then compound with different amino/amide side chain (3.0 equiv) was added, and the reaction mixture was heat to 100 °C for 10 h under a nitrogen atmosphere. After being cooled down to room temperature, 10 mL of ice water was added. Dichloromethane (10 mL) was added, and the mixture was filtered. The filtrate was extracted with dichloromethane (10 mL) for two times. Then combined organic layers were washed with brine for three times, dried over anhydrous sodium sulfate, filtered, concentrated and purified using chromatography on silica gel with methanol/ dichloromethane (20/1-5/1, 0.5% NH_3_.H_2_O) to give the desire intermediate **B1-B14**.

*10-(Benzofuran-5-yl)-2,7-bis((3-(diethylamino) propyl) amino) acridin-9(10H)-one (****B1****)*: A yellow solid was obtained with a yield of 29%. ^1^H NMR (400 MHz, CDCl_3_) δ 7.81 (d, J = 2.2 Hz, 1H), 7.77 (d, J = 8.7 Hz, 1H), 7.68 (d, J = 2.8 Hz, 2H), 7.61 (d, J = 1.9 Hz, 1H), 7.25 (dd, J = 8.6, 2.0 Hz, 1H), 6.90 (d, J = 1.7 Hz, 1H), 6.86 (dd, J = 9.1, 2.8 Hz, 2H), 6.64 (d, J = 9.1 Hz, 2H), 3.33 (t, J = 6.3 Hz, 4H), 2.59 (dt, J = 14.2, 6.8 Hz, 12H), 1.88 – 1.83 (m, 4H), 1.07 (t, J = 7.1 Hz, 12H). ^13^C NMR (101 MHz, CDCl_3_) δ 177.05, 154.57, 146.76, 143.14, 136.35, 134.52, 129.32, 126.08, 123.04, 122.45, 121.99, 118.01, 113.50, 106.99, 104.85, 51.83, 46.84, 43.93, 25.71, 11.36. HRMS (EI): m/z [M+H]^+^ calcd. for C_35_H_45_N_5_O_2_: 568.3646. Found: 568.3646.

*10-(Benzofuran-5-yl)-2,7-bis((3-(dimethylamino)propyl)amino)acridin-9(10H)-one (****B2****)*: A yellow solid was obtained with a yield of 32%. ^1^H NMR (400 MHz, CDCl_3_) δ 7.72 (d, J = 2.2 Hz, 1H), 7.68 (d, J = 8.6 Hz, 1H), 7.61 (d, J = 2.8 Hz, 2H), 7.51 (d, J = 2.0 Hz, 1H), 7.15 (dd, J = 8.6, 2.1 Hz, 1H), 6.82 – 6.79 (m, 1H), 6.80 – 6.72 (m, 2H), 6.54 (d, J = 9.1 Hz, 2H), 3.23 (t, J = 6.6 Hz, 4H), 2.36 (t, J = 6.8 Hz, 4H), 2.18 (s, 12H), 1.80 – 1.71 (m, 4H). ^13^C NMR (101 MHz, CDCl_3_) δ 177.07, 154.58, 146.75, 143.06, 136.41, 134.54, 129.34, 126.08, 123.04, 122.26, 121.99, 117.98, 113.51, 107.00, 105.33, 58.18, 45.49, 43.60, 26.83. HRMS (EI): m/z [M+H]^+^ calcd. for C_31_H_37_N_5_O_2_: 512.3020. Found: 512.3019.

*10-(Benzofuran-5-yl)-2,7-bis((2-(piperazin-1-yl)ethyl)amino)acridin-9(10H)-one (****B3****)*: A yellow solid was obtained with a yield of 28%. ^1^H NMR (400 MHz, CDCl_3_) δ 7.92 (d, J = 2.8 Hz, 1H), 7.73 (d, J = 1.8 Hz, 1H), 7.69 (dd, J = 8.6, 2.8 Hz, 1H), 7.61 (d, J = 2.7 Hz, 2H), 7.51 (d, J = 1.7 Hz, 1H), 7.16 – 7.13 (m, 1H), 6.86 – 6.80 (m, 2H), 6.60 (ddd, J = 26.1, 9.2, 3.3 Hz, 2H), 4.31 (s, 2H), 3.19 (dd, J = 11.4, 5.5 Hz, 4H), 2.79 (dt, J = 12.2, 5.4 Hz, 8H), 2.58 (t, J = 5.7 Hz, 4H), 2.41 (dd, J = 15.1, 8.8 Hz, 8H). ^13^C NMR (101 MHz, CDCl_3_) δ 177.07, 154.59, 146.78, 143.28, 143.05, 136.52, 134.52, 129.37, 123.02, 122.44, 121.98, 117.93, 113.55, 106.93, 105.52, 57.27, 54.30, 46.11, 40.68. HRMS (EI): m/z [M+H]^+^ calcd. for C_33_H_39_N_7_O_2_: 566.3238. Found: 566.3241.

*10-(Benzofuran-5-yl)-2,7-bis((3-(4-methylpiperazin-1-yl)propyl)amino)anthracen-9(10H)-one (****B4****)*: A yellow solid was obtained with a yield of 46%. ^1^H NMR (400 MHz, CDCl3) δ 7.72 (d, J = 2.1 Hz, 1H), 7.68 (d, J = 8.6 Hz, 1H), 7.59 (d, J = 2.7 Hz, 2H), 7.51 (d, J = 1.9 Hz, 1H), 7.15 (dd, J = 8.6, 2.0 Hz, 1H), 6.84 – 6.72 (m, 3H), 6.55 (d, J = 9.1 Hz, 2H), 3.24 (t, J = 6.3 Hz, 4H), 2.59 – 2.32 (m, 20H), 2.23 (s, 6H), 1.82 – 1.74 (m, 4H). ^13^C NMR (101 MHz, CDCl_3_) δ 177.02, 154.57, 146.78, 143.18, 136.37, 134.53, 129.34, 126.05, 123.02, 122.39, 122.00, 118.00, 113.51, 106.98, 105.01, 57.20, 55.10, 53.00, 45.86, 44.06, 25.59. HRMS (EI): m/z [M+H]^+^ calcd. for C_33_H_47_N_7_O_2_: 622.3864. Found: 622.3864.

*10-(Benzofuran-5-yl)-2,7-bis((3-morpholinopropyl)amino)acridin-9(10H)-one (****B5****)*: A yellow solid was obtained with a yield of 33%. ^1^H NMR (400 MHz, CDCl_3_) δ 7.72 (d, J = 1.9 Hz, 1H), 7.68 (d, J = 8.6 Hz, 1H), 7.57 (d, J = 2.5 Hz, 2H), 7.51 (d, J = 1.6 Hz, 1H), 7.15 (dd, J = 8.6, 1.7 Hz, 1H), 6.80 (d, J = 2.8 Hz, 2H), 6.77 (d, J = 2.7 Hz, 1H), 6.55 (d, J = 9.1 Hz, 2H), 3.25 (t, J = 6.2 Hz, 4H), 2.56 (dd, J = 14.9, 7.2 Hz, 10H), 1.84 – 1.76 (m, 4H), 1.01 (t, J = 7.1 Hz, 10H). ^13^C NMR (101 MHz, CDCl_3_) δ 177.05, 154.61, 146.76, 143.14, 136.35, 134.52, 129.32, 126.08, 123.04, 122.45, 121.99, 118.01, 113.50, 106.99, 104.85, 51.83, 46.84, 43.93, 25.71, 11.36. HRMS (EI): m/z [M+Na]^+^ calcd. for C_35_H_41_N_5_O_4_: 618.3051. Found: 618.3053.

*10-(Benzofuran-5-yl)-2,7-bis((2-morpholinoethyl)amino)acridin-9(10H)-one (****B6****)*: A yellow solid was obtained with a yield of 35%. ^1^H NMR (400 MHz, CDCl_3_) δ 7.73 (d, J = 2.1 Hz, 1H), 7.69 (d, J = 8.6 Hz, 1H), 7.62 (d, J = 2.7 Hz, 2H), 7.51 (d, J = 1.8 Hz, 1H), 7.14 (dd, J = 8.6, 1.9 Hz, 1H), 6.92 – 6.75 (m, 3H), 6.56 (d, J = 9.1 Hz, 2H), 3.79 – 3.60 (m, 8H), 3.23 (t, J = 5.8 Hz, 4H), 2.61 (t, J = 5.8 Hz, 4H), 2.48 – 2.32 (m, 8H). ^13^C NMR (101 MHz, CDCl_3_) δ 177.07, 154.61, 146.84, 142.93, 136.57, 134.45, 129.39, 126.01, 123.00, 122.46, 121.95, 118.07, 113.59, 106.99, 105.44, 66.97, 57.11, 53.38, 40.51. HRMS (EI): m/z [M+Na]^+^ calcd. for C_33_H_37_N_5_O_4_: 590.2738. Found: 590.2740.

*10-(Benzofuran-5-yl)-2,7-bis((3-(pyrrolidin-1-yl)propyl)amino)acridin-9(10H)-one (****B7****)*: A yellow solid was obtained with a yield of 50%.^1^H NMR (400 MHz, CDCl_3_) δ 7.81 (s, 1H), 7.77 (d, J = 8.5 Hz, 1H), 7.71 (s, 2H), 7.59 (s, 1H), 7.23 (d, J = 8.2 Hz, 1H), 6.99 – 6.78 (m, 3H), 6.64 (d, J = 9.1 Hz, 2H), 4.59 – 4.15 (m, 2H), 3.50 – 3.17 (m, 4H), 2.93 – 2.72 (m, 4H), 2.68 – 2.40 (m, 8H), 2.20 – 2.10 (m, 2H), 1.94 – 1.70 (m, 8H). ^13^C NMR (101 MHz, CDCl_3_) δ 177.09, 154.58, 146.77, 143.09, 136.48, 134.50, 129.35, 126.05, 123.02, 122.59, 121.95, 117.99, 113.53, 107.00, 105.19, 54.85, 53.95, 42.97, 23.50. HRMS (EI): m/z [M]^+^ calcd. for C_35_H_41_N_5_O_2_: 563.3255. Found: 563.3236

*10-(Benzofuran-5-yl)-2,7-bis((2-(dimethylamino)ethyl)amino)acridin-9(10H)-one (****B8****)*: A yellow solid was obtained with a yield of 26%. ^1^H NMR (400 MHz, CDCl_3_) δ 7.82 (t, J = 6.6 Hz, 1H), 7.77 (d, J = 8.6 Hz, 1H), 7.68 (d, J = 2.6 Hz, 2H), 7.59 (d, J = 1.7 Hz, 1H), 7.23 (dd, J = 8.6, 1.8 Hz, 1H), 6.94 (dd, J = 9.2, 2.8 Hz, 2H), 6.89 (d, J = 1.4 Hz, 1H), 6.65 (d, J = 9.2 Hz, 2H), 3.35 (t, J = 5.7 Hz, 4H), 2.75 – 2.68 (m, 4H), 2.35 (s, 12H). ^13^C NMR (101 MHz, CDCl_3_) δ 177.03, 154.59, 146.79, 142.87, 136.52, 134.44, 129.36, 126.01, 123.00, 122.82, 121.92, 118.10, 113.55, 107.00, 104.82, 57.84, 45.02, 41.37. HRMS (EI): m/z [M+H]^+^ calcd. for C_29_H_33_N_5_O_2_: 484.2707. Found: 484.2701.

*10-(Benzofuran-5-yl)-2,7-bis((2-(diethylamino)ethyl)amino)acridin-9(10H)-one (****B9****)*: A yellow solid was obtained with a yield of 39%. ^1^H NMR (400 MHz, CDCl_3_) δ 7.72 (d, J = 2.1 Hz, 1H), 7.68 (d, J = 8.6 Hz, 1H), 7.59 (d, J = 2.7 Hz, 2H), 7.51 (d, J = 1.9 Hz, 1H), 7.14 (dd, J = 8.6, 2.0 Hz, 1H), 6.85 (dd, J = 9.1, 2.8 Hz, 2H), 6.80 (d, J = 1.7 Hz, 1H), 6.56 (d, J = 9.2 Hz, 2H), 3.25 (t, J = 5.7 Hz, 4H), 2.74 (t, J = 5.7 Hz, 4H), 2.57 (dd, J = 14.2, 7.1 Hz, 8H), 1.00 (t, J = 7.1 Hz, 12H). ^13^C NMR (101 MHz, CDCl_3_) δ 177.06, 154.59, 146.79, 142.93, 136.51, 134.45, 129.35, 126.02, 123.01, 122.80, 121.92, 118.10, 113.54, 107.00, 105.00, 51.48, 46.81, 41.41, 11.29. HRMS (EI): m/z [M+H]^+^ calcd. for C_33_H_41_N_5_O_2_: 540.3333. Found: 540.3337.

*10-(Benzofuran-5-yl)-2,7-bis((2-(pyridin-2-yl)ethyl)amino)acridin-9(10H)-one (****B10****)*: A yellow solid was obtained with a yield of 41%. ^1^H NMR (400 MHz, CDCl_3_) δ 8.46 (d, J = 4.5 Hz, 2H), 7.75 – 7.69 (m, 1H), 7.70 – 7.60 (m, 3H), 7.55 (tt, J = 7.2, 3.6 Hz, 2H), 7.48 (d, J = 1.9 Hz, 1H), 7.15 (d, J = 7.8 Hz, 2H), 7.10 (ddd, J = 12.3, 7.8, 3.6 Hz, 3H), 6.80 (dd, J = 9.4, 2.7 Hz, 3H), 6.53 (d, J = 9.2 Hz, 2H), 3.58 (t, J = 6.5 Hz, 4H), 3.08 (t, J = 6.5 Hz, 4H). ^13^C NMR (101 MHz, CDCl_3_) δ 177.00, 159.70, 154.57, 148.93, 146.80, 142.49, 136.98, 136.55, 134.40, 129.36, 125.99, 123.60, 122.99, 122.56, 121.92, 121.62, 118.10, 113.54, 107.00, 105.53, 44.11, 36.97. HRMS (EI): m/z [M+Na]^+^ calcd. for C_35_H_29_N_5_O_2_: 574.2213. Found: 574.2213.

*N,N'-(10-(benzofuran-5-yl)-9-oxo-9,10-dihydroacridine-2,7-diyl)bis(4-morpholino butanamide) (****B11****)*: A yellow solid was obtained with a yield of 35%. ^1^H NMR (400 MHz, DMSO) δ 10.24 (s, 2H), 8.69 (s, 2H), 8.23 (s, 1H), 7.98 (d, J = 8.6 Hz, 1H), 7.85 (s, 1H), 7.78 (d, J = 8.0 Hz, 2H), 7.39 (d, J = 8.2 Hz, 1H), 7.12 (s, 1H), 6.71 (d, J = 9.2 Hz, 2H), 3.62 – 3.48 (m, 8H), 2.41 – 2.25 (m, 16H), 1.81 – 1.68 (m, 4H). ^13^C NMR (101 MHz, DMSO) δ 176.68, 171.61, 154.61, 148.38, 139.51, 134.02, 133.91, 129.90, 126.54, 126.19, 123.40, 121.08, 117.99, 115.27, 114.37, 107.79, 66.67, 58.21, 53.74, 34.74, 22.40. HRMS (EI): m/z [M+H]^+^ calcd. for C_37_H_41_N_5_O_6_: 652.3130. Found: 652.3137.

*N,N'-(10-(benzofuran-5-yl)-9-oxo-9,10-dihydroacridine-2,7-diyl)bis(3-(pyrrolidin-1-yl)propanamide) (****B12****)*: A yellow solid was obtained with a yield of 32%. ^1^H NMR (400 MHz, CDCl_3_) δ 11.35 (s, 2H), 8.19 (dd, J = 9.3, 2.5 Hz, 2H), 8.08 (d, J = 2.5 Hz, 2H), 7.83 (d, J = 2.2 Hz, 1H), 7.81 (d, J = 8.6 Hz, 1H), 7.66 (d, J = 2.0 Hz, 1H), 7.29 (d, J = 3.3 Hz, 1H), 6.92 (d, J = 1.6 Hz, 1H), 6.77 (d, J = 9.3 Hz, 2H), 2.95 – 2.84 (m, 4H), 2.79 – 2.70 (m, 8H), 2.65 – 2.51 (m, 4H), 1.98 – 1.87 (m, 8H). ^13^C NMR (101 MHz, CDCl_3_) δ 177.27, 171.11, 154.75, 146.97, 139.89, 133.81, 133.26, 129.61, 127.02, 125.70, 122.84, 121.15, 117.77, 115.87, 113.84, 107.03, 53.26, 51.42, 34.55, 23.74. HRMS (EI): m/z [M=Na]^+^ calcd. for C_35_H_37_N_5_O_4_: 614.2738. Found: 614.2732.

*N,N'-(10-(benzofuran-5-yl)-9-oxo-9,10-dihydroacridine-2,7-diyl)bis(3-(diethylami no)propanamide) (****B13****)*: A yellow solid was obtained with a yield of 38%. ^1^H NMR (400 MHz, CDCl_3_) δ 11.16 (s, 2H), 8.30 (d, J = 2.2 Hz, 2H), 8.19 – 8.05 (m, 2H), 7.88 – 7.74 (m, 2H), 7.58 (dd, J = 14.9, 1.9 Hz, 1H), 7.35 – 7.30 (m, 0.4H), 7.22 (dd, J = 8.6, 2.0 Hz, 0.6H), 6.88 (d, J = 2.0 Hz, 1H), 6.71 (dd, J = 17.0, 9.3 Hz, 2H), 3.08 – 3.00 (m, 4H), 2.87 (dd, J = 13.9, 6.8 Hz, 8H), 2.82 – 2.71 (m, 4H), 1.23 (t, J = 7.2 Hz, 12H). ^13^C NMR (101 MHz, CDCl_3_) δ 177.38, 170.99, 154.75, 147.00, 139.91, 133.22, 127.03, 125.66, 122.80, 121.25, 117.88, 117.72, 115.74, 115.65, 113.87, 107.02, 48.74, 46.16, 32.92, 11.37. HRMS (EI): m/z [M+H]^+^ calcd. for C_35_H_41_N_5_O_4_: 596.3231. Found: 596.3220.

*N,N'-(10-(benzofuran-5-yl)-9-oxo-9,10-dihydroacridine-2,7-diyl)bis(3-morpholino propanamide) (****B14****)*: A yellow solid was obtained with a yield of 29%. ^1^H NMR (400 MHz, DMSO) δ 10.26 (s, 2H), 8.67 (d, J = 2.4 Hz, 2H), 8.22 (d, J = 2.1 Hz, 1H), 7.98 (d, J = 8.6 Hz, 1H), 7.85 (d, J = 2.0 Hz, 1H), 7.75 (dd, J = 9.3, 2.5 Hz, 2H), 7.40 (dd, J = 8.6, 2.1 Hz, 1H), 7.13 (d, J = 1.4 Hz, 1H), 6.73 (d, J = 9.2 Hz, 2H), 3.66 – 3.49 (m, 8H), 3.46 – 3.20 (m, 8H), 2.74 – 2.62 (m, 4H), 2.53 – 2.40 (m, 4H). ^13^C NMR (101 MHz, DMSO) δ 176.66, 170.47, 154.63, 148.41, 139.62, 133.85, 129.91, 126.57, 126.19, 123.40, 121.08, 118.15, 115.31, 114.39, 107.80, 66.58, 54.64, 53.46, 34.25. HRMS (EI): m/z [M+H]^+^ calcd. for C_35_H_37_N_5_O_6_: 624.2817. Found: 624.2815.

**3. Other Supporting Table, Spectra and Graphs**

**Table S2.** Effect of compounds on Aβ_1-42_ aggregation


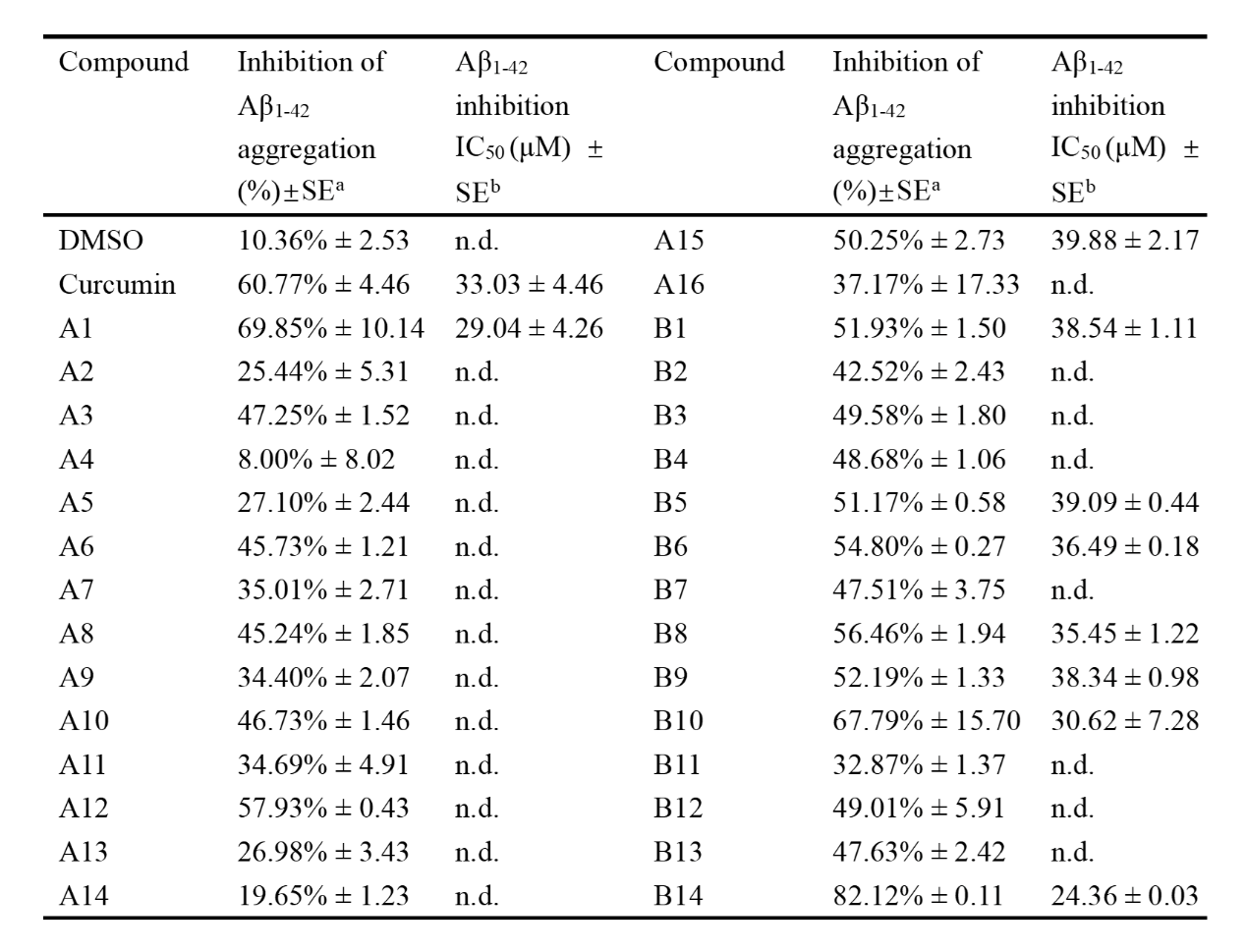


n.d. = not determined

^a^ Self-induced Aβ_1-42_ aggregation (means ± SD of three experiments). The thioflavin-T fluorescence method was used, and the measurements were carried out in the presence of 40 μM test compound.

^b^ IC_50_ values of test compounds represent the concentration that inhibited 50% Aβ_1-42_ aggregation

**Table S3**. Equilibrium binding constants (*K_D_*) determined by using SPR

| 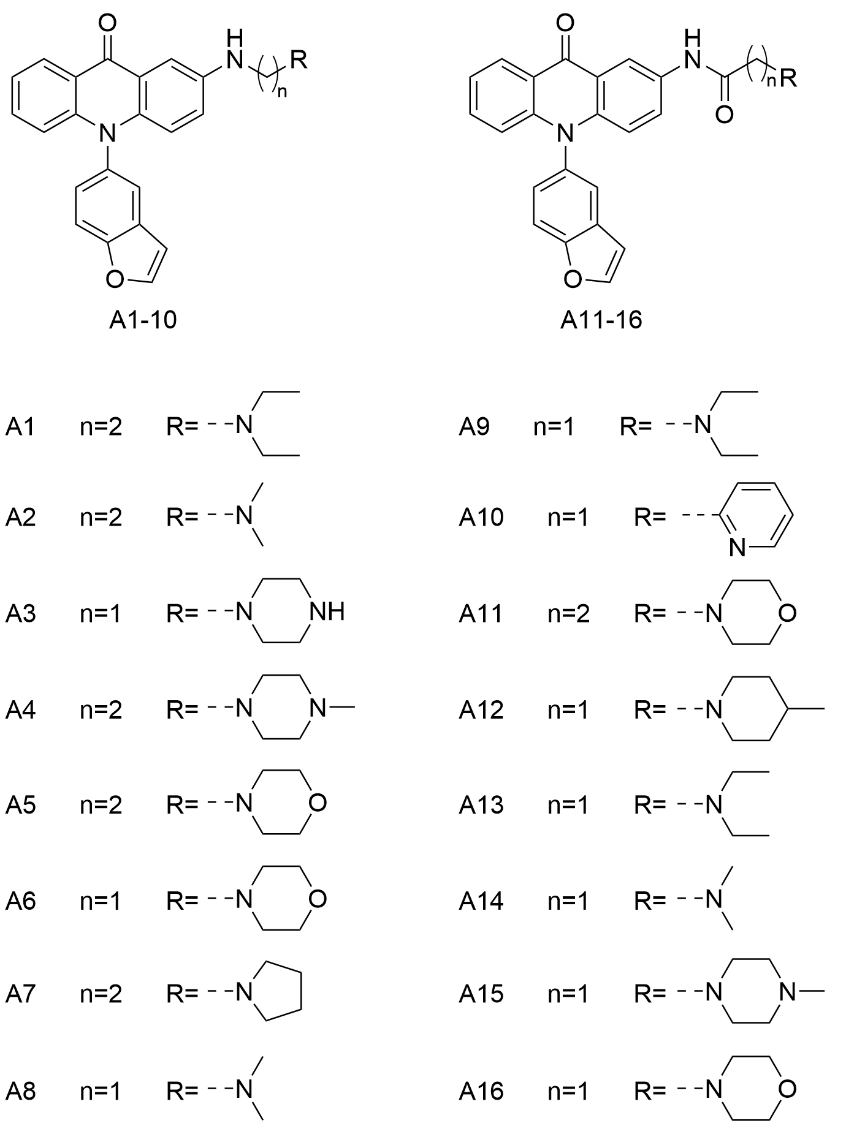 | Compound | *K*_D_ (μM) |
| --- | --- | --- |
|  | **A1** | >50 |
|  | **A2** | >50 |
|  | **A3** | >50 |
|  | **A4** | >50 |
|  | **A5** | >50 |
|  | **A6** | >50 |
|  | **A7** | >50 |
|  | **A8** | >50 |
|  | **A9** | 34.1 |
|  | **A10** | >50 |
|  | **A11** | >50 |
|  | **A12** | >50 |
|  | **A13** | >50 |
|  | **A14** | >50 |
|  | **A15** | >50 |
|  | **A16** | >50 |
|  |  |  |

**Table S4**. Equilibrium binding constants (*K_D_*) determined by using SPR

| 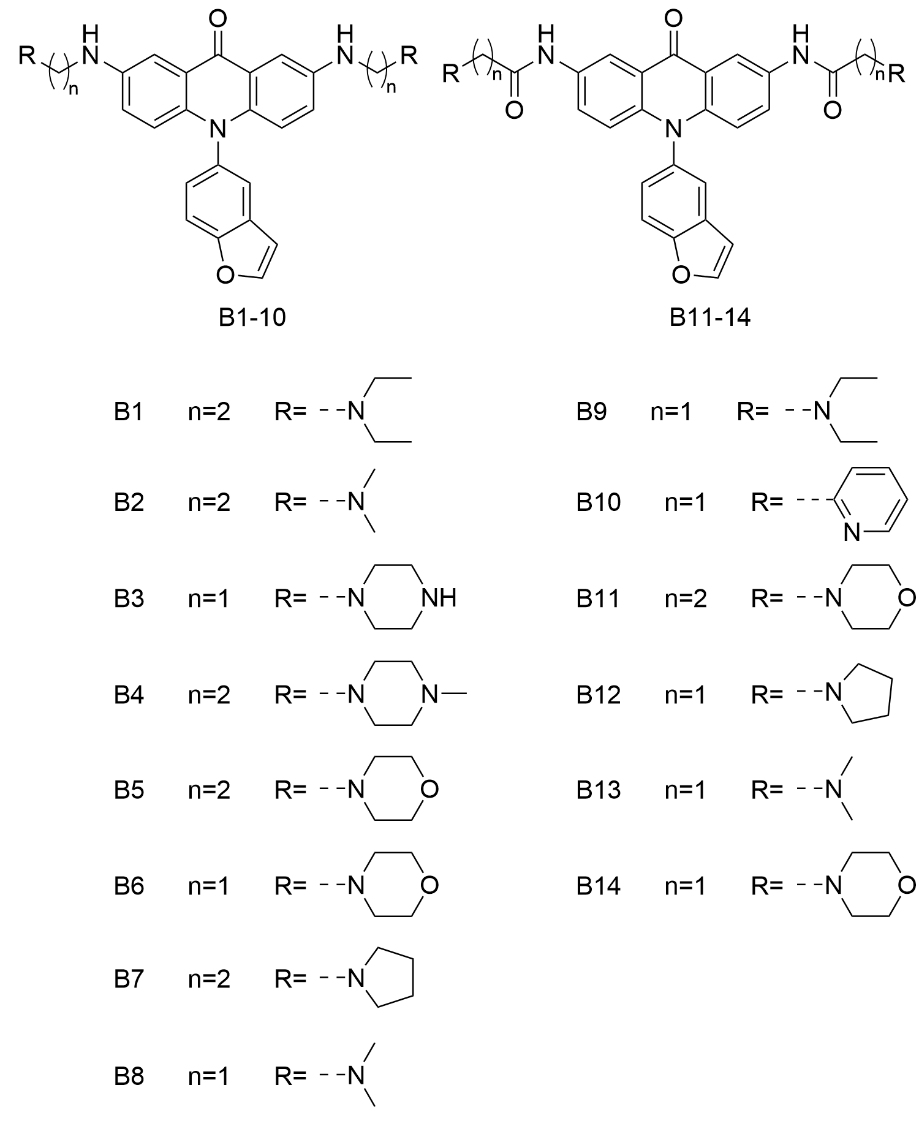 | Compound | *K*_D_ (μM) |
| --- | --- | --- |
|  | **B1** | >50 |
|  | **B2** | >50 |
|  | **B3** | >50 |
|  | **B4** | >50 |
|  | **B5** | >50 |
|  | **B6** | >50 |
|  | **B7** | >50 |
|  | **B8** | >50 |
|  | **B9** | >50 |
|  | **B10** | >50 |
|  | **B11** | >50 |
|  | **B12** | >50 |
|  | **B13** | >50 |
|  | **B14** | 2.13 |
|  |  |  |
|  |  |  |
|  |  |  |

**Table S5.** Relative TO displacement ratio (%) of selected compounds after screening through SPR experiment

| **Compound** | **Relative displacement ratio (%)** |
| --- | --- |
| **B14** | 65.7 |
| **A22** | 62.6 |

**Table S6**. IC_50_ values for effect of **B14** on various cell lines determined by using MTT after incubation for 48 h

| Cell lines | IC_50_ (μM) |
| --- | --- |
| SH-SY5Y | >100 |
| HT-22 | >100 |
| bEnd.3 | >100 |
| HK-2 | >100 |

**Table S7.** Primers sequence information

| **Spics** | **Gens** | **Forward primer (5’-3’)** | **Reverse primer (5’-3’)** |
| --- | --- | --- | --- |
| human | *β-actin* | CTGGAACGGTGAAGGTGAA | AAGGGACTTCTGTAACAACGA |
|  | *BCL-2* | TGTTGTTCAAACGGGATTCA | GGCTGGGCACATTTACTGTT |
|  | *BAX* | AGCGACTGATGTCCCTGTCT | CTCAGCCCATCTTCTTCCAG |
|  | *VEGF* | GCTACTGCCATCCAATCGAG | CTTGGTGAGGTTTGATCCGC |
|  | *C-MYC* | AAACACAAACTTGAACAGCTAC | ATTTGAGGCAGTTTACATTATGG |
| mice | Bcl-2 | GTACCTGAACCGGCATCTG | GGGGCCATATAGTTCCACAA |
|  | Bax | TAGCAAACTGGTGCTCAAGG | TCTTGGATCCAGACAAGCAG |
|  | β-actin | GACCTCTATGCCAACACAGTGC | GTACTCCTGCTTGCTGATCCAC |

**Table S8.** The qPCR raw data from the real-time RT-PCR assay for cells and animal samples

| **Experiment 1** | **Ct** | | **^a^-∆∆Ct ^a^** | **^b^F = 2^-∆∆Ct^** |
| --- | --- | --- | --- | --- |
|  | **β-actin** | **BCL-2** |  |  |
| Control | 17.99 | 18.88 | 0 | 1 |
| *6.25 μM* ***B14*** | 17.22 | 17.80 | -0.31 | 1.23 |
| *12.5 μM* ***B14*** | 17.35 | 17.51 | -0.73 | 1.65 |
| *25 μM* ***B14*** | 17.49 | 17.27 | -1.11 | 2.15 |
|  | **Ct** | | **^a^-∆∆Ct ^a^** | **^b^F = 2^-∆∆Ct^** |
|  | **β-actin** | **BAX** |  |  |
| Control | 17.99 | 18.87 | 0 | 1 |
| *6.25 μM* ***B14*** | 17.22 | 17.97 | -0.13 | 1.09 |
| *12.5 μM* ***B14*** | 17.35 | 18.23 | 0 | 1.00 |
| *25 μM* ***B14*** | 17.49 | 18.34 | -0.03 | 1.02 |
|  | **Ct** | | **^a^-∆∆Ct ^a^** | **^b^F = 2^-∆∆Ct^** |
|  | **β-actin** | **VEGF** |  |  |
| Control | 17.99 | 19.41 | 0 | 1 |
| *6.25 μM* ***B14*** | 17.22 | 18.63 | -0.01 | 1.00 |
| *12.5 μM* ***B14*** | 17.35 | 18.76 | -0.01 | 1.00 |
| *25 μM* ***B14*** | 17.49 | 18.88 | -0.03 | 1.02 |
|  | **Ct** | | **^a^-∆∆Ct ^a^** | **^b^F = 2^-∆∆Ct^** |
|  | **β-actin** | **C-MYC** |  |  |
| Control | 17.99 | 19.68 | 0 | 1 |
| *6.25 μM* ***B14*** | 17.22 | 18.9 | -0.01 | 1.00 |
| *12.5 μM* ***B14*** | 17.35 | 19.02 | -0.02 | 1.01 |
| *25 μM* ***B14*** | 17.49 | 19.15 | -0.03 | 1.02 |

| **Experiment 2** | **Ct** | | **^a^-∆∆Ct ^a^** | **^b^F = 2^-∆∆Ct^** |
| --- | --- | --- | --- | --- |
|  | ***β-actin*** | ***BCL-2*** |  |  |
| *Control* | 15.52 | 21.18 | -0.01 | 1 |
| *25 μM Aβ_1-42_* | 16.07 | 22.3 | 0.56 | 0.68 |
| *25 μM Aβ_1-42_+6.25 μM* ***B14*** | 15.88 | 21.5 | -0.05 | 1.04 |
| *25 μM Aβ_1-42_+12.5 μM* ***B14*** | 15.91 | 21.1 | -0.48 | 1.40 |
| *25 μM Aβ_1-42_+25 μM* ***B14*** | 16.1 | 20.95 | -0.82 | 1.76 |
|  | ***β-actin*** | ***BAX*** | **^a^-∆∆Ct ^a^** | **^b^F = 2^-∆∆Ct^** |
| *Control* | 15.52 | 21.88 | -0.01 | 1 |
| *25 μM Aβ_1-42_* | 16.07 | 20.16 | -2.27 | 4.82 |
| *25 μM Aβ_1-42_+6.25 μM* ***B14*** | 15.88 | 20.53 | -1.71 | 3.23 |
| *25 μM Aβ_1-42_+12.5 μM* ***B14*** | 15.91 | 21.18 | -1.09 | 2.13 |
| *25 μM Aβ_1-42_+25 μM* ***B14*** | 16.1 | 21.66 | -0.80 | 1.71 |

| **Experiment 3** | **Ct** | | **^a^-∆∆Ct ^a^** | **^b^F = 2^-∆∆Ct^** |
| --- | --- | --- | --- | --- |
|  | ***β-actin*** | ***BCL-2*** |  |  |
| *WT* | 23.21 | 28.98 | 0 | 1 |
| *Model* | 23.88 | 30.21 | 0.56 | 0.67 |
| ***B14*** *(10 mg/kg)* | 23.26 | 29.42 | 0.39 | 0.76 |
| ***B14*** *(20 mg/kg)* | 23.52 | 29.46 | 0.17 | 0.88 |
| ***B14*** *(40 mg/kg)* | 23.38 | 29.14 | -0.01 | 1.00 |
| *Donepezil* *(10 mg/kg)* | 23.71 | 29.74 | 0.26 | 0.83 |
|  | ***β-actin*** | ***BAX*** | **^a^-∆∆Ct ^a^** | **^b^F = 2^-∆∆Ct^** |
| *WT* | 23.21 | 28.88 | 0.08 | 0.94 |
| *Model* | 23.88 | 29.33 | -0.22 | 1.16 |
| ***B14*** *(10 mg/kg)* | 23.26 | 28.77 | -0.16 | 1.11 |
| ***B14*** *(20 mg/kg)* | 23.52 | 29.27 | 0.08 | 0.94 |
| ***B14*** *(40 mg/kg)* | 23.38 | 29.26 | 0.21 | 0.86 |
| *Donepezil* *(10 mg/kg)* | 23.71 | 29.31 | -0.07 | 1.05 |

^a^ **-∆∆Ct** = -[(Ct value of genes in treated sample - Ct value of genes in control) - (Ct value of β-actin in treated sample - Ct value of β-actin in control)]. ^b^ F: The relative expression fold.

Experiment 1: SH-SY5Y cells were treated with different concentration of **B14**. The Ct values of BAX, VEGF, CMYC and BCL-2 were obtained.

Experiment 2: The Ct values of BCL-2 and Bax in 25 μM Aβ_1-42_ model with different concentration of **B14**.

Experiment 3: The Ct values of BCL-2 and Bax in WT, Model, **B14** and Donepezil administration group of mice brain.


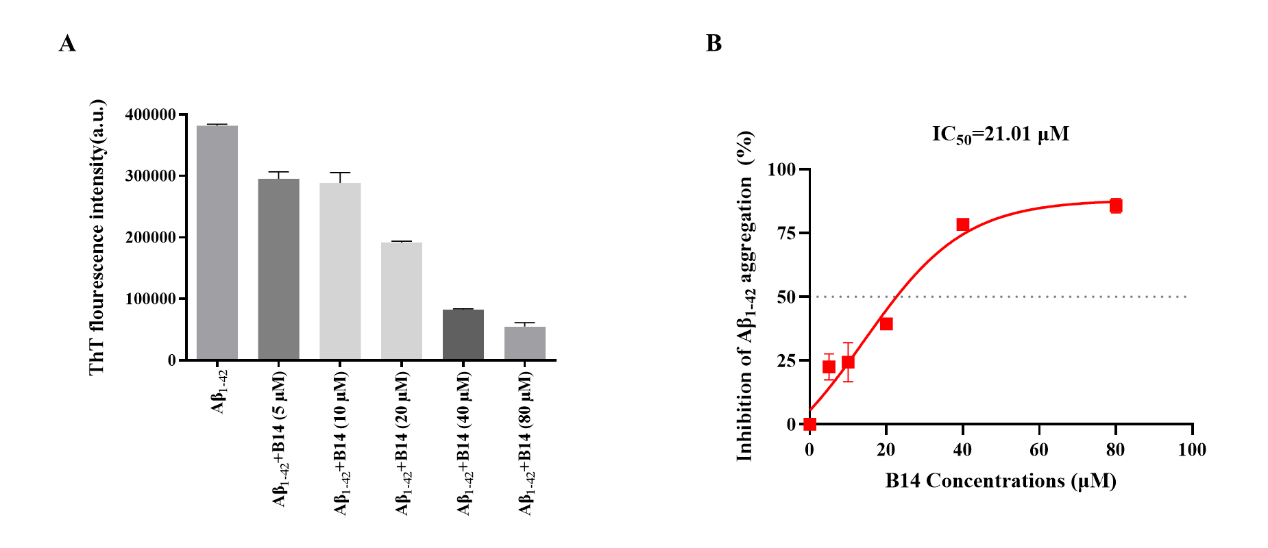
**Figure S2**. The fluorescence intensity of different concentrations of **B14** was measured after incubation with Aβ_1-42_. Data were statistically analyzed as mean ± SEM. (A) ThT fluorescence intensity. (B) IC_50_ value for **B14** against Aβ_1-42_ aggregation was determined by using ThT fluorescence intensity.


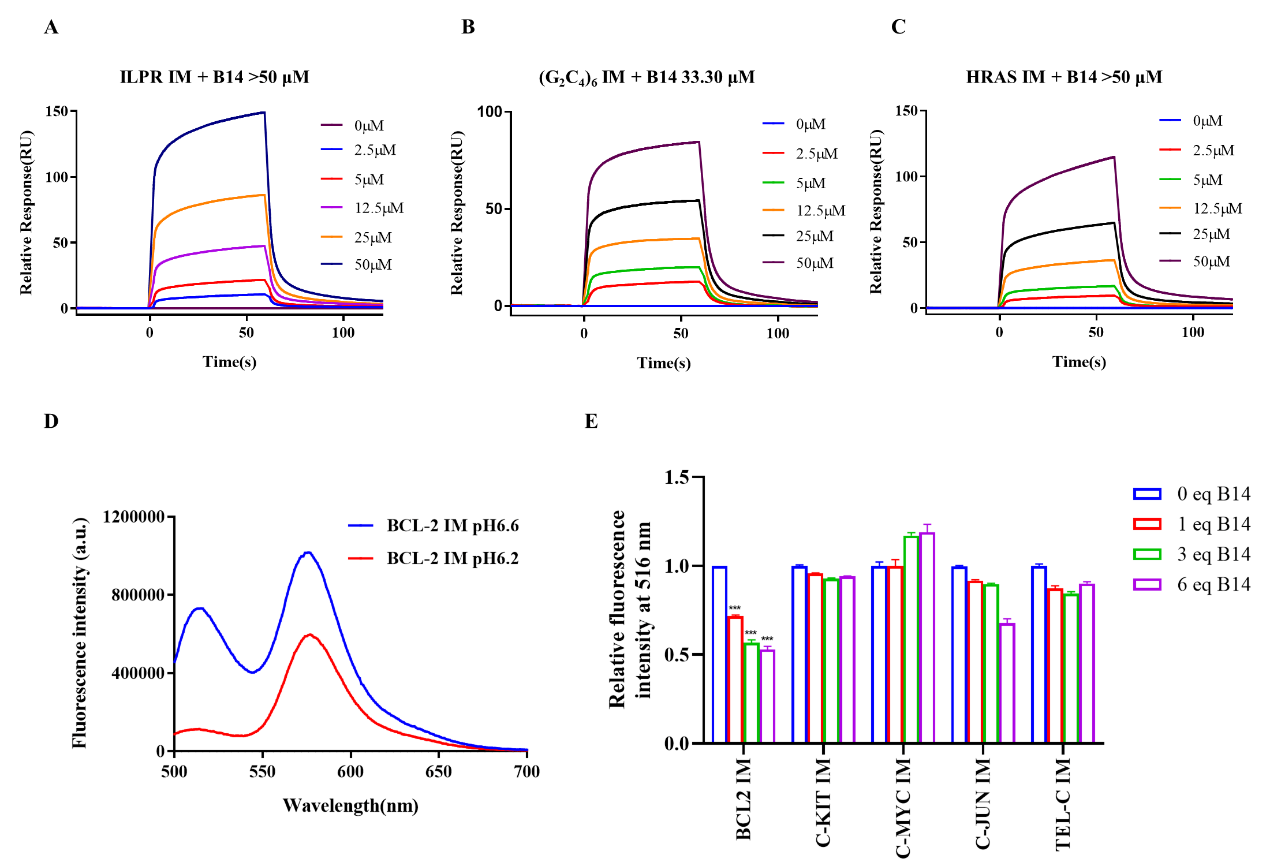


**Figure S3**. Biophysical experiments were performed for binding of acridine derivative to *BCL-2* promoter i-motif and other DNA structures. (A-C) SPR experiments were performed for binding of **B14** to *ILPR* promoter i-motif*,* *(G_2_C_4_)_6_* promoter i-motif, and *HRAS* promoter i-motif. Bio-*ILPR*, bio-(*G_2_C_4_)_6_*, and bio-*HRAS* were annealed in MES buffer at pH 5.5. (D) FRET experiment was performed for pH-dependent fluorescence change of *BCL-2* promoter i-motif dual labeled with FAM and TAMRA at the 5´-end and 3´-end, respectively. The data showed that more i-motif structures could form at lower pH, and therefore pH 6.2 was selected for the follow-up FRET titration experiments. (E) Dose-dependent fluorescence spectra changes at 516 nm for various dual labeled C-rich oligomers (representing i-motifs from promoters of *BCL-2*, *C-KIT*, *C-MYC*, *C-JUN* and *TEL-C* DNA) were measured with addition of increasing amount of **B14**. Dose-dependent decrease of absorbance at 516 nm for *BCL-2* indicated that **B14** could induce the formation of *BCL-2* promoter i-motif. All the experiments were repeated for three times.


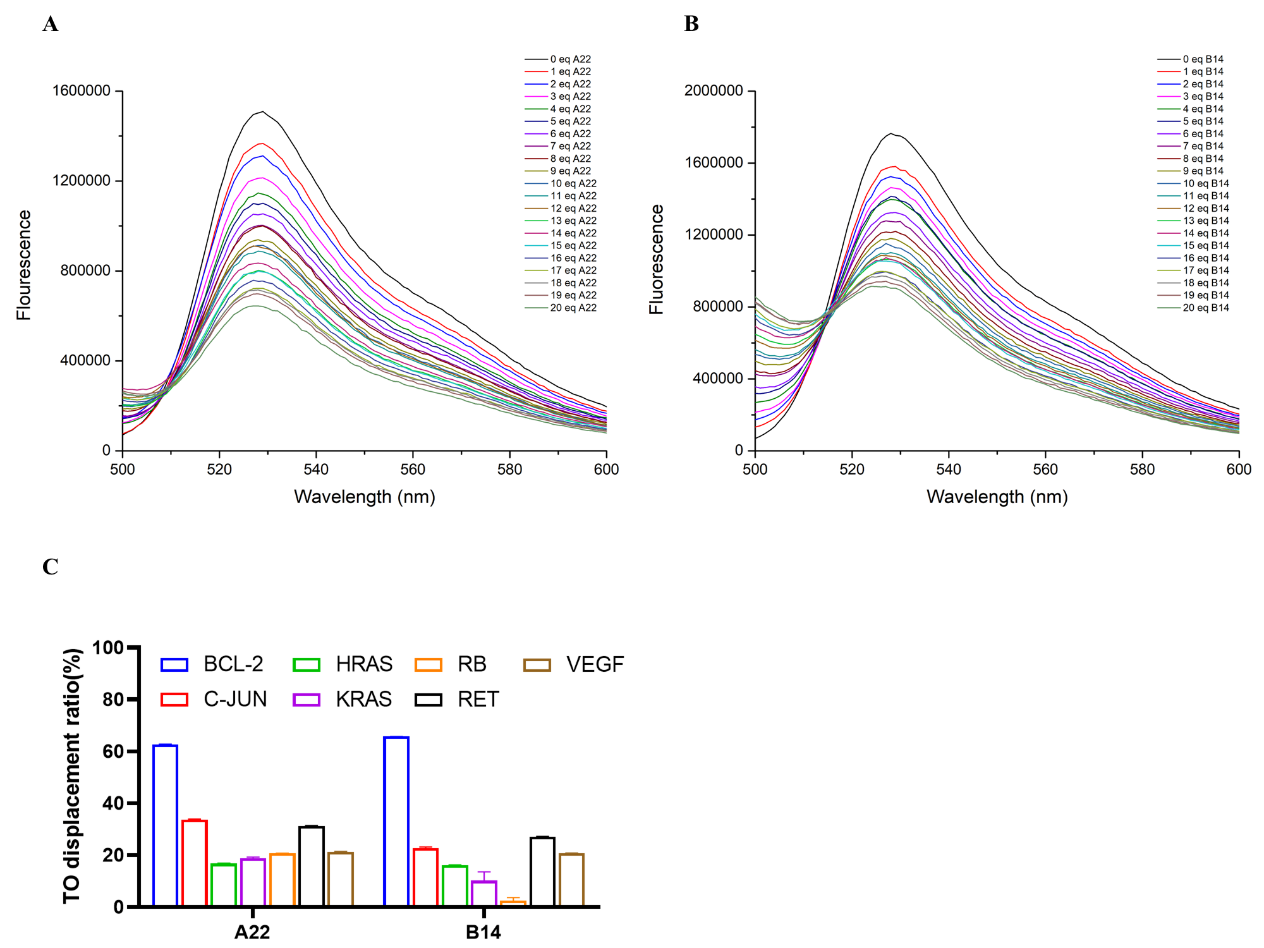


**Figure S4**. TO displacement and UV titration experiments were performed to examine the effects of **B14** and **A22** on *BCL-2* promoter i-motif. (A-B) TO displacement was performed on *BCL-2* promoter i-motif annealed in 1XBPES buffer at pH 5.5 with increasing amount of **B14** and **A22**. (C) The above relative TO displacement ratio were statistically analyzed.


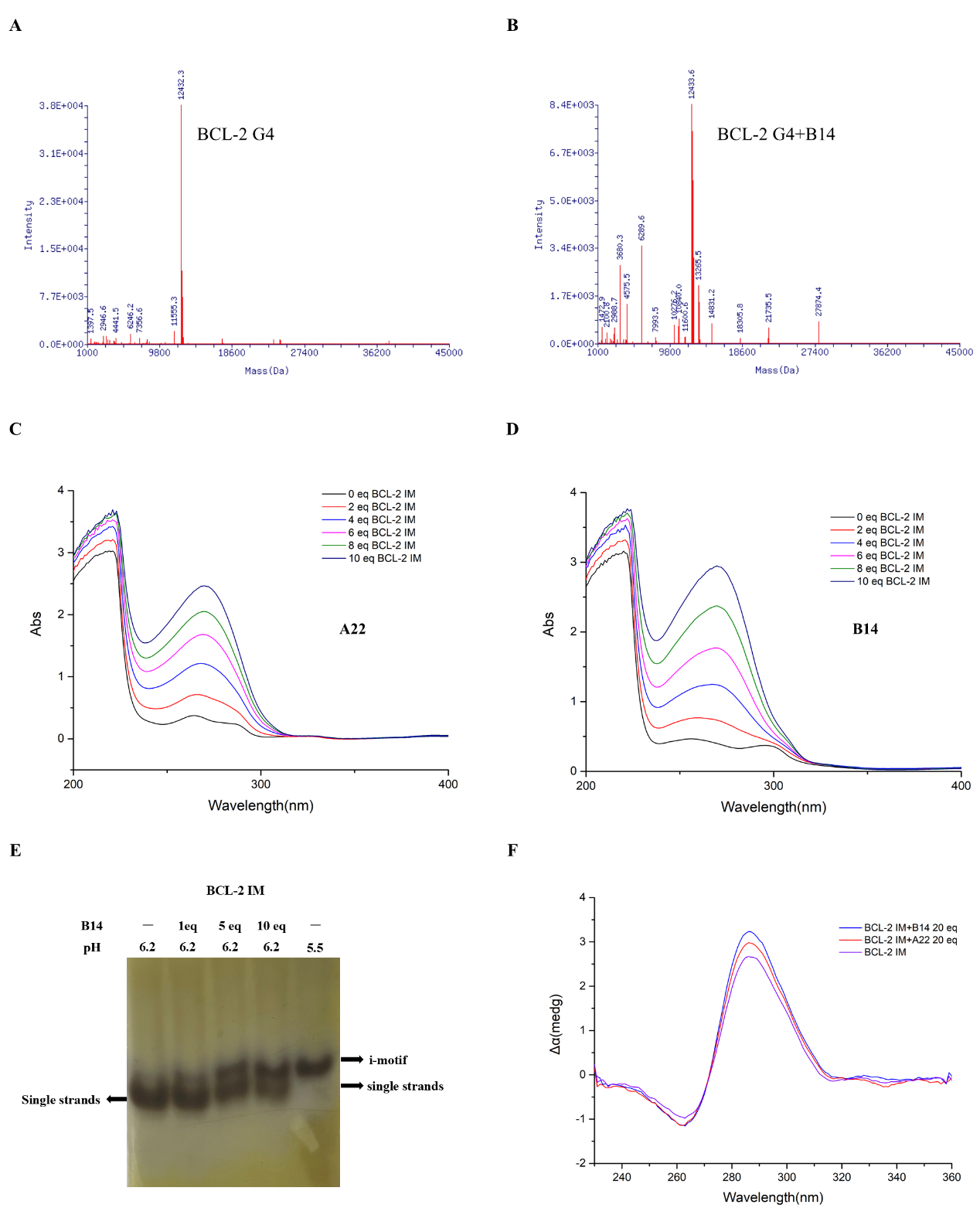


**Figure S5**. ESI-MS, UV, CD, and EMSA experiments were performed to examine the effect of **B14** on *BCL-2* promoter i-motif and G-quadruplex. (A) ESI-MS experiment for oligomer *BCL-2* G4 alone, which was annealed in 1 × BPES buffer at pH 7.4. (B) ESI-MS experiment for above oligomer *BCL-2* G4 with addition of **B14** in 1 × BPES buffer at pH 7.4. (C-D) TO displacement with increasing amount of oligomer *BCL-2* i-motif annealed in 1 X BPES buffer at pH 5.5 in the presence of 20 equivalents of **B14** or **A22**. (E) EMSA experiment was performed for the effect of **B14** on inducing *BCL-2* i-motif formation. The lanes from left to right were as follow: *BCL-2* i-motif alone at pH 5.5; *BCL-2* i-motif alone at pH 6.2; *BCL-2* i-motif plus 1 eq **B14** at pH 6.2; *BCL-2* i-motif plus 5 eq **B14** at pH 6.2; *BCL-2* i-motif plus 10 eq **B14** at pH 6.2. (F) CD experiment was performed to study the interaction of **B14** or **A22** with *BCL-2* promoter i-motif. **B14** and **A22** could increase its absorption peak at 586 nm, indicating that both compounds could induce the formation of *BCL-2* promoter i-motif, with **B14** having stronger effect than **A22**.


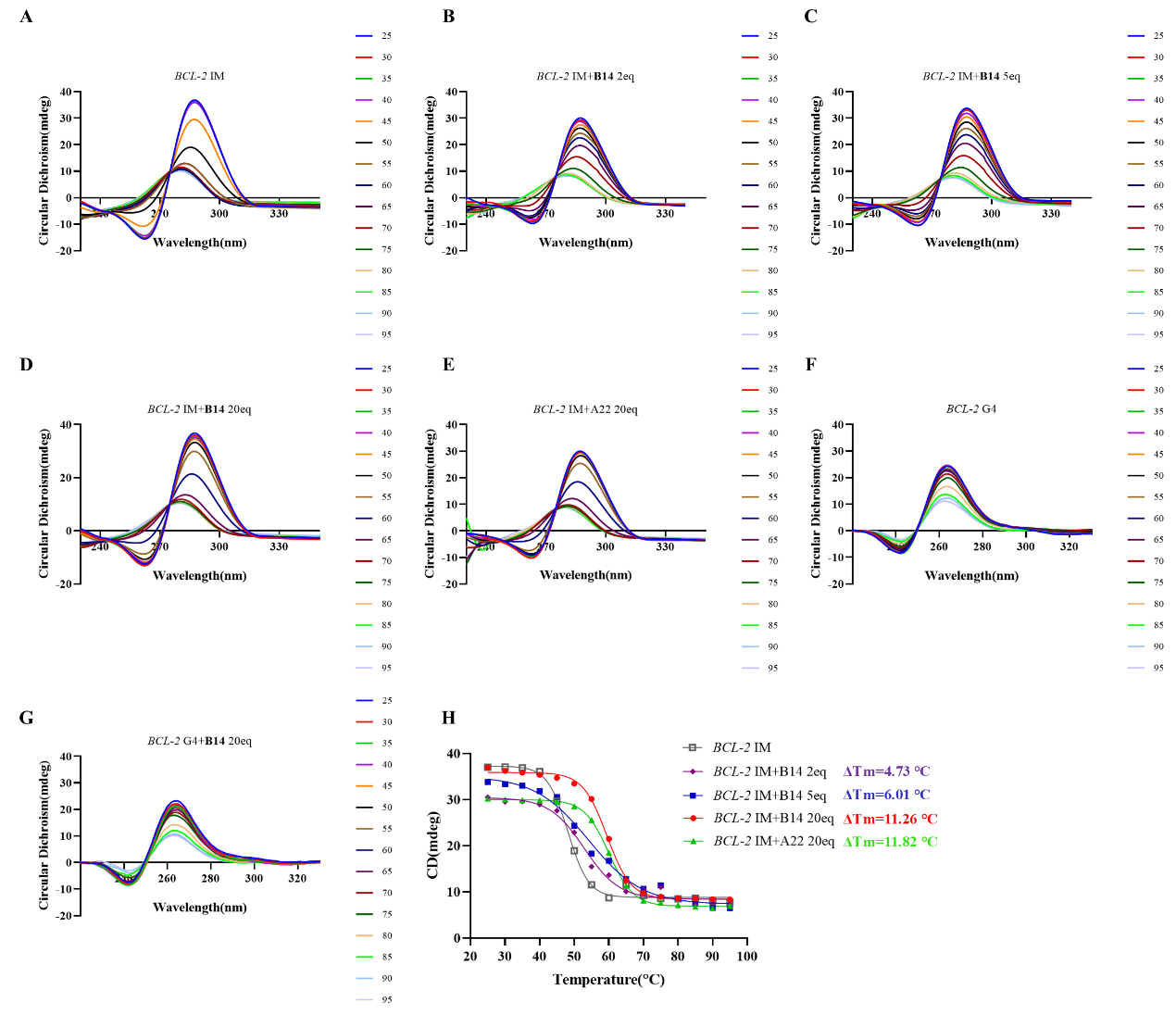


**Figure S6**. CD melting experiments were performed to examine the effects of **B14** and **A22** on *BCL-2* promoter i-motif and G-quadruplex. (A-H) CD melting experiments were performed for *BCL-2* promoter i-motif or G-quadruplex in the absence or presence of **B14** (2, 5, 20 equivalents) and **A22** (20 equivalents). The oligonucleotide for *BCL-2* i-motif was annealed in 1 X BPES buffer at pH 5.5, while the oligonucleotide for *BCL-2* G-quadruplex was annealed in 20 mM Tris-HCl buffer with 20 mM NaCl at pH 7.4. The *ΔTm* values for the effects of **B14** and **A22** on *BCL-2* promoter i-motif were determined to be 11.26 °C and 11.82 °C, respectively.


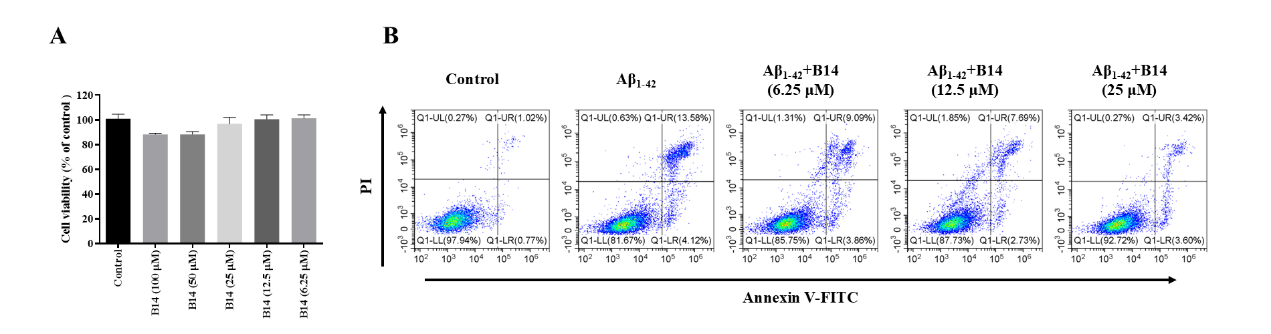


**Figure S7.** Effect of **B14** on SH-SY5Y cells incubated with Aβ_1-42_. (A) Effect of **B14** on the viability of SH-SY5Y cells. (B) Anti-apoptotic effect of **B14** on Aβ_1-42_ induced toxicity to SH-SY5Y cells were determined by using flow cytometry. All these experiments were repeated for three times.

**Figure S8.** Effect of **B14** on the body weights of APP/PS1 transgenic mice in comparison with that of donepezil.


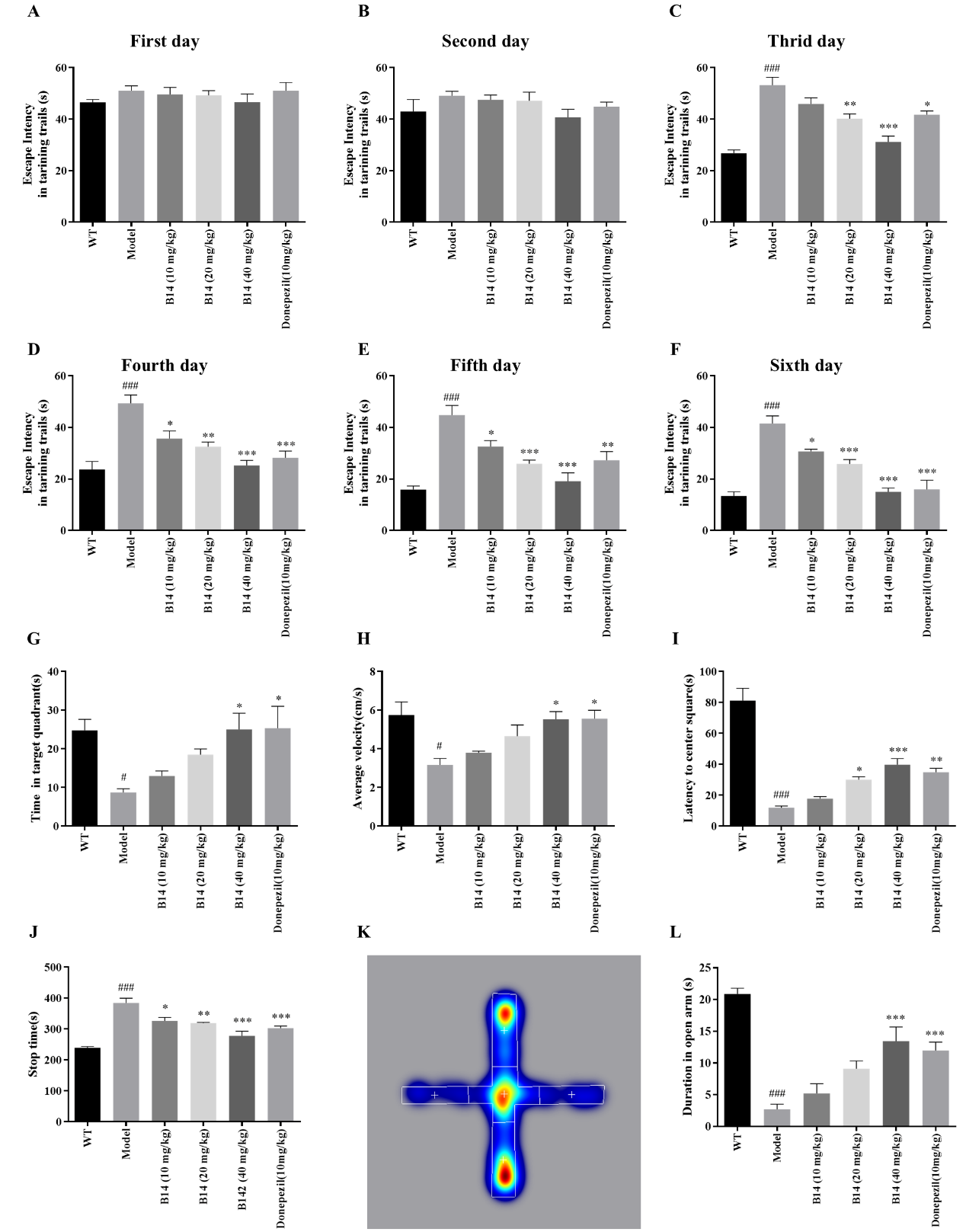


**Figure S9.** Effect of **B14** on behavioral cognitive deficits in APP/PS1 transgenic mice. (A-F) Escape latency determination in the positioning and navigation experiment. (G) Activity time in the central area of APP/PS1 mice. (H) Average velocity in APP/PS1 mice. (I) APP/PS1 mice residence time in the center square. (J) Stop time for APP/PS1 mice in open field. (K) Schematic diagram of the elevated plus maze (EPM) and time spent in the open arm by APP/PS1 mice (L). The data were expressed as the mean ± SEM: ^#^ p < 0.05, ^###^ p < 0.001 vs Control group; ^*^ p < 0.05, ^**^ p < 0.01, ^***^ p < 0.001 vs Model group, significantly different from the control.


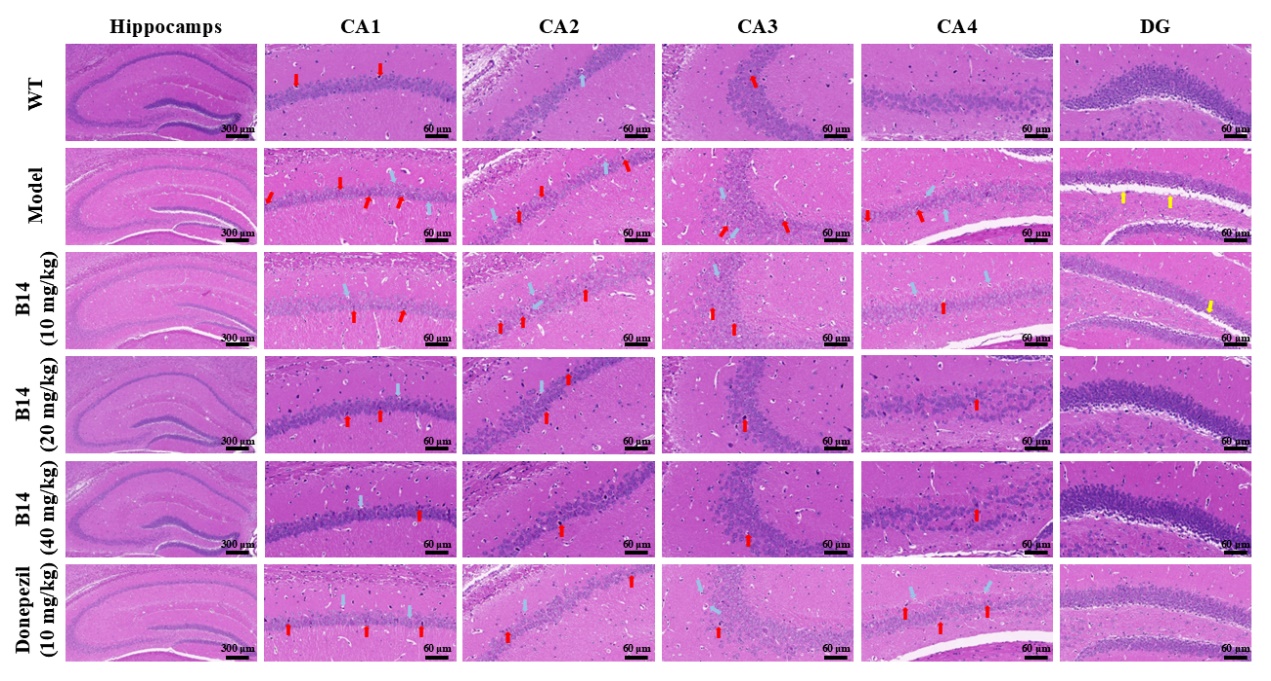


**Figure S10.** Histopathological staining showed hippocampal morphology in APP/PS1 transgenic mice, with cell edema indicated by red arrows and pyknosis indicated by blue arrows. **B14** could reduce the cell edema and pyknosis, while increasing the number of neurons in the hippocampus of AD mice. Scale bar was 300 μm and 60 μm.


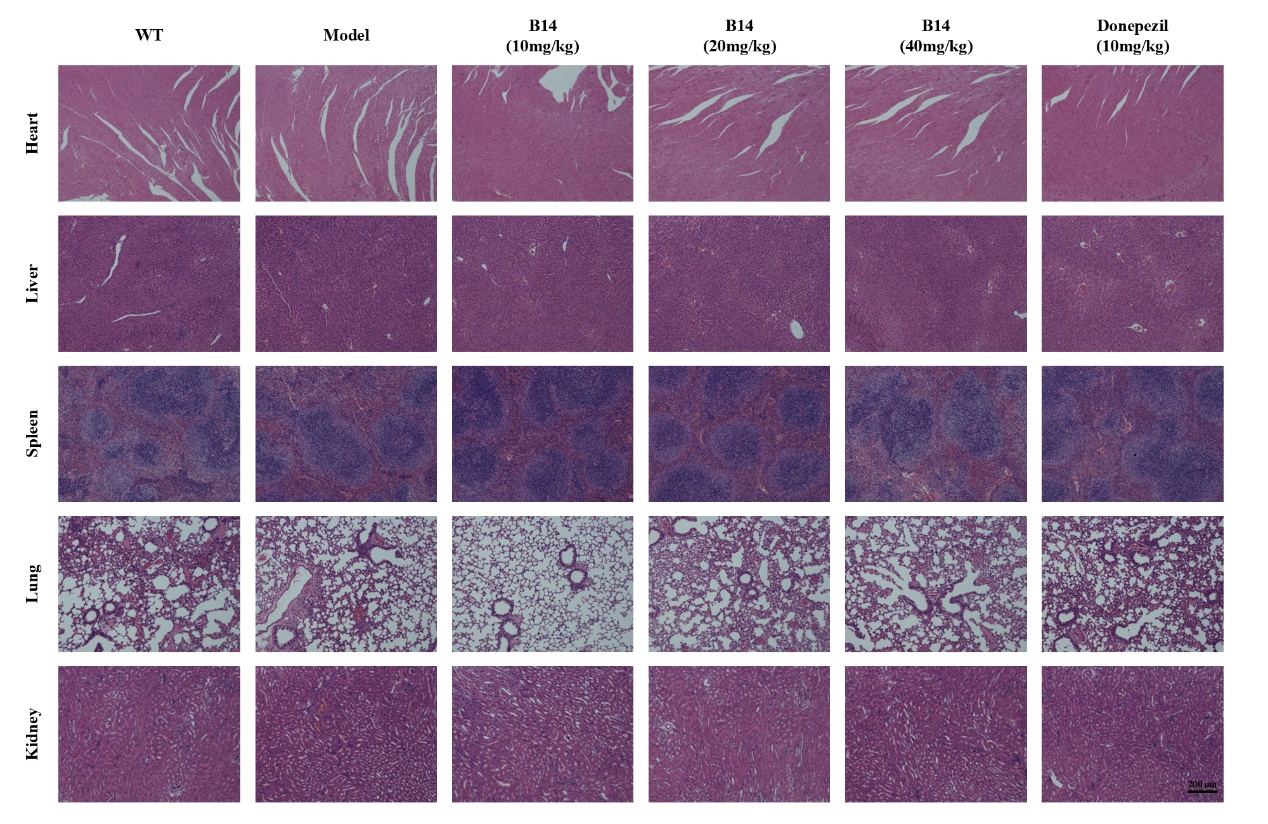


**Figure S11.** HE staining was performed for vital organs including heart, liver, spleen, lung, and kidney under various experimental conditions. Scale bar was 200 μm.

**4. MATERIAL AND METHODS**

**4.1. General materials**

Chemically synthesized DNA oligomers of HPLC purified grade were purchased from Sangon Biotec. All oligonucleotide concentrations were determined by measuring absorbance at 260 nm using a Nano Drop 1000 spectrophotometer (Thermo Scientific). For obtaining i-motif structures, C-rich oligonucleotides were annealed in 1XBPES buffer (30 mM KH_2_PO_4_, 30 mM K_2_HPO_4_, 1 mM EDTA, 100 mM KCl) of different pH at 95 °C for 5 min, and then cooled to room temperature. For obtaining G-quadruplex, oligonucleotides were annealed in 20 mM Tris-HCl buffer containing 100 mM KCl (pH 7.0) by heating at 95 °C for 5 min followed with gradual cooling to room temperature. Formation of secondary non-B DNA structures were determined by using circular dichroism (CD) spectrophotometer.

**4.2. Preparation of the Aβ_1-42_ oligomers**

Aβ_1-42_ oligomers were prepared as previously described [38]. The Aβ_1-42_ peptide (0.5 mg) was completely dissolved in dimethyl sulfoxide (0.022 mL), and then diluted with 1.058 mL of DMEM without phenol red. The Aβ_1-42_ solutions were filtered (0.22 μm filter) and incubated at 37 °C for 72 h.

**4.3. Congo red binding assay**

A total of 30 μM Congo red solution (20 mM potassium phosphate, 50 mM NaCl, pH 7.4) was prepared and filtered with a 0.45 μm filter. The Aβ_1-42_ solutions (30 μM) were co-incubated with **B14** (20 μM) and Curcumin (20 μM) at 37 °C protected from light for 72h. The Aβ_1-42_ solution was mixed with equal volume of Congo red solution and incubated for 30 min. A spectral region from 400 nm to 650 nm was analyzed by using a UV-vis spectrophotometer (AoE, China).

**4.4. Circular dichroism (CD) measurements**

Circular dichroism (CD) experiment was performed to investigate the effect of **B14** (20 μM) and Curcumin (20 μM) on the change of Aβ_1-42_ secondary structure. The Aβ_1-42_ solutions (100 μM) were co-incubated with **B14** (20 μM) and Curcumin (20 μM) at 37 °C for 72h. Ellipticity changes of Aβ_1-42_ were monitored employing a Chirascan CD spectropolarimeter (Applied Photophysics, UK).

**4.5. Thioflavine T fluorescence spectroscopy assay**

The Aβ_1-42_ solutions (100 μM) were co-incubated with **B14** (20 μM) and Curcumin (20 μM) at 37 °C protected from light for 96 h. 30 μL of Aβ_1-42_ solutions were taken at different time points (0 h, 10 h, 24 h, 48 h, 72 h, 96 h), mixed fully with 400 μL of ThT solution (15 μM), and incubated in the dark for 30 min. The fluorescence signal was recorded by a Cary Eclipse fluorescence spectrophotometer (Varian, USA) with excitation wavelength at 440 nm, emission wavelength at 480 nm, and slit width of 20 nm.

**4.6. Dot blot**

Dot blot assays were performed to detect Aβ_1-42_ fibril aggregation with **B14** and Curcumin as described previously with minor modification. Briefly, Aβ_1-42_ was diluted into 20 μM solution with buffer (20 mM HEPES, 150 mM NaCl, pH 7.4), and then incubated with or without **B14** and Curcumin (20 μM). After incubation at 37 °C for 24h, 10 μL aliquots of 20 μM Aβ_1-42_ reactions were spotted onto nitrocellulose membranes. The membranes were blocked for 2 h with 5% non-fat milk in TBS. After washing, the membranes were incubated with the anti-Aβ fibril antibody (1:1000 dilution) dissolved in TBS containing 3% BSA and 0.01% Tween-20, and then developed using an alkaline-phosphatase antirabbit secondary antibody (1:5000 dilution). Invitrogen's Western Breeze Chemiluminescent kit was used to visualize the protein dots, and these dots were imaged using a FUJIFILM Luminescent Image Analyzer LAS-1000CH.

**4.7. Transmission electron microscopy (TEM)**

The Aβ_1-42_ solution (100 μM) was co-incubated with **B14** solution (20 μM) or Curcumin (20 μM) at 37 °C for 72h. The samples were dropped to a carbon-coated grid, stained with 2% phosphotungstic acid, and then dried at room temperature. The morphology of Aβ_1-42_ fiber was observed on a JEM-1200EX II transmission electron microscope (Jeol, Japan).

**4.8. Fluorescence resonance energy transfer (FRET) melting experiment**

The oligonucleotides with fluorescent 5´-FAM and 3′-TAMRA labeling at concentration of 100 nM in 1 × BPES buffer were annealed by heating at 95 °C for 5 min followed by cooling to room temperature. The spectra from 500 nm to 700 nm of 100 μL mixture with addition of different equivalent compounds at 25 °C were recorded based on fluorescence emission (λex = 480 nm).

**4.9. Circular dichroic (CD) spectroscopy and CD-melting experiments**

CD measurements were performed on a Chirascan circular dichroism spectrophotometer (Chirascan, England). A 10 mm path length quartz cuvette was used to record the spectra with a wavelength range of 230-350 nm with a 1 nm bandwidth. CD and CD melting experiments were performed at a fixed i-motif or G-quadruplex concentration (1 μM) with or without a fixed concentration (20 μM) of **B14** and Curcumin in different buffers. Data collection was performed at intervals of 5 °C over a range of 25–95 °C with a heating rate of 2.5 ºC/min.

**4.10. TO displacement assay**

DNA oligonucleotides were diluted to 1 μM with 1 × PBES buffer at pH 5.5, annealed at 95 ºC for 10 min, cooled to room temperature, and stored at 4 ºC for future use. TO dye (1 μM) was added to the DNA solution followed with incubation for 1 h. The spectra of 100 μL mixture with addition of different equivalent compounds at 25 ºC were recorded based on fluorescence emission (λex = 480 nm).

TO displacement ratio (%) = (F_0_ − F_C_) / (F_0_ − F_B_) * 100.

F_0_ and F_C_ were the fluorescence intensity of TO at 536 nm with DNA before and after adding compounds, respectively. F_B_ was the fluorescence intensity of TO at 536 nm with buffer only.

**4.11. Ultraviolet-visible spectroscopy experiments**

*BCL-2* i-motif (500 μM) was prepared with BPES buffer at pH 5.5, annealed in a metal bath at 95 ºC for 10 min, allowed to cool down to room temperature slowly, and then stored at 4 ºC. **B14** and **A22** were prepared as 1 mL of 10 μM solutions with the corresponding buffers, and their UV absorption spectra were analyzed using a UV spectrophotometer in a scanning range of 200-400 nm. Then *BCL-2* i-motif was added dropwise at increasing concentration of 1 μM each step up to 10 μM finally, which was mixed and incubated for 10 min before UV analysis.

**4.12. Electrophoretic mobility shift assay (EMSA)**

The DNA oligonucleotides were diluted to 5 μM with 1 × BPES buffer at pH 5.5 or pH 6.2, and different equivalents of compound were added. The mixtures were annealed at 95 ºC for 10 min, gradually cooled to room temperature, and incubated at 4 ºC overnight. Electrophoresis was carried out using 20% acrylamide Native PAGE (pH 6.4) with 1 × Tris-borate-EDTA (TBE) as running buffer (pH 6.4) at 80 V for 7 h at 4 ºC. The gels were then silver-stained and photographed.

**4.13. MTT cytotoxicity assay**

SH-SY5Y cells, HepG2 cells, HK-2 cells and HT-22 cells were seeded on 96-well plates (5.0×10^3^ per well) with 100 μL of culture medium, and incubated for 12 h at 37 °C in a humidified atmosphere with 5% CO_2_, and then exposed to various concentrations of **B14**. After 48 h of treatment at 37 °C in a humidified atmosphere with 5% CO_2_, 100 μL of 0.5 mg/mL MTT solution was added to each well, and the mixture was further incubated for 4 h. The cells in each well were then treated with DMSO (200 μL for each well), and the culture medium was siphoned off, and the absorbance was recorded at 570 nm. All drug doses were parallel tested in triplicate, and the cytotoxicity was evaluated based on the percentage of cell survival in a dose-dependent manner regarding the negative control. The final IC_50_ values were determined by using Graph Pad Prism 9.0.

**4.14. RNA extraction and qRT-PCR**

Total RNA was extracted from cells and mice liver tissue using RNAiso Plus (Takara, 9109, Japan). Following sample homogenization, chloroform was added, and the solution was allowed to have phase separation into an upper aqueous layer (containing RNA), an interphase, and a lower organic layer (containing DNA and proteins). RNA was precipitated from the aqueous phase using isopropanol, while DNA was precipitated from the aqueous-organic interphase using ethanol. Complementary DNA (cDNA) was synthesized using the One-Step RT-PCR Kit (Takara, Cat#639503, Japan).

Quantitative real-time polymerase chain reaction (qRT-PCR) was performed using the 2× RealStar SYBR Mixture (GenStar, Cat# A301-10, Guangzhou, China) on a LightCycler 480 II real-time PCR system (Roche, USA). Data were analyzed using the 2^−ΔΔCt method. Primers were synthesized by Sangon Biotech (Guangzhou, China), and their sequences are listed in Table S7. Actin served as the internal control, and relative mRNA levels were normalized accordingly.

**4.15. FITC Annexin V/PI cell apoptosis analysis**

The FITC Annexin V/PI apoptosis analysis was conducted using the FITC Annexin V/PI Apoptosis Detection Kit (Muti sciences, 70-AP101-100). Cells were treated with varying concentrations of **B14** in a 6-well plate containing a cell model induced with 25 μM Aβ_1-42_. After digestion and resuspension in 500 μL binding buffer, samples were incubated with 5 μL of FITC Annexin V for 5 minutes, followed by the addition of 5 μL PI for further incubation at room temperature for 10 minutes. Flow cytometry analysis (Millipore, guava easy Cyte) was used to measure fluorescence intensity, and data analysis was performed using Flow jo V10.

**4.16. Hoechst 33342/Mito-tracker deep red/H2DCFDA/JC-1 staining and confocal imaging**

SH-SY5Y cells were plated on 96 well plates. After 24h treatment with or without **B14** in 25 μM Aβ_1-42_ induced cell model, the cells were washed for three times with cold PBS. Then cells were incubated with Hoechst 33342 solution (Sigma, 10 μg/mL) and H2DCFDA probe (10 μM, sigma), as well as Mito-tracker deep red (100 nM, sigma) for 10 min at 37 °C in the dark. All the samples were photographed by using FV3000 (Olympus). SH-SY5Y cells were plated on 6 well plates. After 24h treatment with **B14** in 25 μM Aβ_1-42_ induced cell model, cells were incubated with JC-1 (5 μg/mL, sigma) for 30 min at 37 °C in the dark. Fluorescence detection was carried out by using EVOS FL Auto.

**4.17. Primary antibodies for Western blot analysis**

Primary antibodies for Western blot analysis include BCL-2 (affinity biosciences OH, Cat# AF6139, USA), BAX (affinity biosciences OH, Cat# AF0120, USA), VEGF (affinity biosciences OH, Cat# AF5131, USA), C-MYC (affinity biosciences OH, Cat# AF0358, USA), Caspase9 (affinity biosciences OH, Cat# AF6348, USA), cleaved-Caspase9 (affinity biosciences OH, Cat# AF5240, USA), Caspase3 (affinity biosciences OH, Cat# AF6311, USA), cleaved-Caspase3 (affinity biosciences OH, Cat# AF7022, USA), and Cyto-C (affinity biosciences OH, Cat# AF6139, USA).

**4.18. Behavioral experiments**

Morris water maze (MWM) system consisted of a heated circular pool, a target platform, and a video capture with analysis system. The pool with a diameter of 110 cm and a height of 45 cm, was filled with water at 25 °C ± 1 °C, and made opaque with titanium dioxide. It was divided into four equal quadrants, and a transparent platform (10 cm in diameter) was placed in the fourth quadrant with its surface 1-1.5 cm below the water. The MWM experiment comprised two main parts including a localization trial and a spatial search trial. The localization trial lasted for 5 days, and the mice were placed in the water facing the wall from one of the four quadrants and allowed to swim for 60 seconds to find the platform. The time taken to find the platform (latency) was recorded. The mice that found the platform were allowed to rest on it for 20 seconds. Those unable to find it within 60 seconds were gently placed on the platform for 20 seconds. On the 6th day, the spatial search trial was conducted. The platform was removed, and the mice were monitored for 60 seconds. Their movements were tracked by using a computer-controlled video tracking system, which could record and analyze their behavioral data.

The open field test (OFT), also known as the mice spontaneous activity test, is commonly used to assess the spontaneous activity and exploratory behavior of mice. The setup consisted of four identical experimental boxes (60 cm × 60 cm × 50 cm) placed in a quiet room. During the experiment, each mouse was gently placed in the center of the open field chamber by holding the tip of its tail. A camera system was used to observe and record the mice's behavioral changes for 5 minutes. After the test, the chamber's interior and bottom were cleaned with 75% alcohol to eliminate any residual odors that might influence subsequent tests.

The nest building test (NBT) assesses fine motor dexterity, cognitive function, and emotional states in mice while minimizing stress. The experimental procedure was as follows: mice were individually housed in single cages with absorbent paper placed flat on top of the bedding. The mice were then allowed to move freely in the cage for 24 hours. After this period, photographs were taken of the paper sheets, and the nests built by the mice were scored.

The novel object recognition (NOR) test evaluates mice's memory for familiar objects and their exploration of novel objects. The setup included a free-roaming box (52 cm × 52 cm × 52 cm) and a camera recording system. On the first day, mice were individually acclimated to the box without any objects for 5 minutes. In the first stage, two identical objects (A1 and A2) were placed diagonally in the box, and mice were allowed to explore them freely for 5 minutes. In the second stage, object A2 was replaced with a novel object B, and the mice were allowed to explore for another 5 minutes. The time spent exploring each object was recorded. The mice's learning and memory abilities were assessed by calculating the preference index (PI) using the formula PI = tB / (tA1 + tB).

The elevated plus maze (EPM) consisted of two relatively open arms (50 cm×10 cm) and two relatively closed arms (50 cm×10 cm×40 cm) connected to a central area (10 cm×10 cm), with a video acquisition system and a video analysis system. During the experiment, the mice were placed into the central area of the maze with their heads facing the open-arm area and allowed to move freely for 5 min, while the time and number of times the mice entered the open arms were recorded.

**5. NMR spectra and HPLC profiles of compounds**

^1^H NMR spectrum of **2** (400 MHz, DMSO)





^13^C NMR spectrum of **2** (101 MHz, DMSO)





^1^H NMR spectrum of **3a** (400 MHz, DMSO)


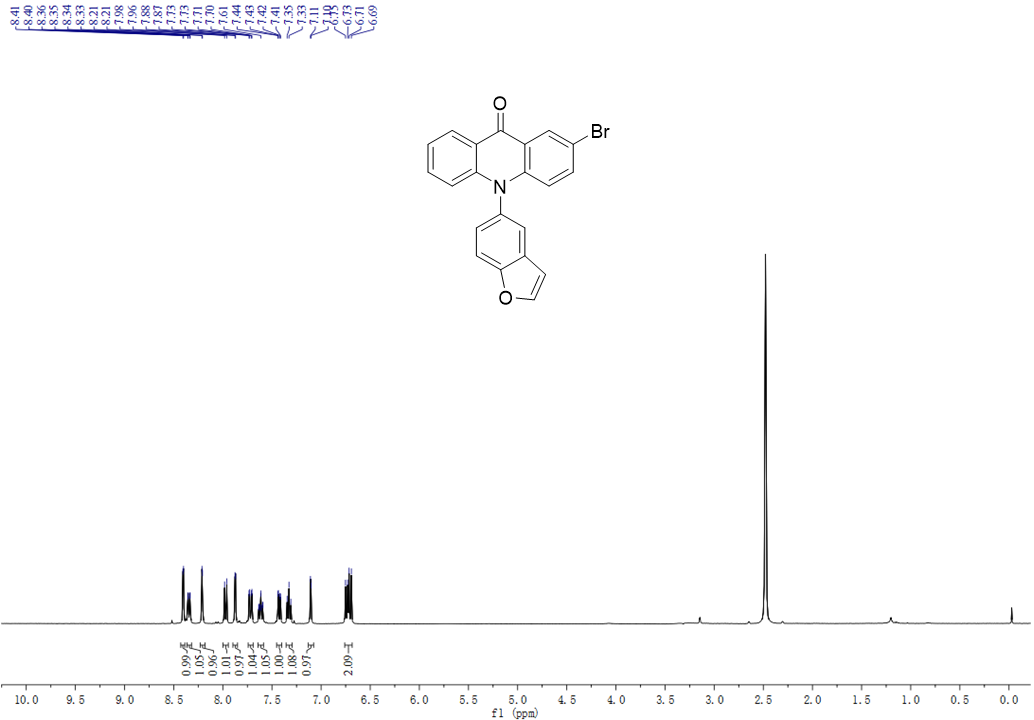


^13^C NMR spectrum of **3a** (101 MHz, DMSO)





^1^H NMR spectrum of **3b** (400 MHz, DMSO)





^13^C NMR spectrum of **3b** (101 MHz, DMSO)





^1^H NMR spectrum of **A1** (400 MHz, CDCl_3_)





^13^C NMR spectrum of **A1** (101 MHz, CDCl_3_)





HPLC profile of **A1**


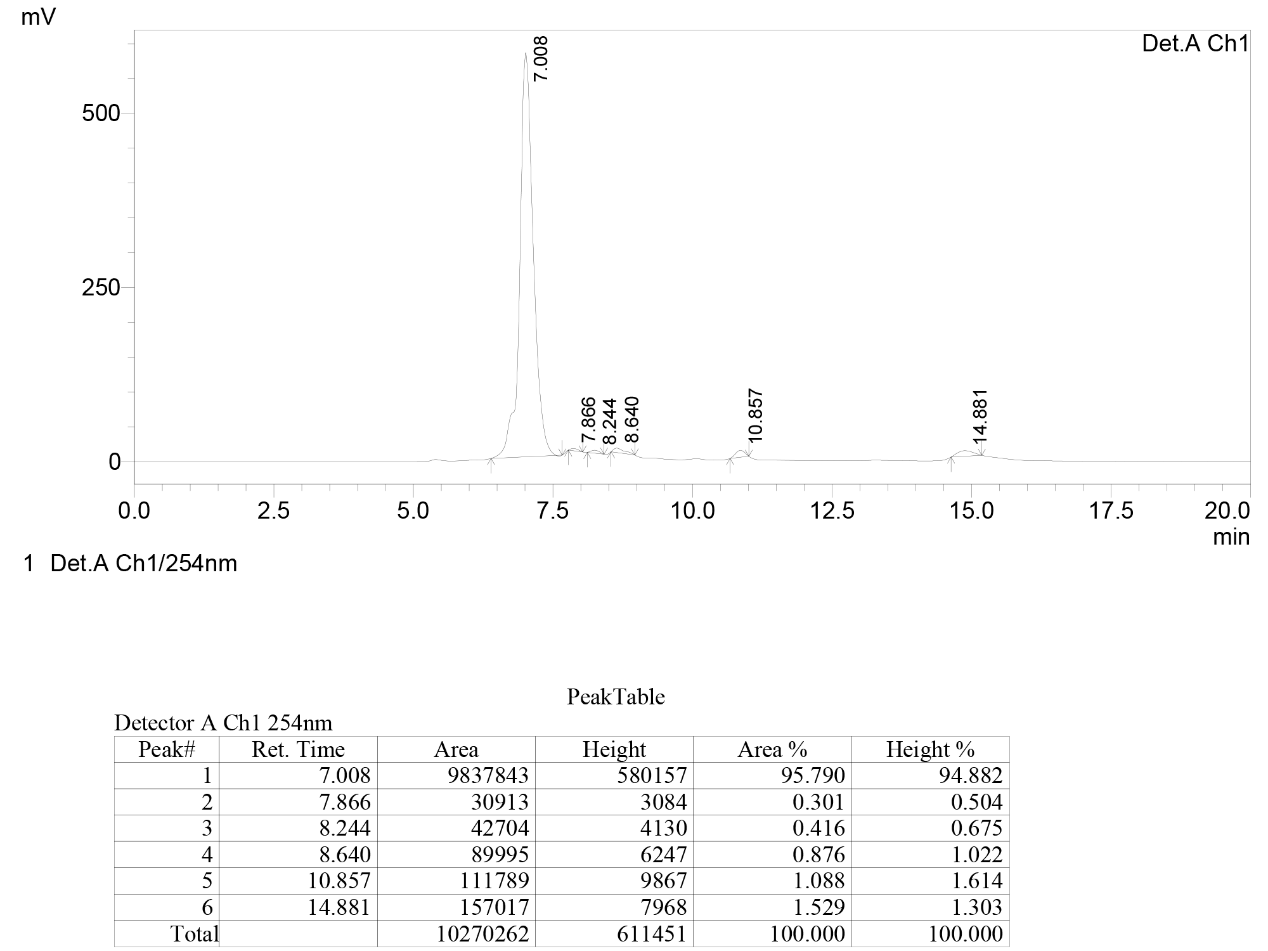


^1^H NMR spectrum of **A2** (400 MHz, CDCl_3_)





^13^C NMR spectrum of **A2** (101 MHz, CDCl_3_)





HPLC profile of **A2**


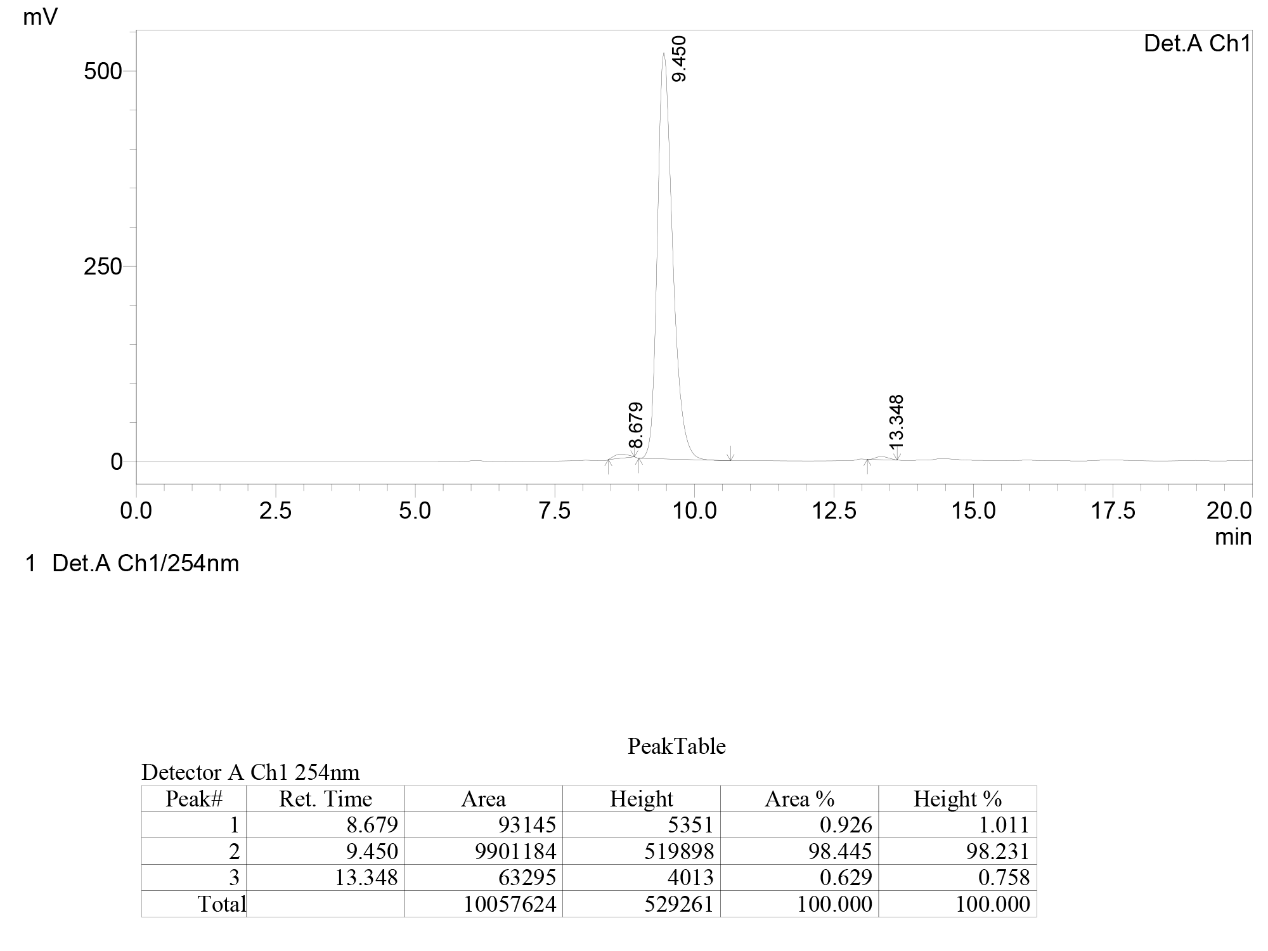


^1^H NMR spectrum of **A3** (400 MHz, CDCl_3_)





^13^C NMR spectrum of **A3** (101 MHz, CDCl_3_)





HPLC profile of **A3**


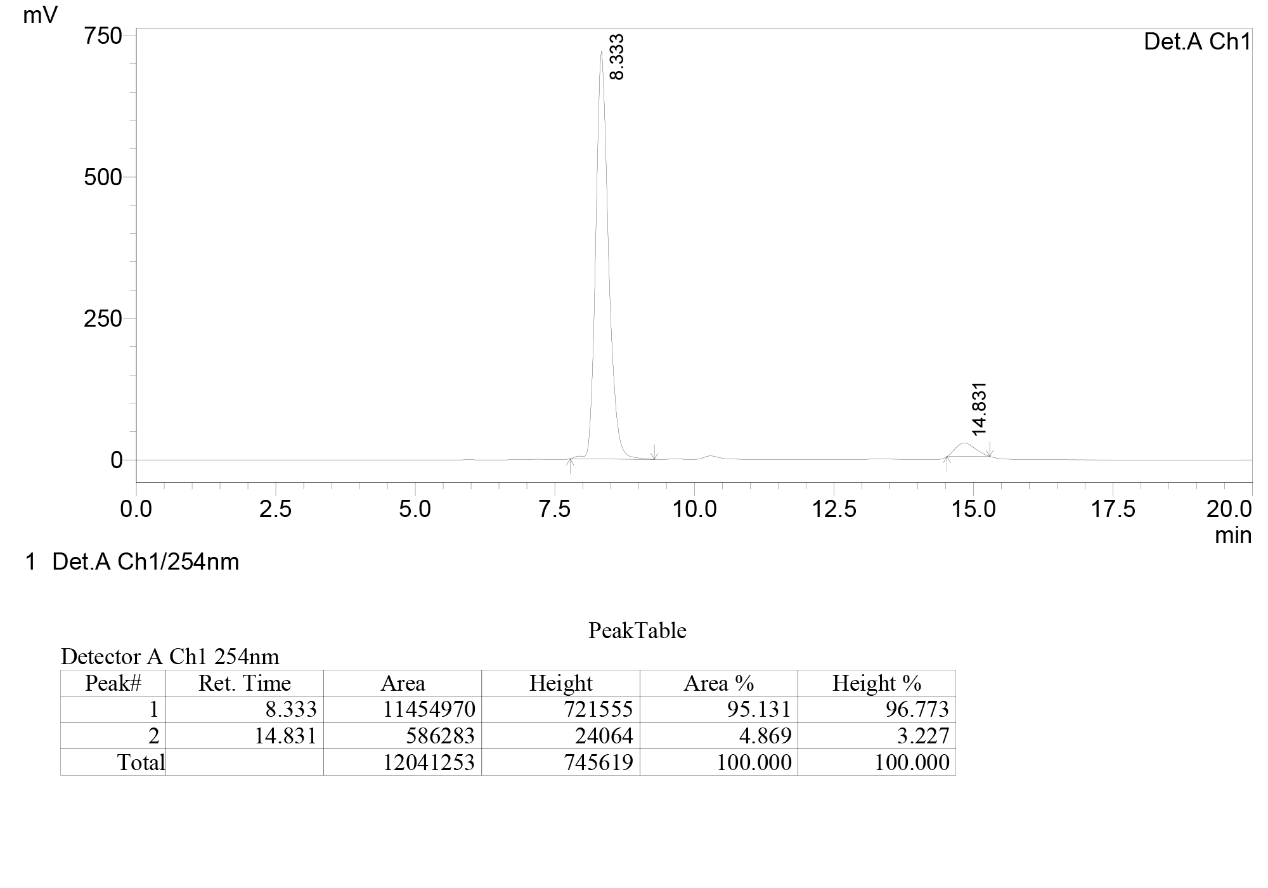


^1^H NMR spectrum of **A4** (400 MHz, CDCl_3_)





^13^C NMR spectrum of **A4** (101 MHz, CDCl_3_)





HPLC profile of **A4**


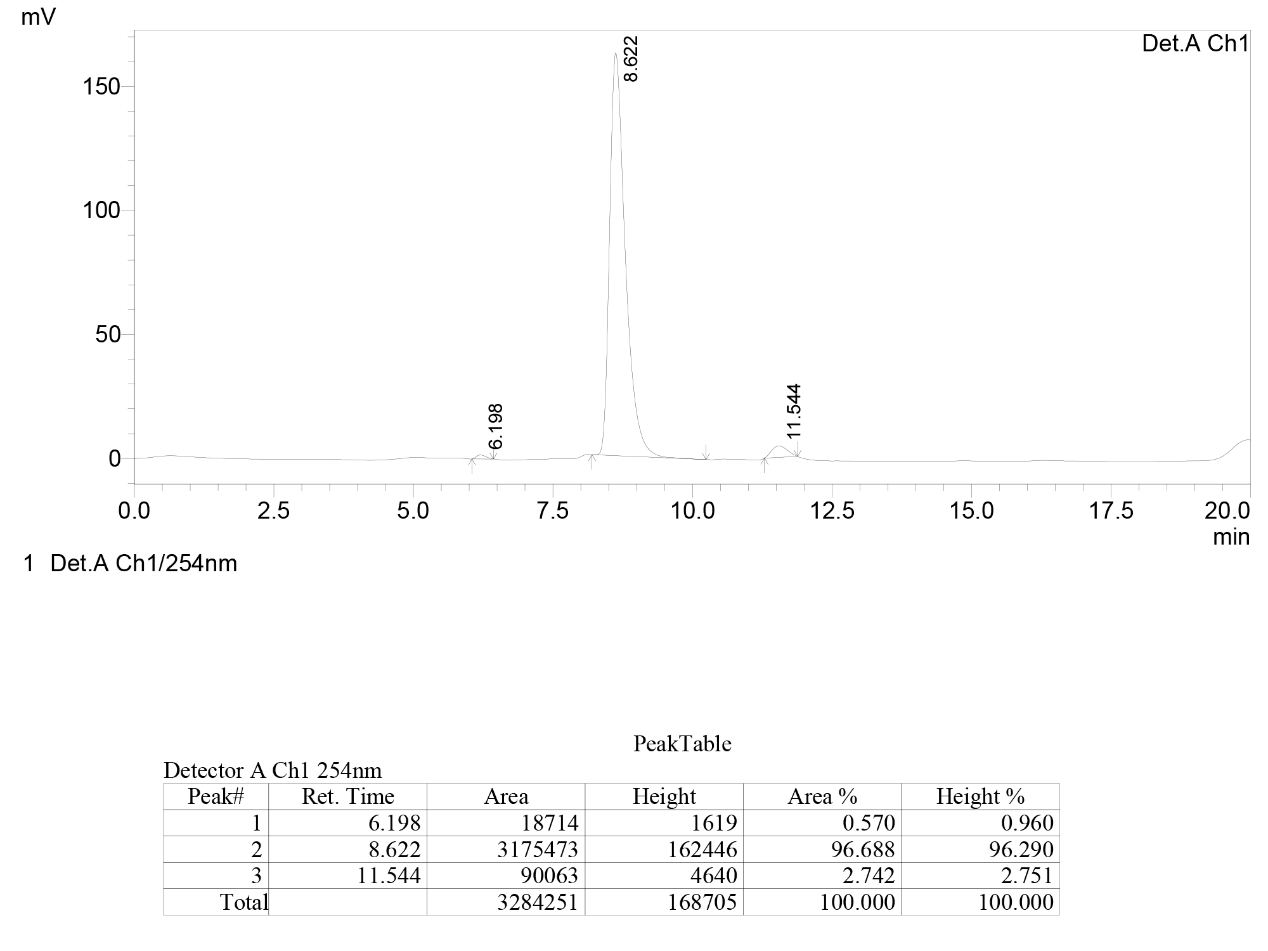


^1^H NMR spectrum of **A5** (400 MHz, CDCl_3_)





^13^C NMR spectrum of **A5** (101 MHz, CDCl_3_)





HPLC profile of **A5**


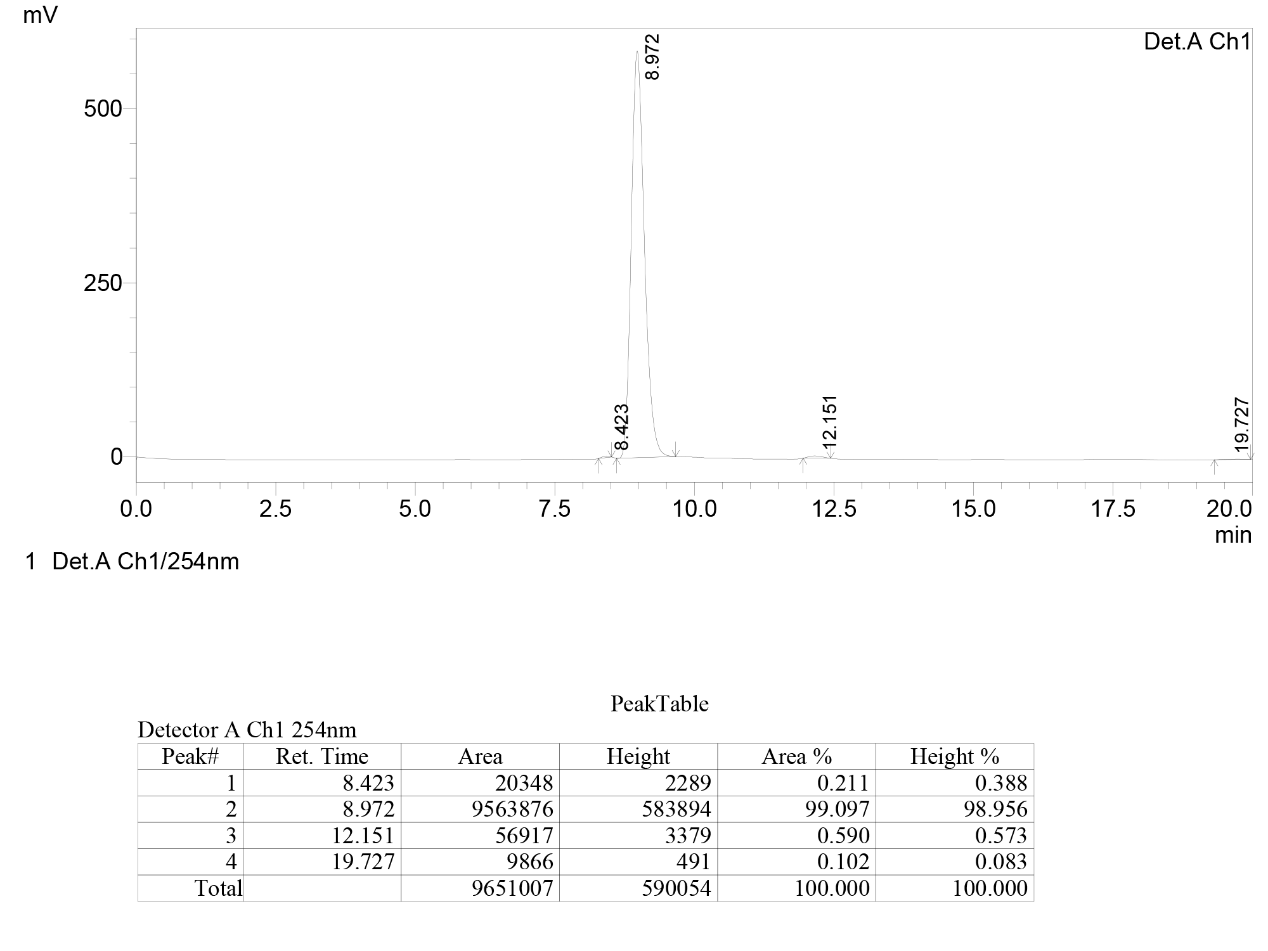


^1^H NMR spectrum of **A6** (400 MHz, CDCl_3_)





^13^C NMR spectrum of **A6** (101 MHz, CDCl_3_)





HPLC profile of **A6**


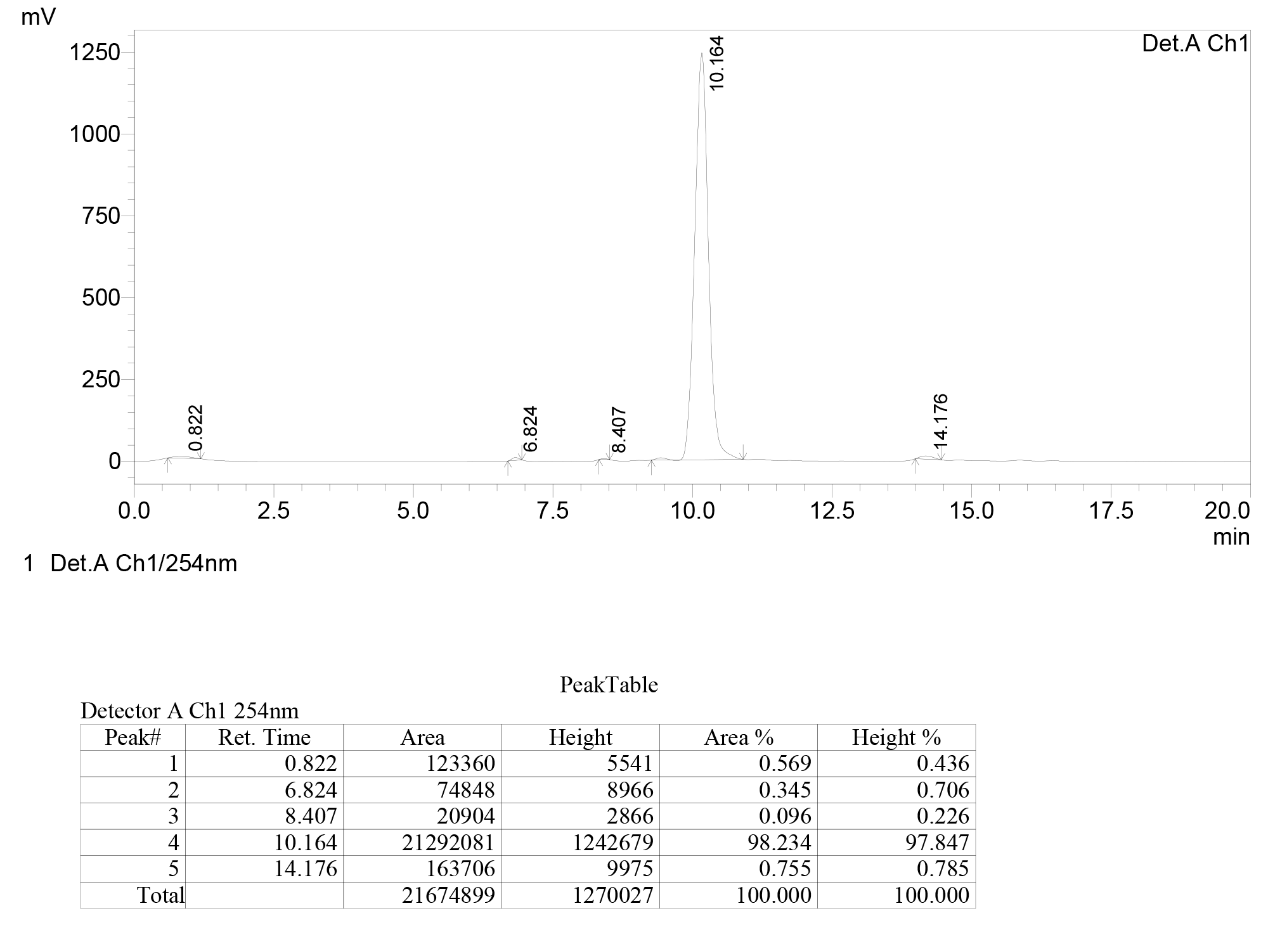


^1^H NMR spectrum of **A7** (400 MHz, CDCl_3_)





^13^C NMR spectrum of **A7** (101 MHz, CDCl_3_)





HPLC profile of **A7**


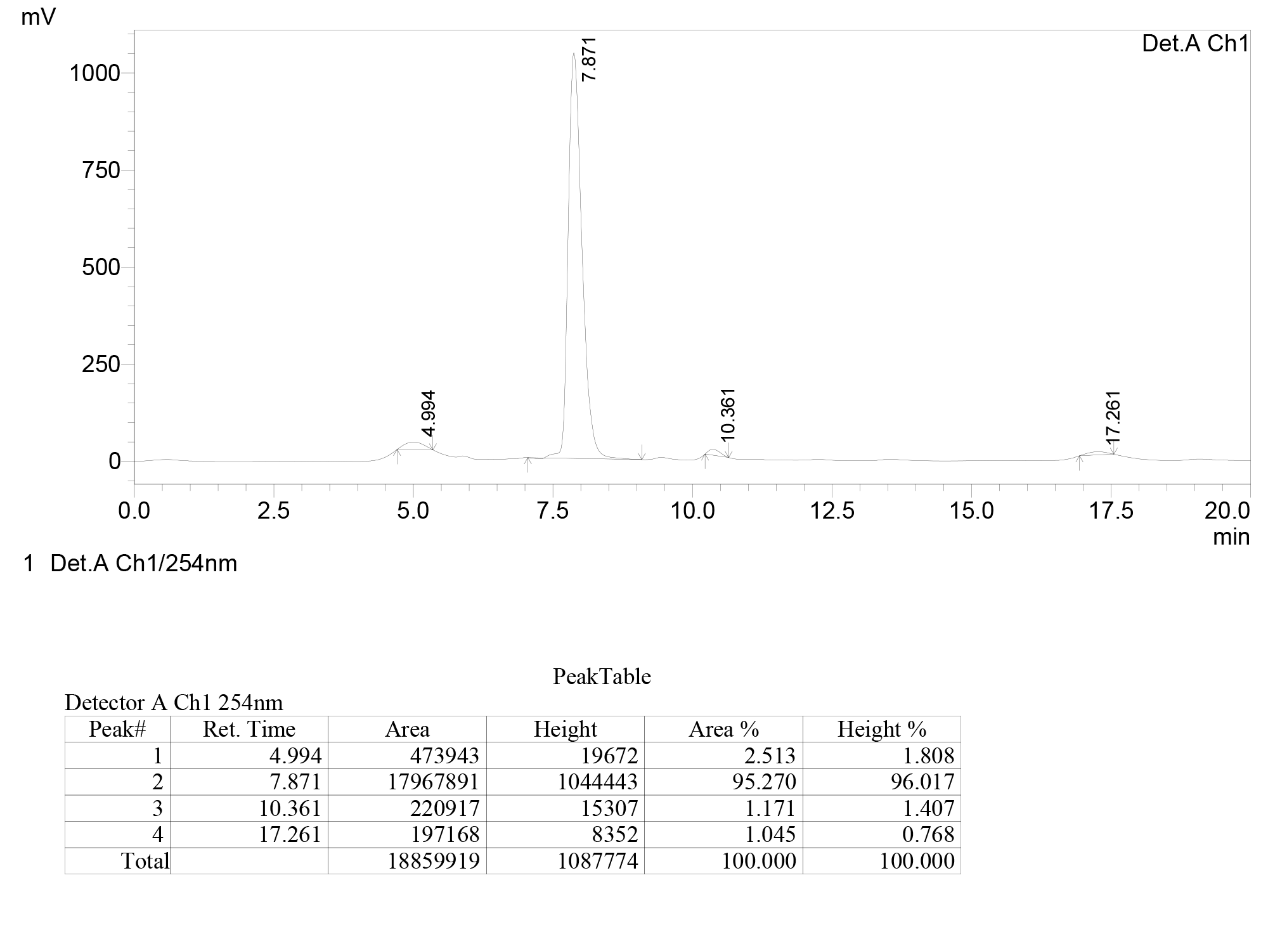


^1^H NMR spectrum of **A8** (400 MHz, CDCl_3_)





^13^C NMR spectrum of **A8** (101 MHz, CDCl_3_)





HPLC profile of **A8**


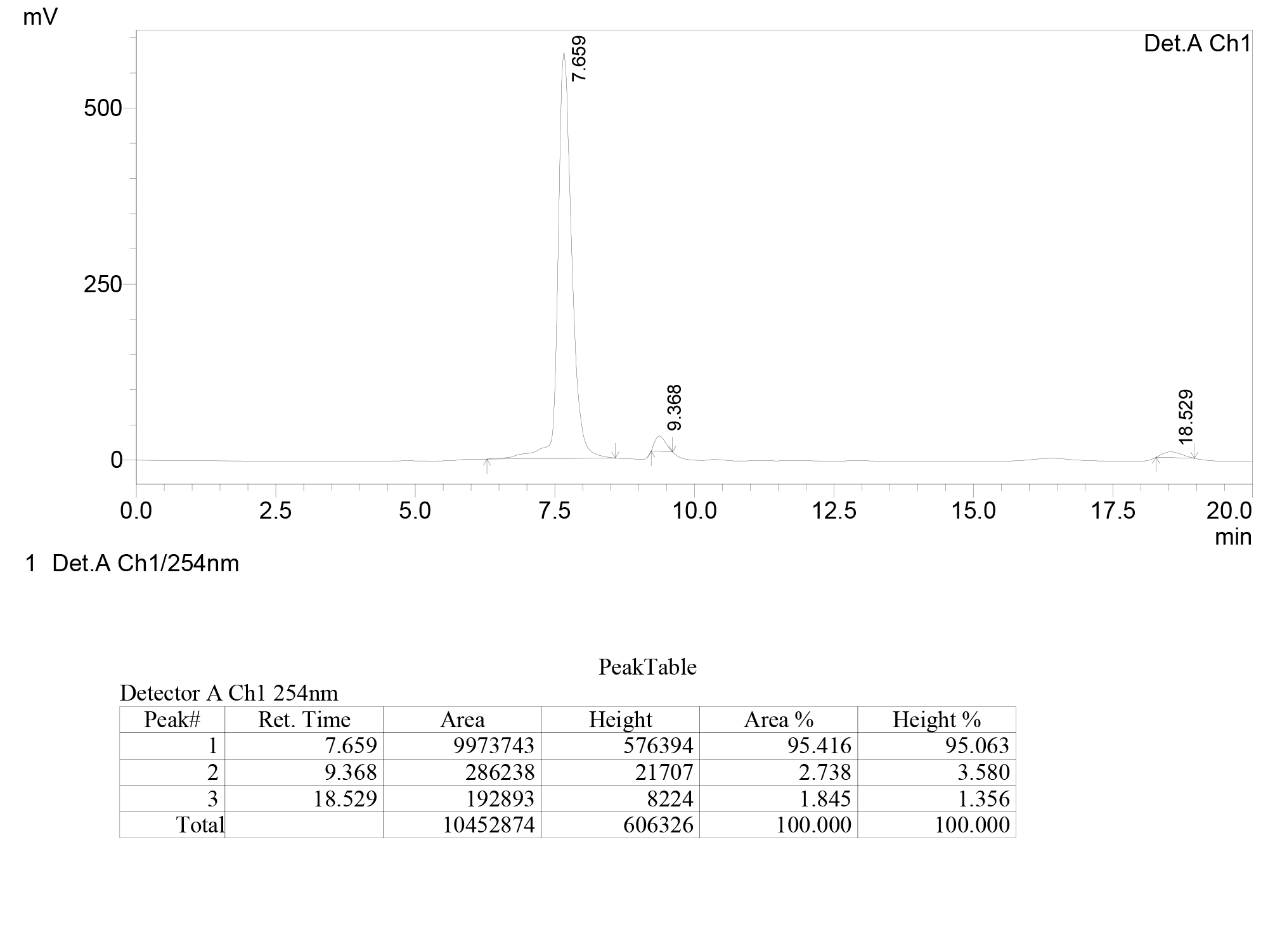


^1^H NMR spectrum of **A9** (400 MHz, CDCl_3_)





^13^C NMR spectrum of **A9** (101 MHz, CDCl_3_)





HPLC profile of **A9**


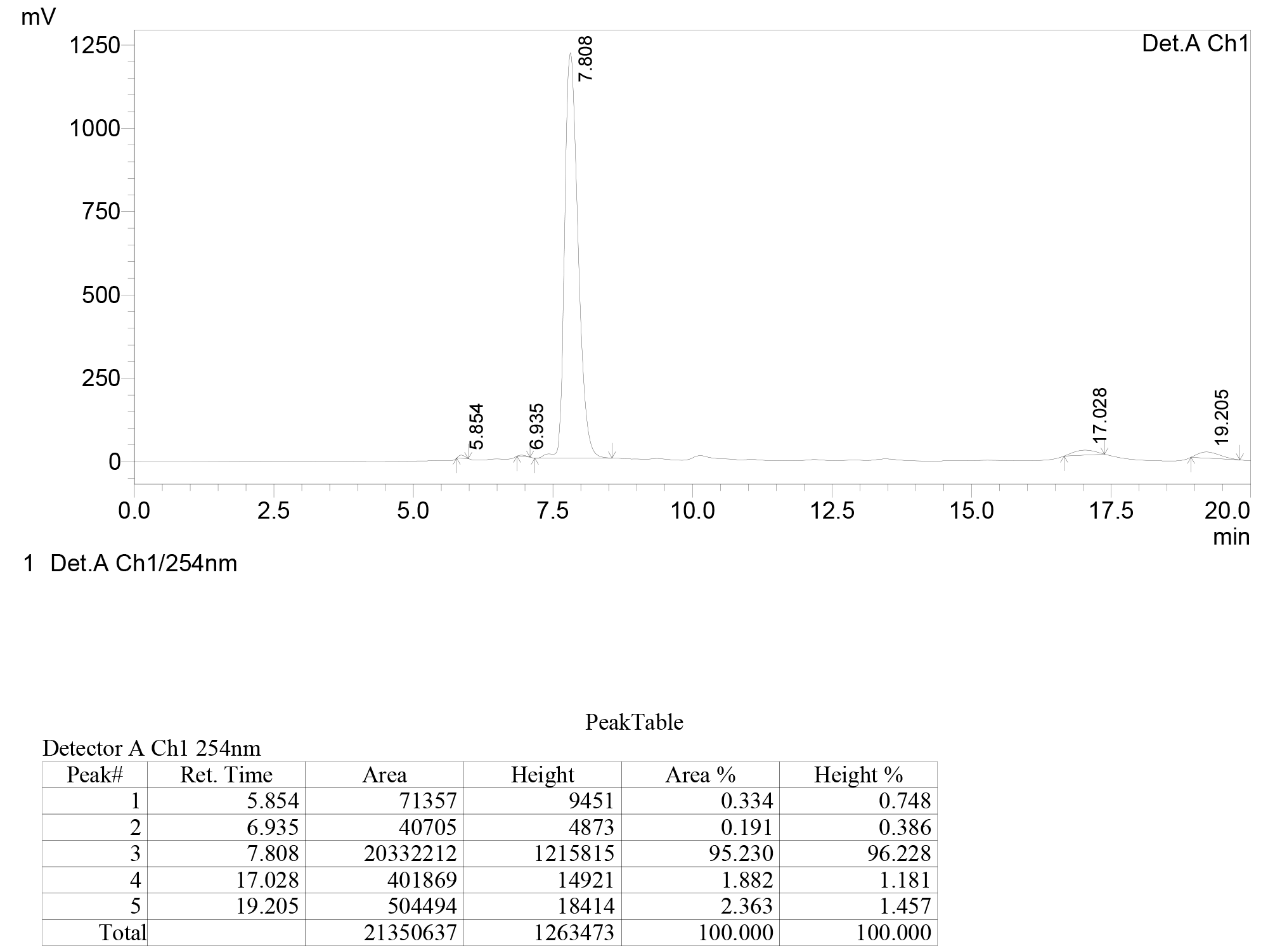


^1^H NMR spectrum of **A10** (400 MHz, CDCl_3_)





^13^C NMR spectrum of **A10** (101 MHz, CDCl_3_)





HPLC profile of **A10**


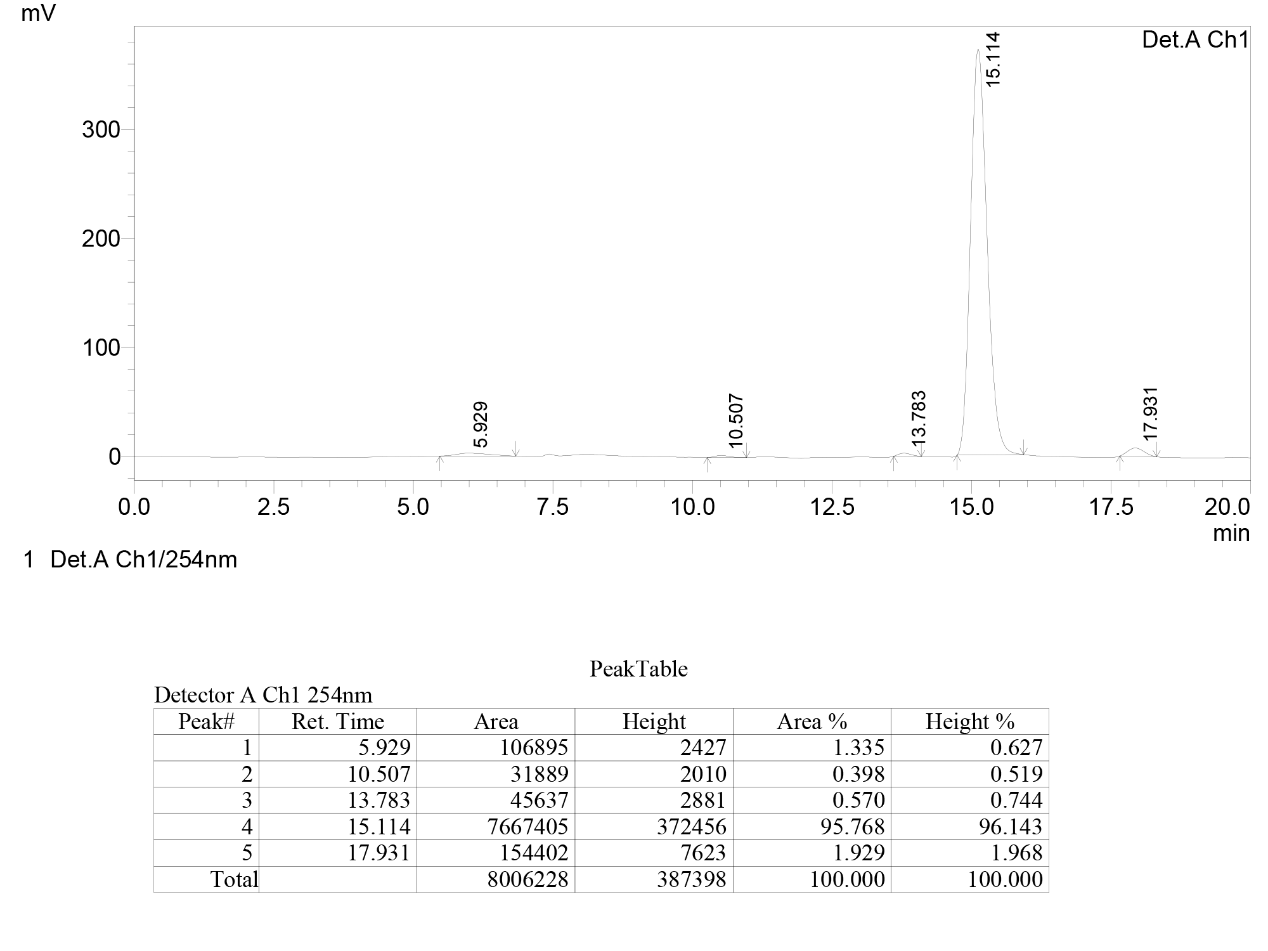


^1^H NMR spectrum of **A11** (400 MHz, CDCl_3_)





^13^C NMR spectrum of **A11** (101 MHz, CDCl_3_)





HPLC profile of **A11**


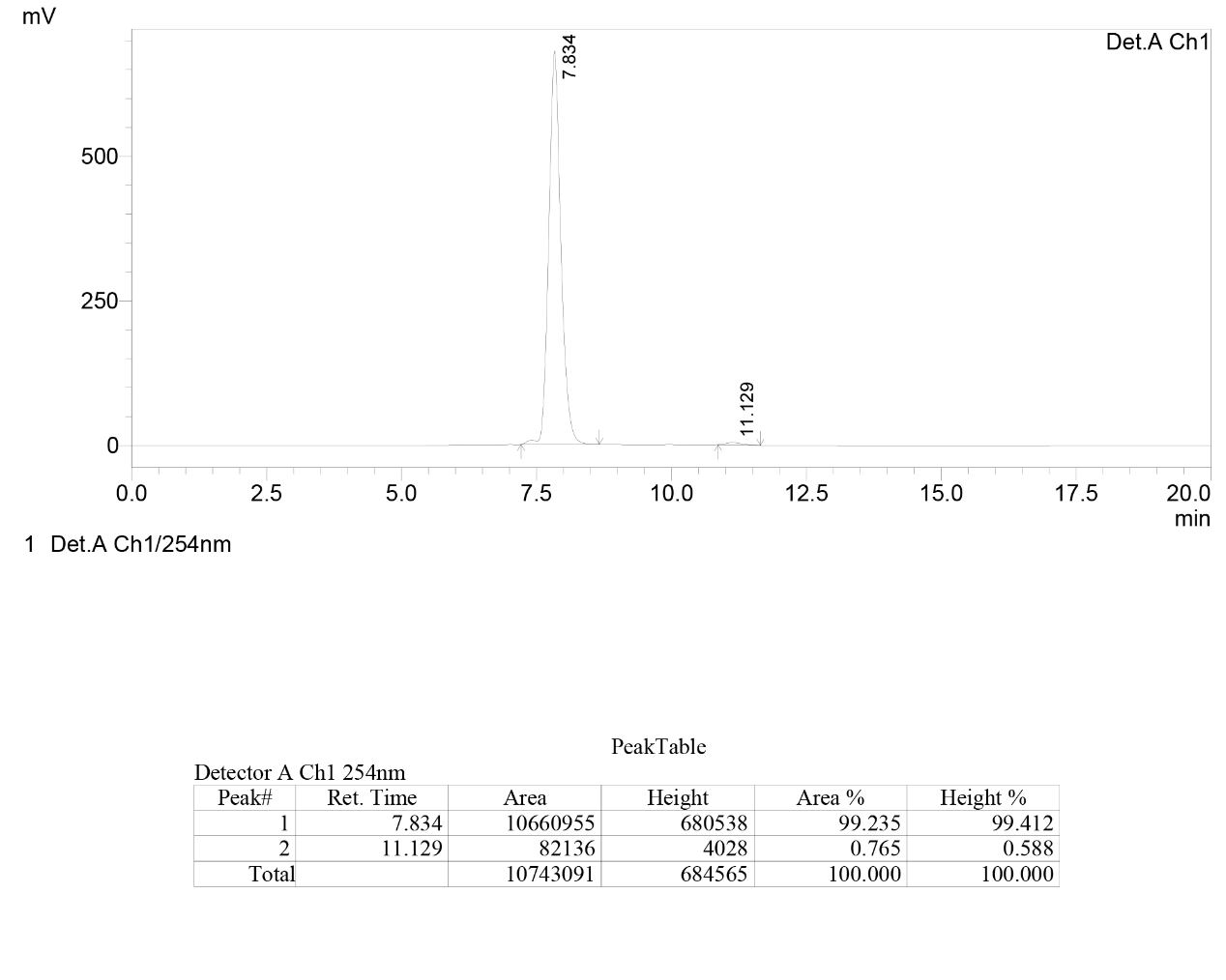


^1^H NMR spectrum of **A12** (400 MHz, CDCl_3_)





^13^C NMR spectrum of **A12** (101 MHz, CDCl_3_)





HPLC profile of **A12**


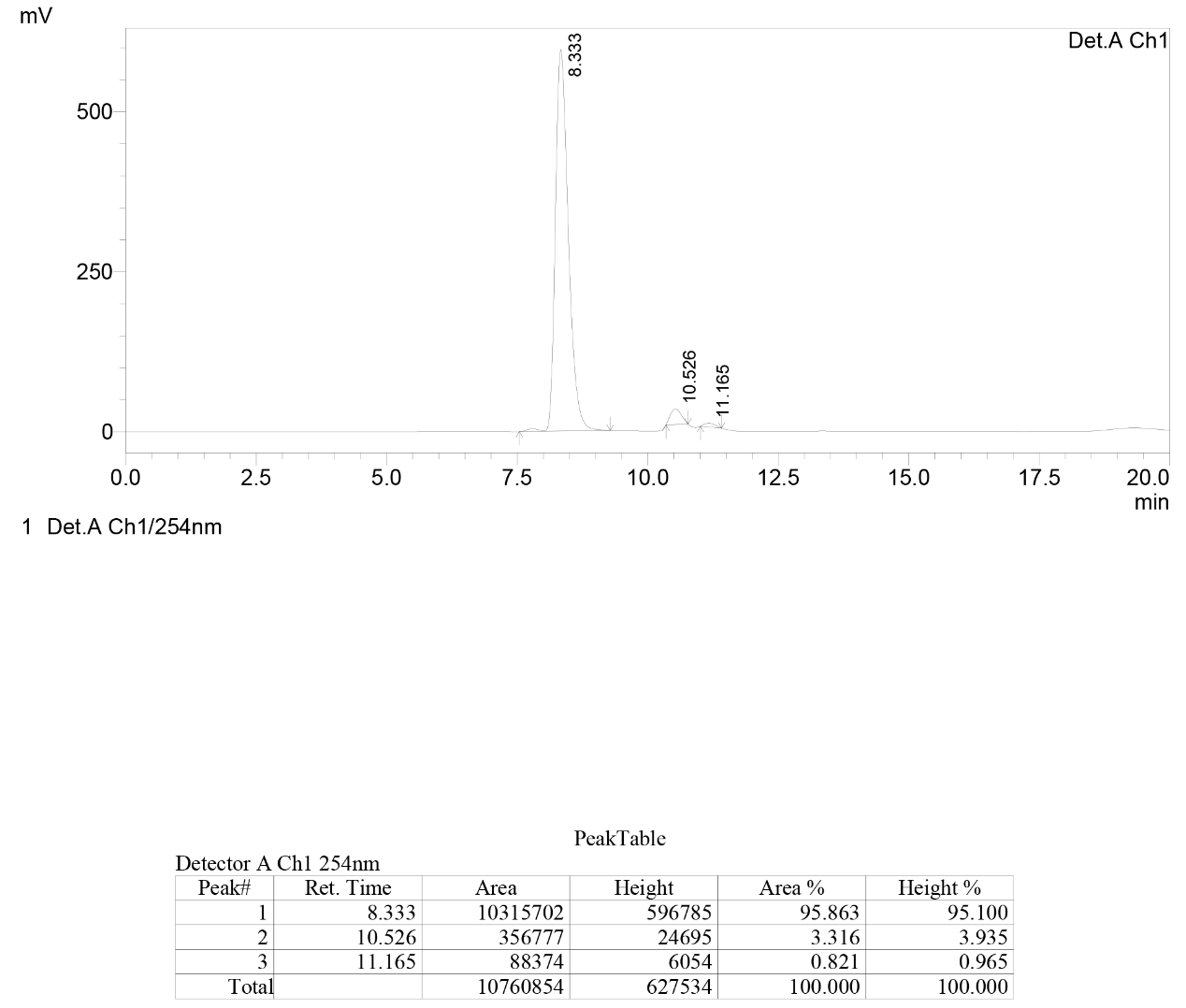


^1^H NMR spectrum of **A13** (400 MHz, CDCl_3_)





^13^C NMR spectrum of **A13** (101 MHz, CDCl_3_)





HPLC profile of **A13**


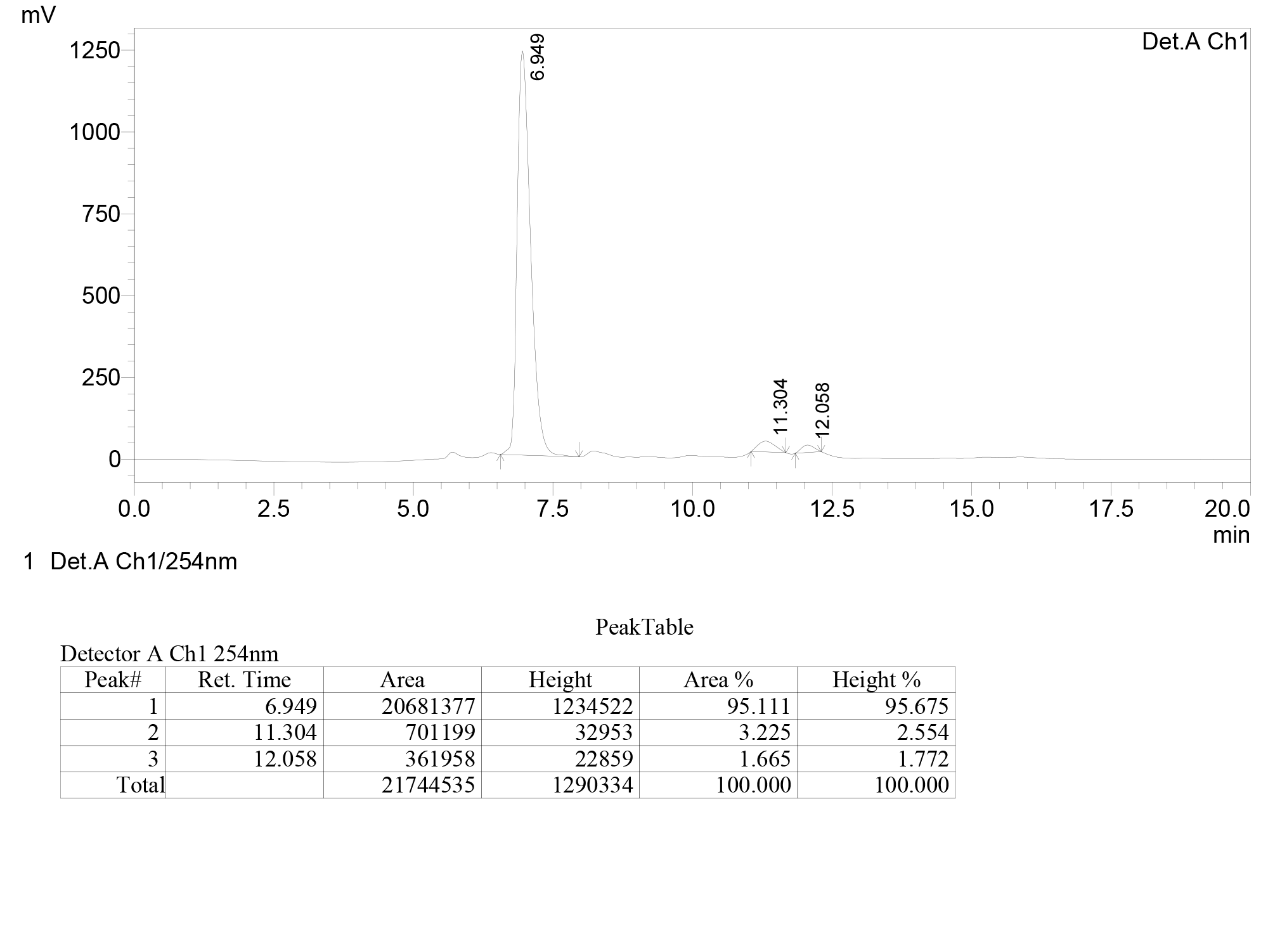


^1^H NMR spectrum of **A14** (400 MHz, CDCl_3_)





^13^C NMR spectrum of **A14** (101 MHz, CDCl_3_)





HPLC profile of **A14**


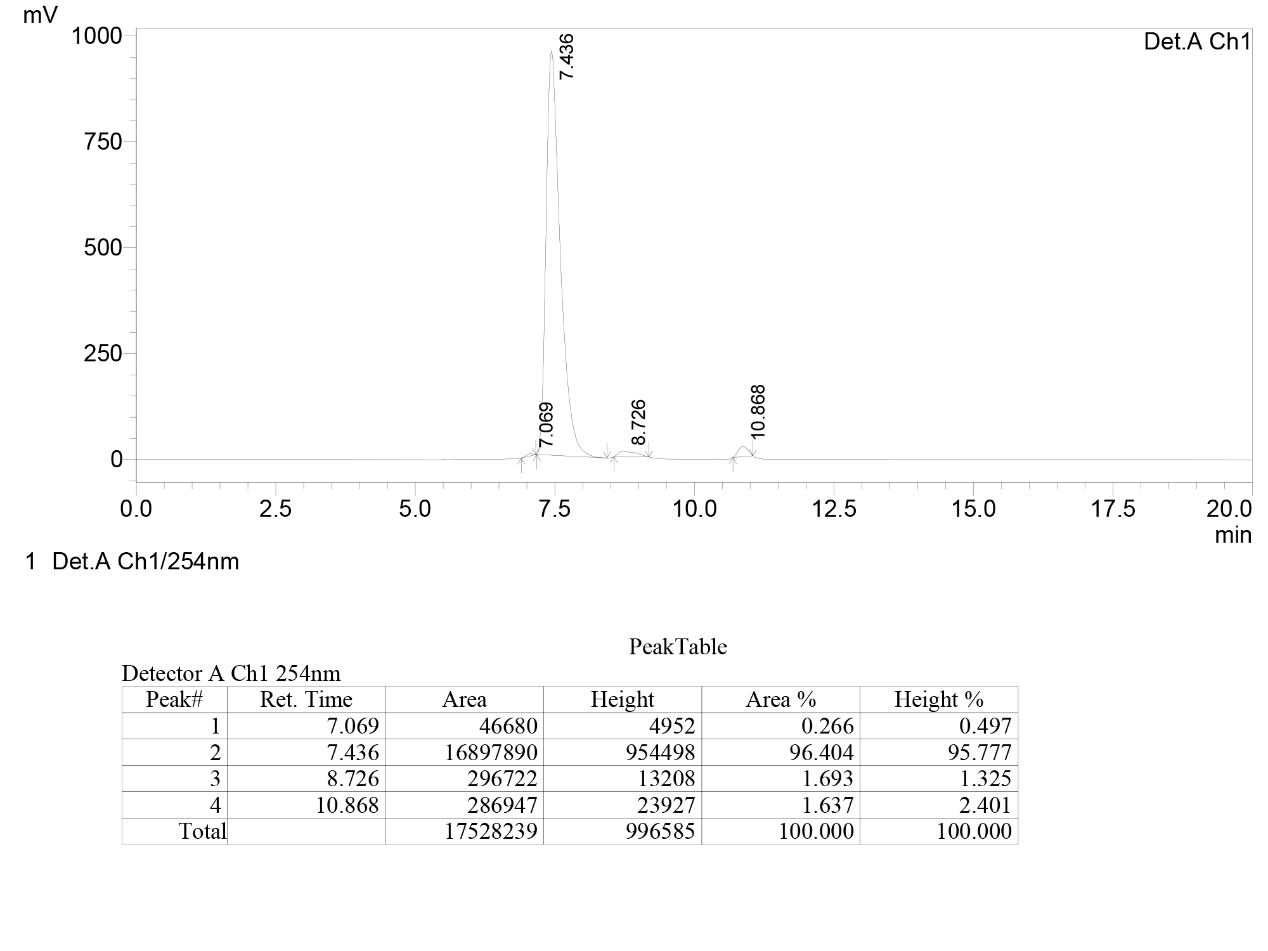


^1^H NMR spectrum of **A15** (400 MHz, CDCl_3_)





^13^C NMR spectrum of **A15** (101 MHz, CDCl_3_)





HPLC profile of **A15**


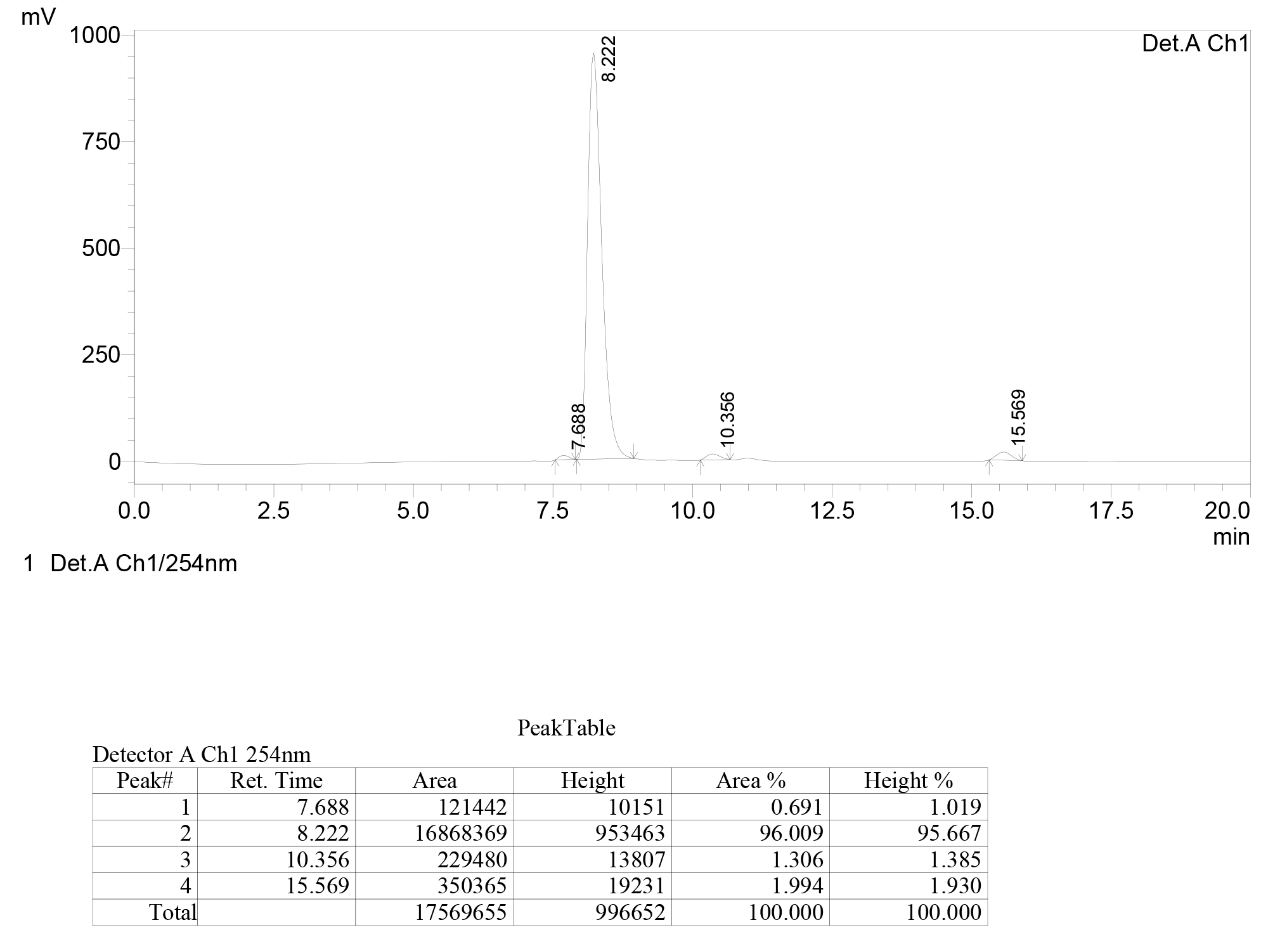


^1^H NMR spectrum of **A16** (400 MHz, CDCl_3_)
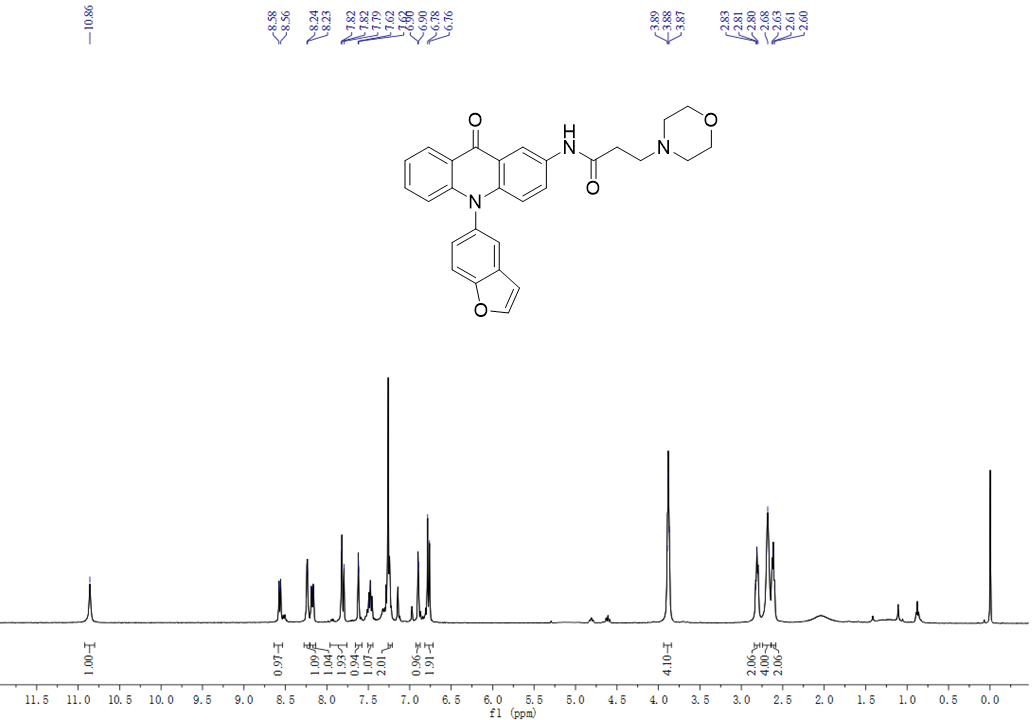


^13^C NMR spectrum of **A16** (101 MHz, CDCl_3_)



HPLC profile of **A16**


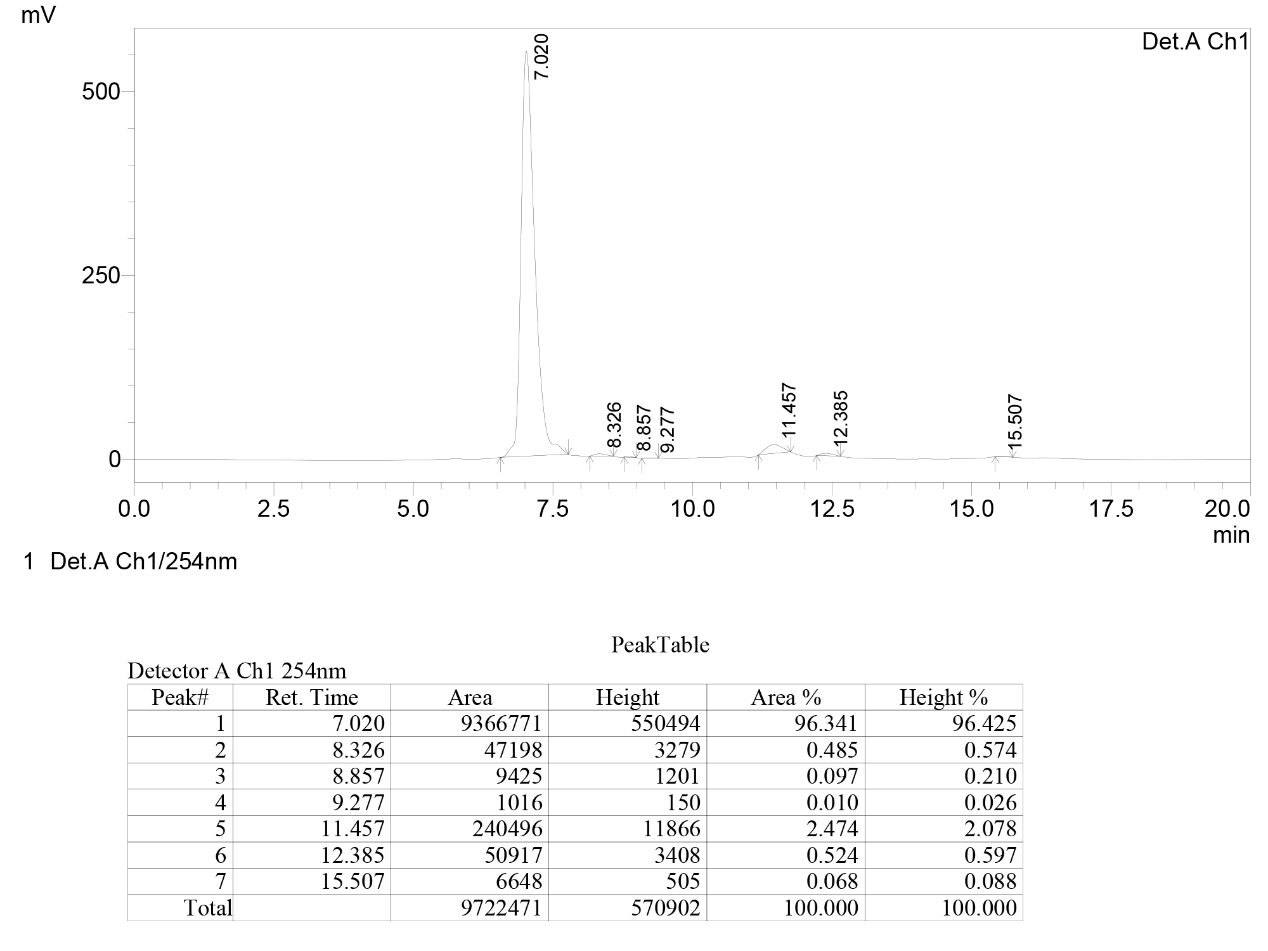


^1^H NMR spectrum of **B1** (400 MHz, CDCl_3_)





^13^C NMR spectrum of **B1** (101 MHz, CDCl_3_)





HPLC profile of **B1**


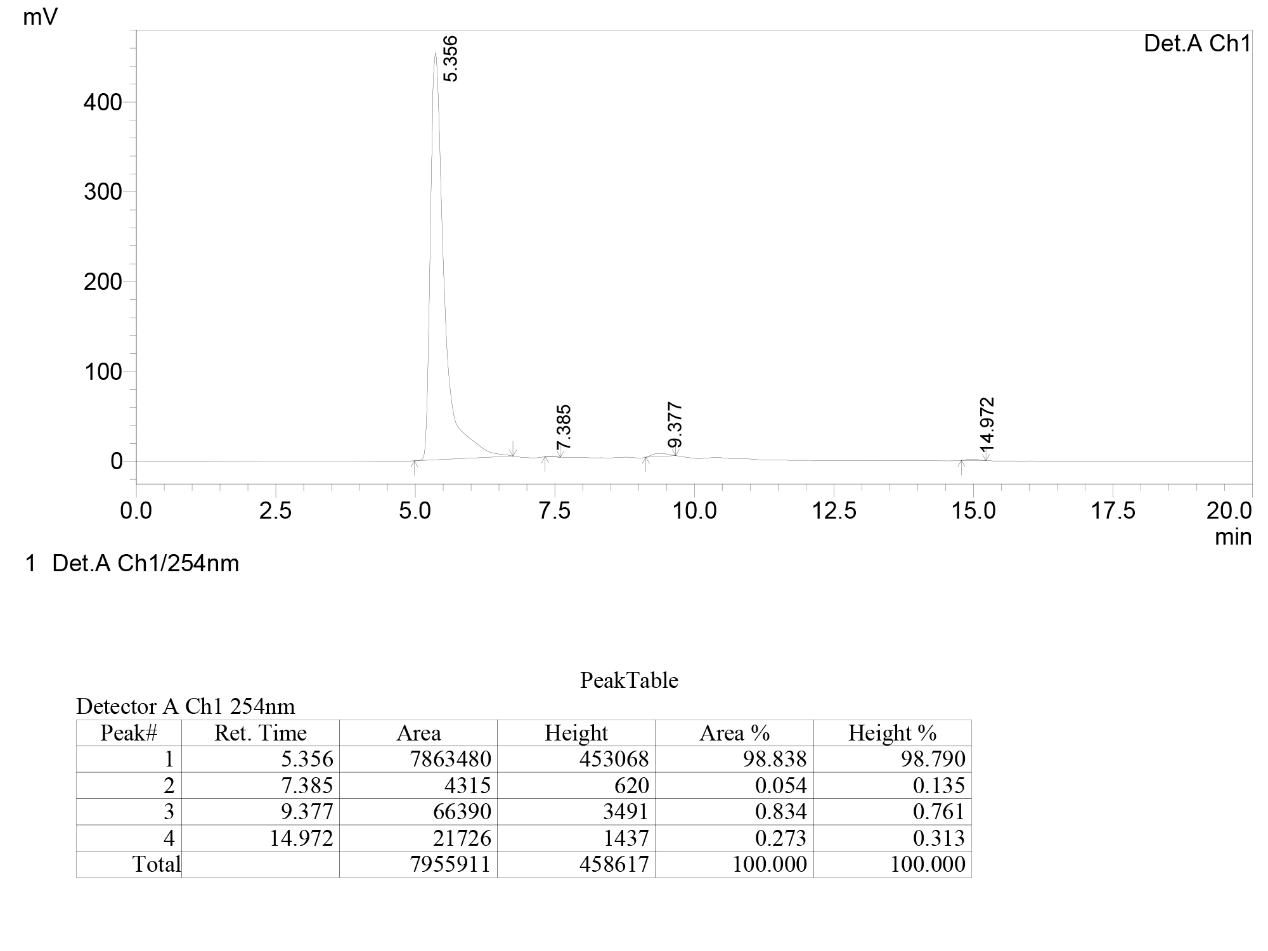


^1^H NMR spectrum of **B2** (400 MHz, CDCl_3_)





^13^C NMR spectrum of **B2** (101 MHz, CDCl_3_)





HPLC profile of **B2**


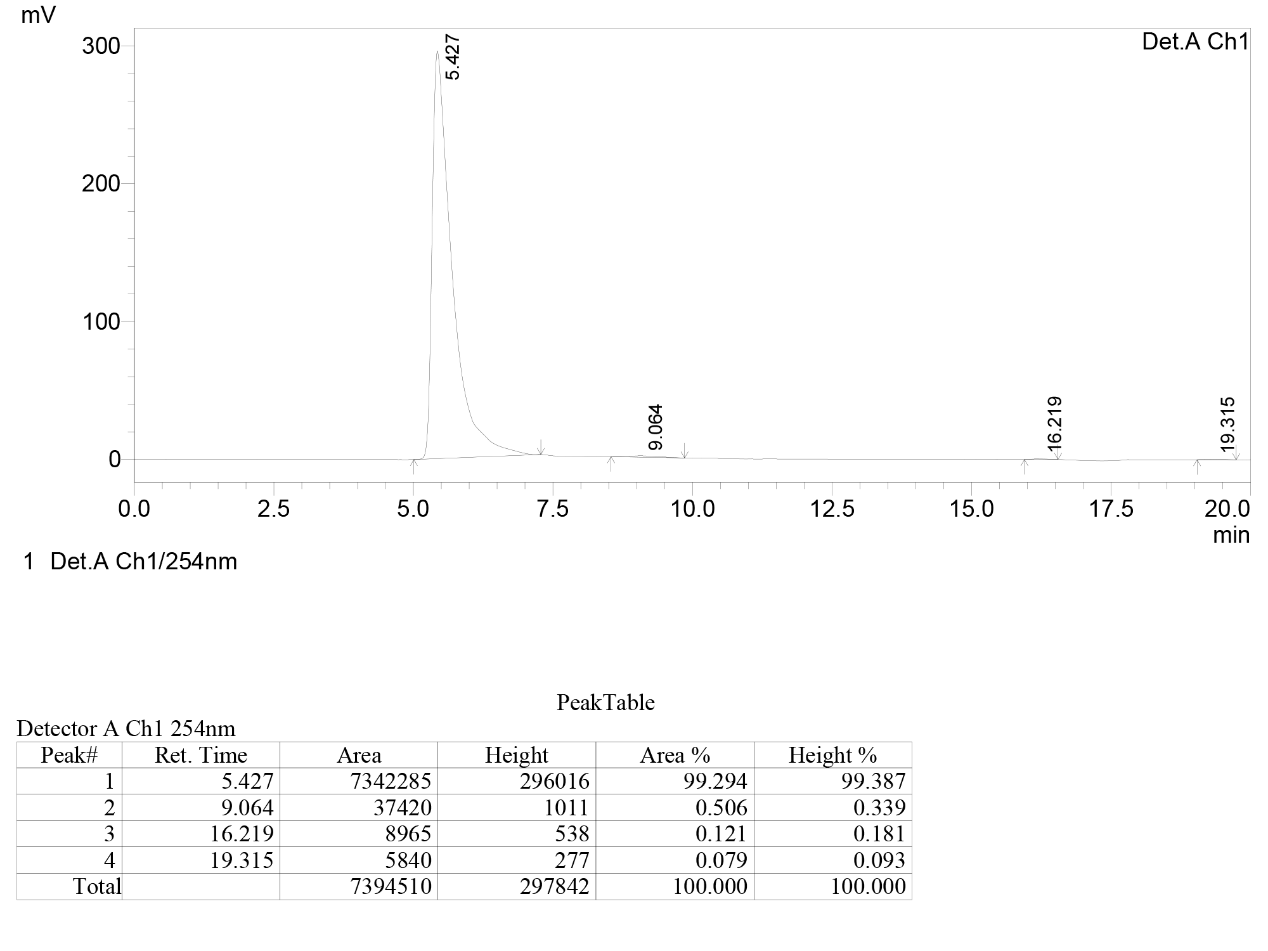


^1^H NMR spectrum of **B3** (400 MHz, CDCl_3_)





^13^C NMR spectrum of **B3** (101 MHz, CDCl_3_)





HPLC profile of **B3**


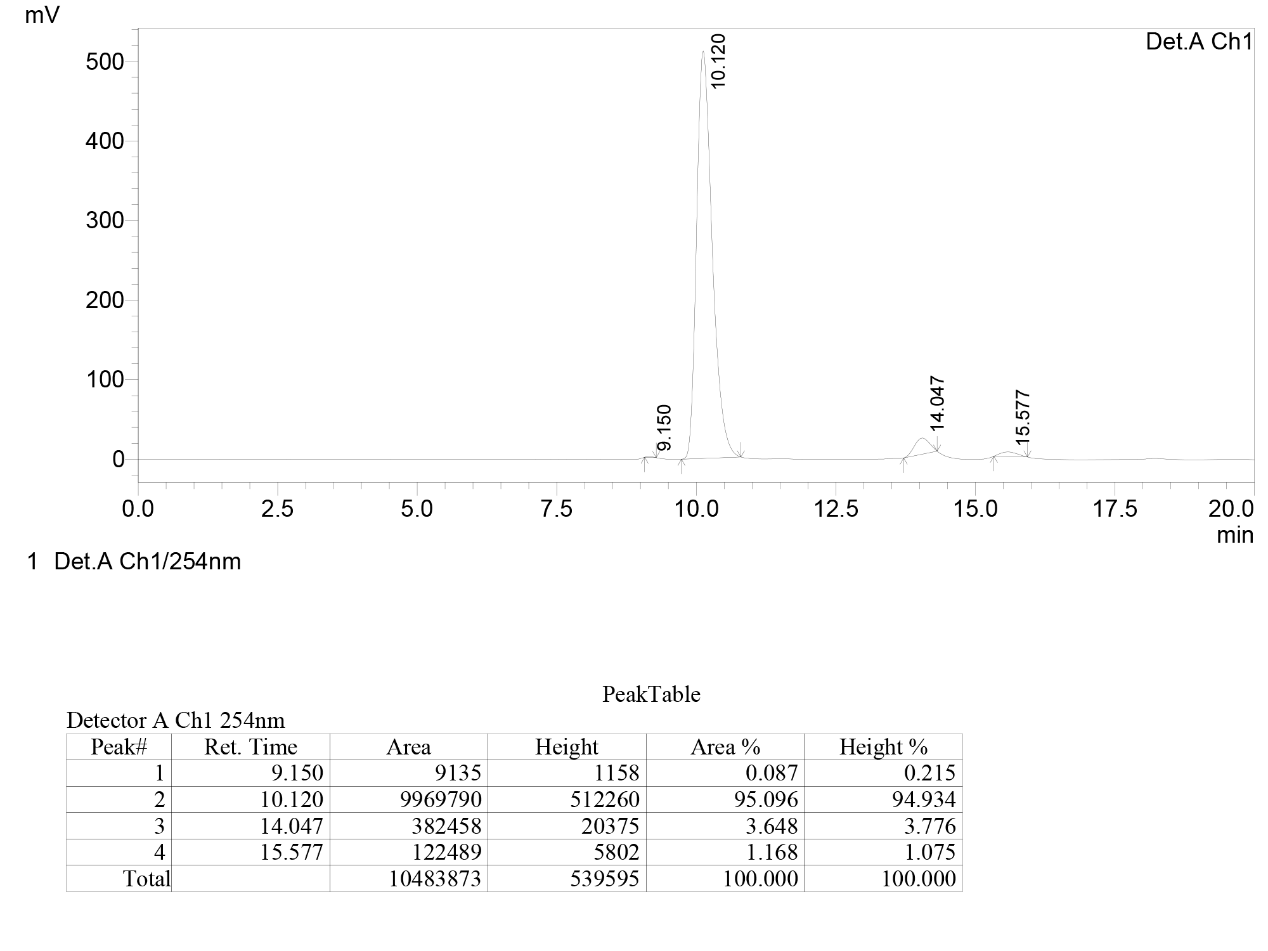


^1^H NMR spectrum of **B4** (400 MHz, CDCl_3_)





^13^C NMR spectrum of **B4** (101 MHz, CDCl_3_)





HPLC profile of **B4**


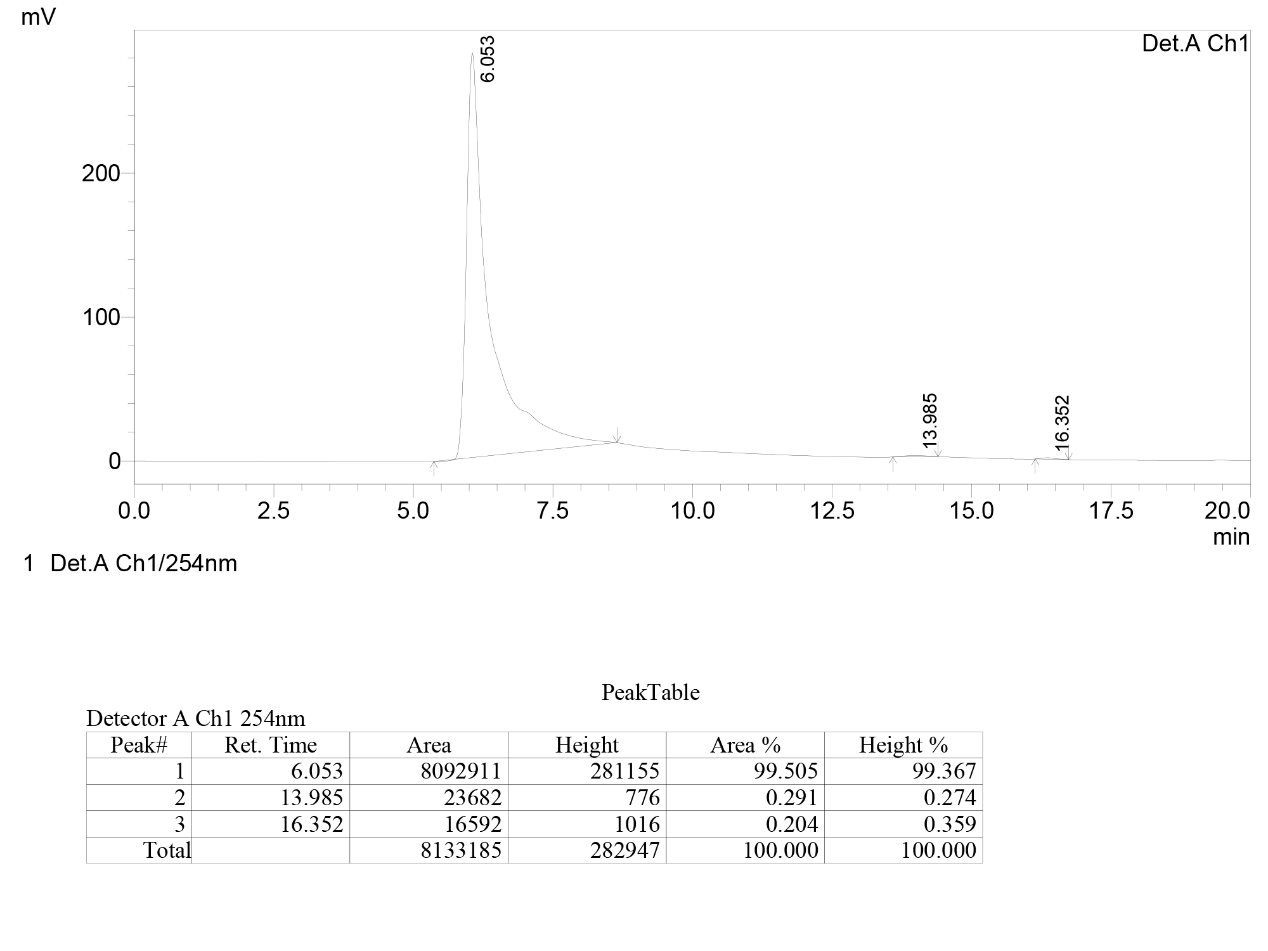


^1^H NMR spectrum of **B5** (400 MHz, CDCl_3_)





^13^C NMR spectrum of **B5** (101 MHz, CDCl_3_)





HPLC profile of **B5**


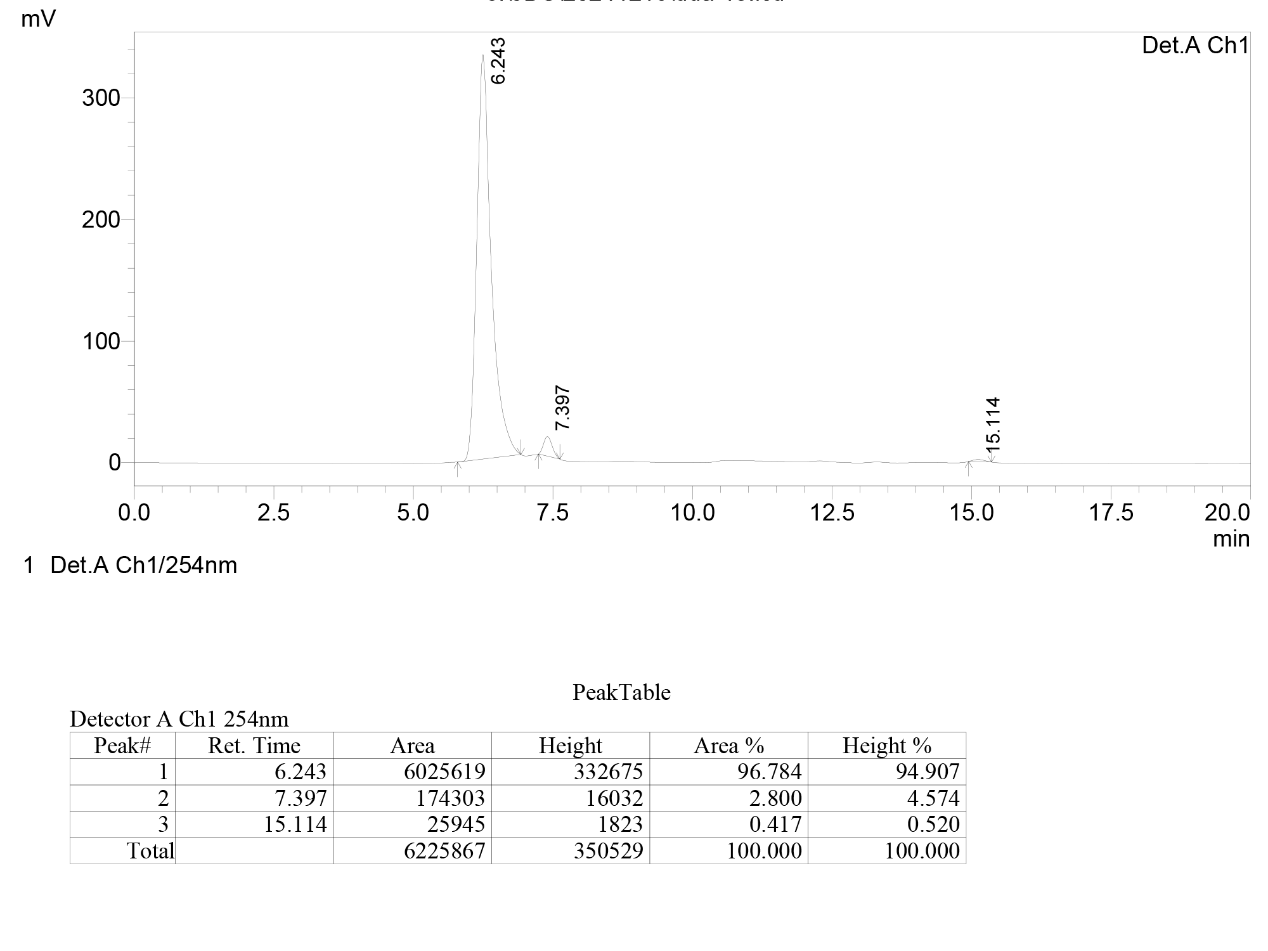


^1^H NMR spectrum of **B6** (400 MHz, CDCl_3_)





^13^C NMR spectrum of **B6** (101 MHz, CDCl_3_)





HPLC profile of **B6**


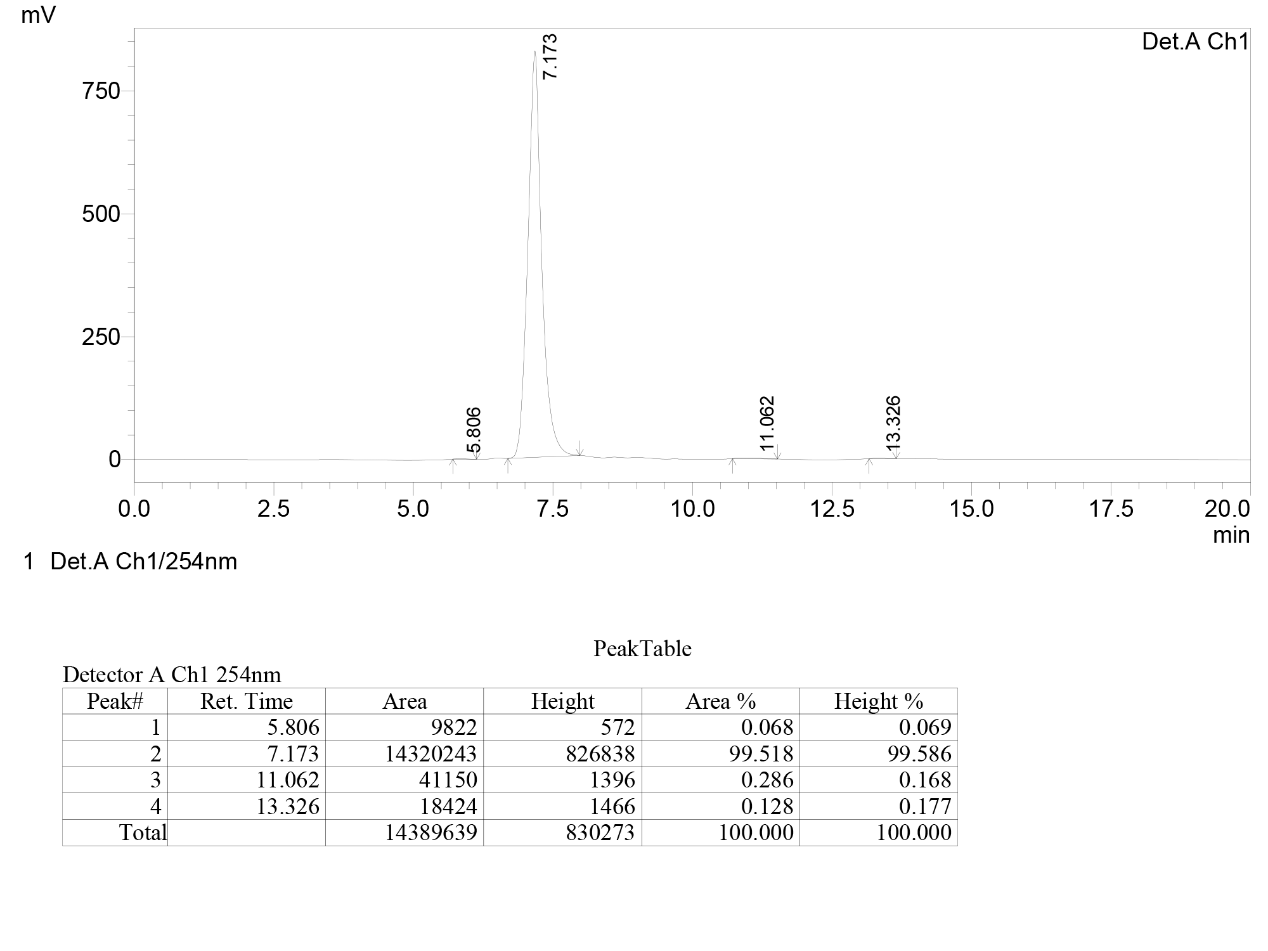


^1^H NMR spectrum of **B7** (400 MHz, CDCl_3_)





^13^C NMR spectrum of **B7** (101 MHz, CDCl_3_)





HPLC profile of **B7**


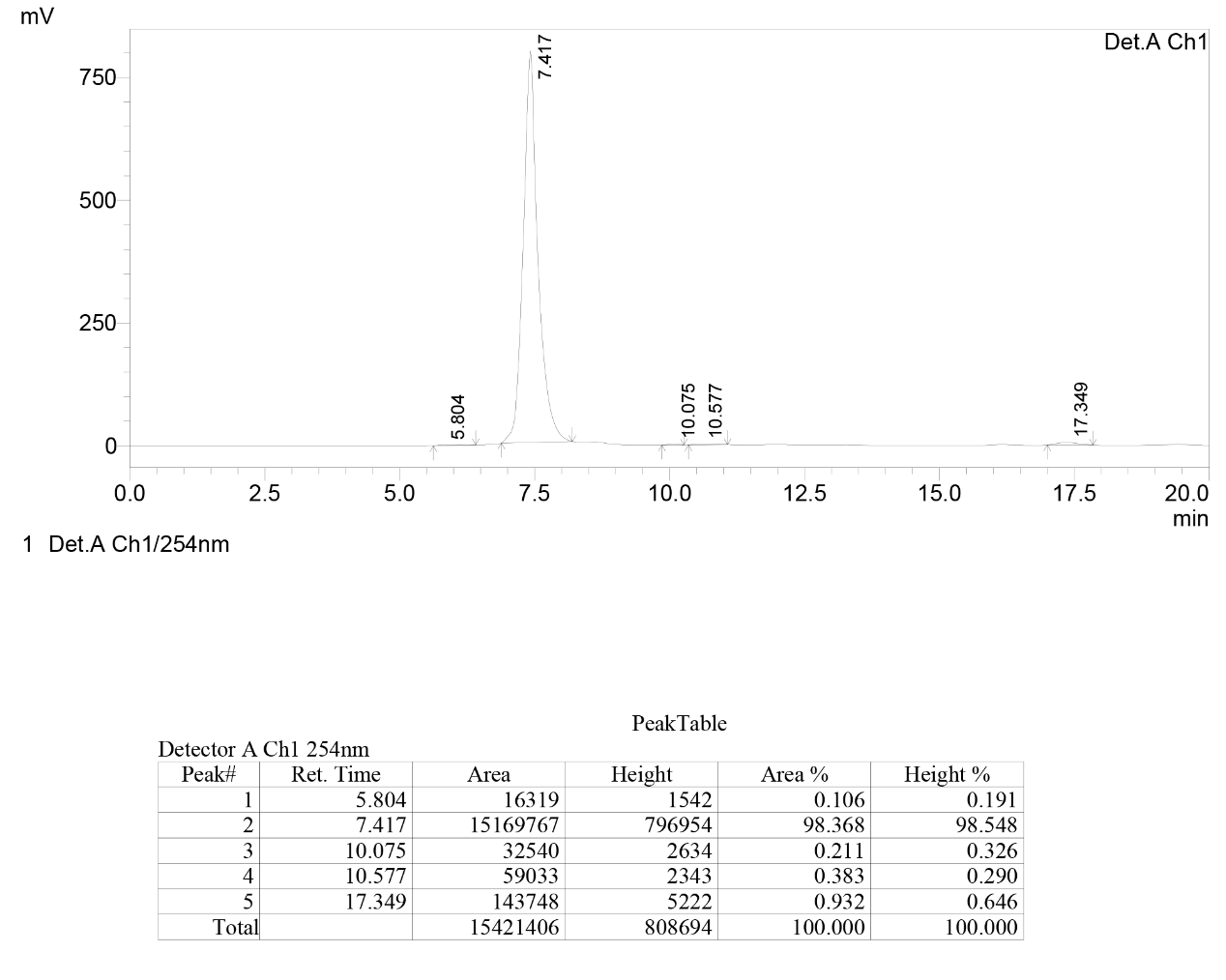


^1^H NMR spectrum of **B8** (400 MHz, CDCl_3_)





^13^C NMR spectrum of **B8** (101 MHz, CDCl_3_)





HPLC profile of **B8**


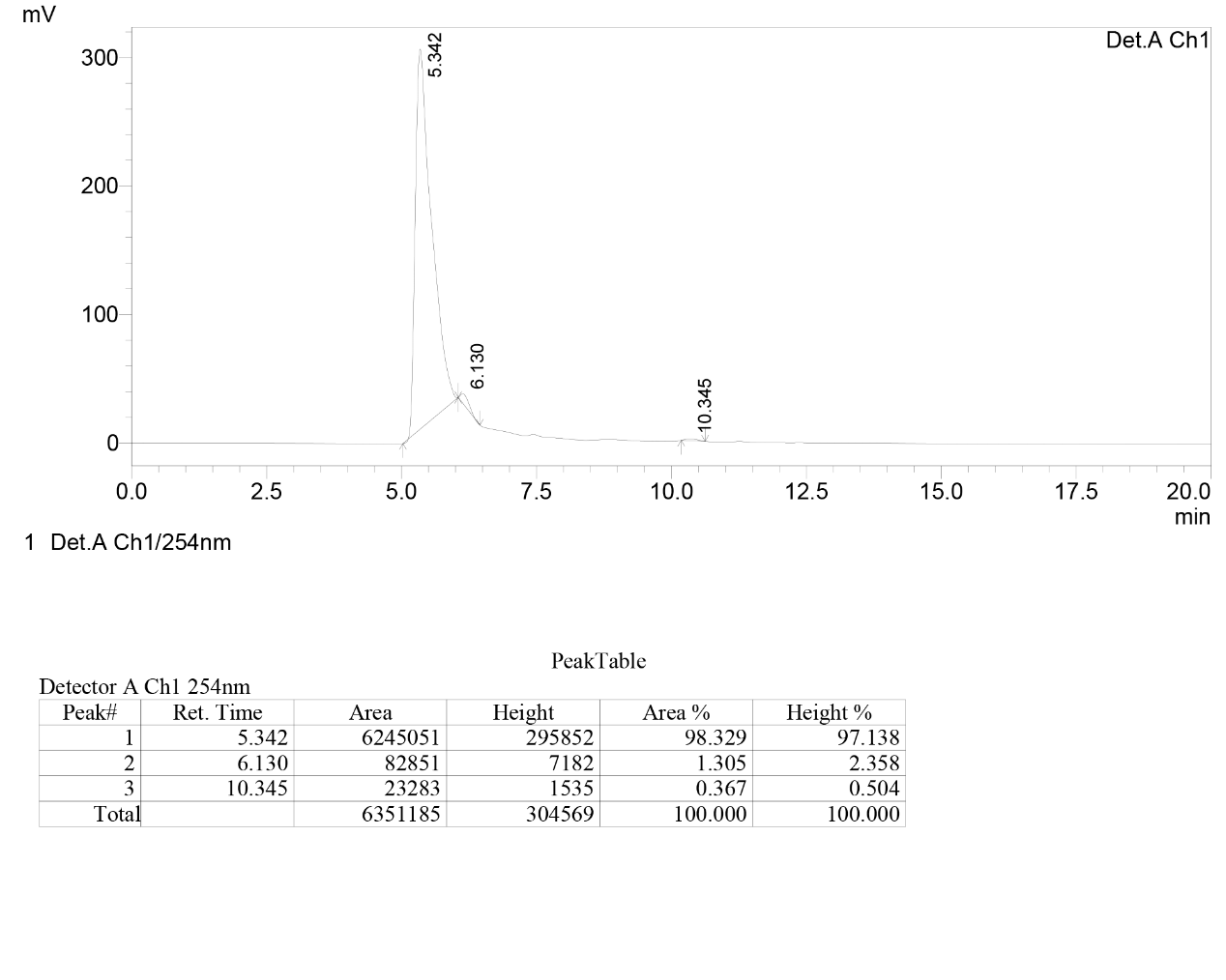


^1^H NMR spectrum of **B9** (400 MHz, CDCl_3_)





^13^C NMR spectrum of **B9** (101 MHz, CDCl_3_)





HPLC profile of **B9**


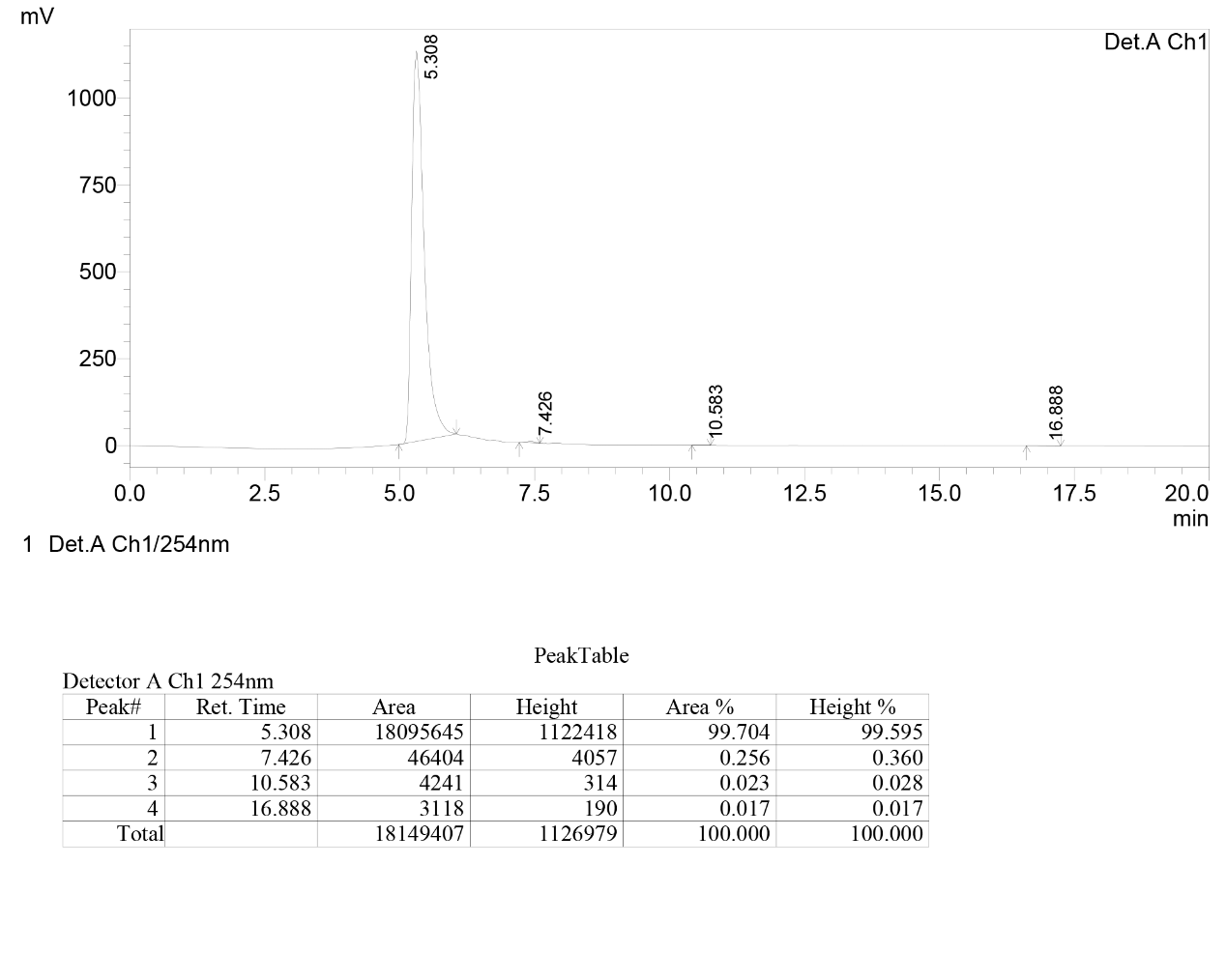


^1^H NMR spectrum of **B10** (400 MHz, CDCl_3_)





^13^C NMR spectrum of **B10** (101 MHz, CDCl_3_)





HPLC profile of **B10**


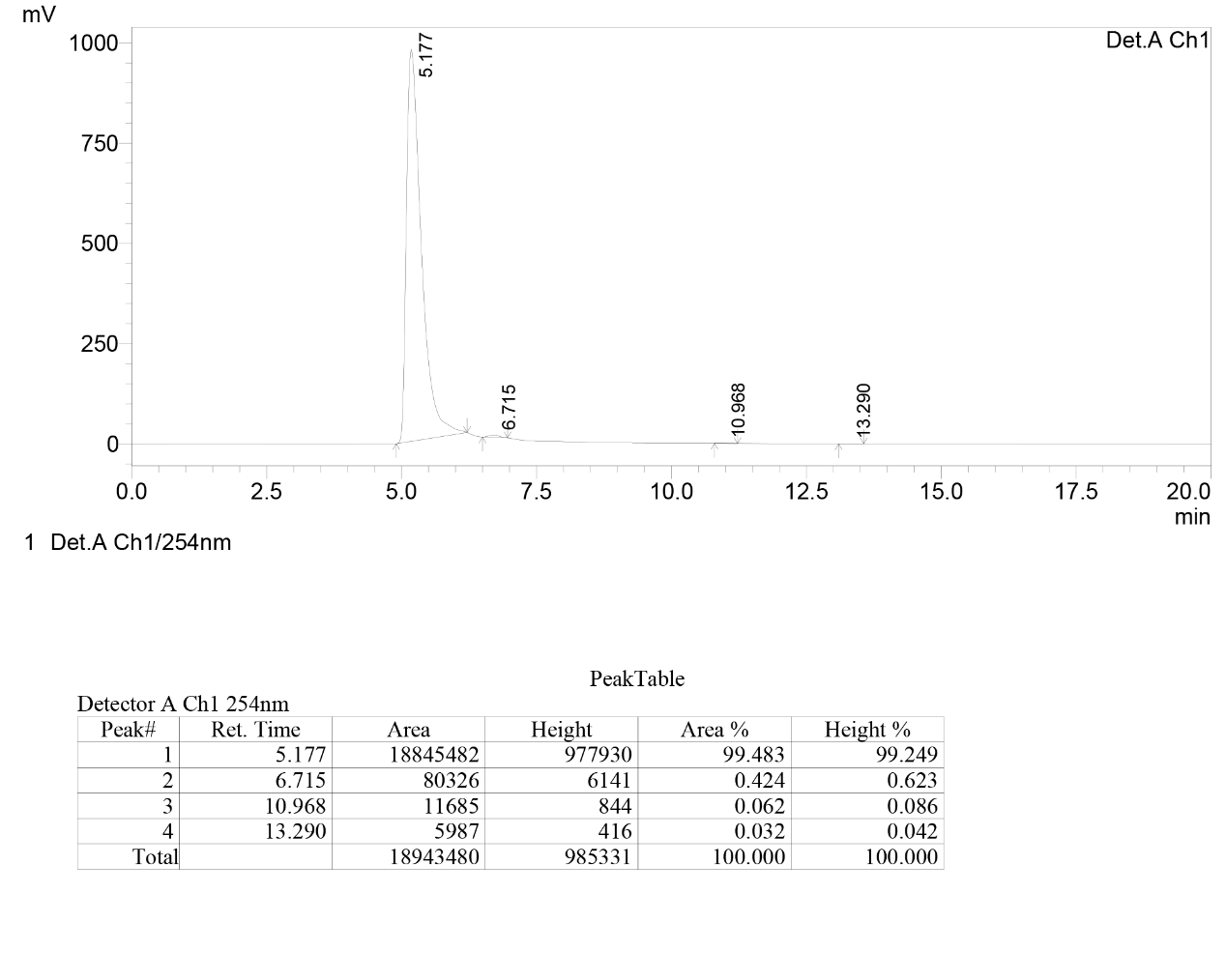


^1^H NMR spectrum of **B11** (400 MHz, DMSO)





^13^C NMR spectrum of **B11** (101 MHz, DMSO)





HPLC profile of **B11**

^1^H NMR spectrum of **B12** (400 MHz, CDCl_3_)

^13^C NMR spectrum of **B12** (101 MHz, CDCl_3_)

HPLC profile of **B12**

^1^H NMR spectrum of **B13** (400 MHz, CDCl_3_)

^13^C NMR spectrum of **B13** (101 MHz, CDCl_3_)

HPLC profile of **B13**

^1^H NMR spectrum of **B14** (400 MHz, DMSO)

^13^C NMR spectrum of **B14** (101 MHz, DMSO)

HPLC profile of **B14**

HPLC profile of **Curcumin**
